# Factors associated with circulating sex hormones in men: Individual Participant Data meta-analyses

**DOI:** 10.7326/M23-0342

**Published:** 2023-08-29

**Authors:** Ross J Marriott, Kevin Murray, Robert J Adams, Leen Antonio, Christie M Ballantyne, Douglas C Bauer, Shalender Bhasin, Mary L Biggs, Peggy M Cawthon, David J Couper, Adrian S Dobs, Leon Flicker, David J Handelsman, Graeme J Hankey, Anke Hannemann, Robin Haring, Benjumin Hsu, Magnus Karlsson, Sean A Martin, Alvin M Matsumoto, Dan Mellström, Claes Ohlsson, Terence W O'Neill, Eric S Orwoll, Matteo Quartagno, Molly M Shores, Antje Steveling, Åsa Tivesten, Thomas G Travison, Dirk Vanderschueren, Gary A Wittert, Frederick C W Wu, Bu B Yeap

**Affiliations:** 1 School of Population and Global Health, University of Western Australia, Perth, Australia; 2 Adelaide Institute for Sleep Health, Flinders University, Bedford Park, South Australia, Australia; 3 Laboratory of Clinical and Experimental Endocrinology, KU Leuven, Leuven, Belgium; 4 Internal Medicine, Baylor College of Medicine, Houston, Texas, USA; 5 General Internal Medicine, University of California, San Francisco, USA; 6 Brigham and Woman ’s Hospital, Harvard Medical School, Boston, USA; 7 Department of Biostatistics, School of Public Health, University of Washington, Seattle, Washington, USA; 8 San Francisco Coordinating Center, California Pacific Medical Center Research Institute, San Francisco, California, USA; 9 Gillings School of Global Public Health, University of North Carolina at Chapel Hill, Chapel Hill, North Carolina, USA; 10 School of Medicine, Division of Endocrinology, Diabetes and Metabolism, Johns Hopkins University, Baltimore, Maryland, USA; 11 Medical School, University of Western Australia, Perth, Australia; 12 Western Australian Centre for Healthy Ageing, University of Western Australia, Perth, Australia; 13 ANZAC Research Institute, University of Sydney, Sydney, Australia; 14 Perron Institute for Neurological and Translational Science, Perth, Australia; 15 Institute of Clinical Chemistry and Laboratory Medicine, University Medicine Greifswald, Greifswald, Germany; 16 DZHK (German Centre for Cardiovascular Research), Partner Site Greifswald, Greifswald, Germany; 17 School of Public Health and Preventive Medicine, Monash University, Melbourne, Australia; 18 European University of Applied Sciences, Faculty of Applied Public Health, Rostock, Germany; 19 Centre for Big Data Research in Health, University of New South Wales, Sydney, Australia; 20 Clinical and Molecular Osteoporosis Research Unit, Departments of Orthopedics and Clinical Sciences, Lund University, Skane University Hospital, Malmo, Sweden; 21 Australian Institute of Family Studies, Southbank, Australia; 22 Department of Medicine, University of Washington School of Medicine, Seattle, USA; 23 Geriatric Research, Education and Clinical Center, VA Puget Sound Health Care System, Seattle, USA; 24 Centre for Bone and Arthritis Research at the Sahlgrenska Academy, Institute of Medicine, University of Gothenburg, Goteborg, Sweden; 25 Centre for Epidemiology Versus Arthritis, University of Manchester and NIHR Manchester Biomedical Research Centre, Manchester University NHS Foundation Trust, Manchester, UK; 26 Oregon Health and Science University, Portland, USA; 27 MRC Clinical Trials Unit, University College London, London, UK; 28 School of Medicine, Department of Psychiatry and Behavioral Sciences, University of Washington, Seattle, Washington, USA; 29 Department of Internal Medicine, University Medicine Greifswald, Greifswald, Germany; 30 Wallenberg Laboratory for Cardiovascular and Metabolic Research, Department of Molecular and Clinical Medicine, Institute of Medicine, Sahlgrenska Academy, University of Gothenburg, Gothenburg, Sweden; 31 Department of Endocrinology, Sahlgrenska University Hospital, Region Västra Götaland, Gothenburg, Sweden; 32 Institute for Aging Research, Hebrew Senior Life, Beth Israel Deaconess Medical Center; Harvard Medical School, Boston, Massachusetts, USA; 33 Freemasons Centre for Men’s Health and Wellbeing, School of Medicine, University of Adelaide, Adelaide, Australia; 34 Division of Endocrinology, Diabetes & Gastroenterology, School of Medical Sciences, University of Manchester, Manchester, UK; 35 Department of Endocrinology and Diabetes, Fiona Stanley Hospital, Perth, Australia

**Keywords:** Testosterone, sex hormone-binding globulin, luteinising hormone, dihydrotestosterone, estradiol, body mass index, male ageing

## Abstract

**Background:**

Different factors modulate circulating testosterone in men, impacting interpretation of testosterone measurements.

**Purpose:**

Clarify factors associated with variations in sex hormone concentrations.

**Data sources:**

Systematic literature searches (to July 2019).

**Study selection:**

Prospective cohort studies of community-dwelling men with total testosterone measured using mass spectrometry.

**Data extraction:**

Individual participant data (IPD, 9 studies, n=21,074) and aggregate data (2 studies, n=4,075). Sociodemographic, lifestyle, health factors, total testosterone, sex hormone binding globulin (SHBG), luteinising hormone (LH), dihydrotestosterone (DHT) and estradiol concentrations were extracted.

**Data synthesis:**

Two-stage random-effects IPD meta-analyses found a non-linear association of testosterone with age, with negligible change among men aged 17-70 years (1SD increase: -0.27 nmol/L; CI=-0.71,0.18) and decreasing testosterone with age for men >70 years (-1.55 nmol/L; CI=-2.05,-1.06). Testosterone was inversely associated with BMI (1SD increase -2.42 nmol/L; CI=-2.70,-2.13). Testosterone concentrations were lower for men who: were married (-0.57 nmol/L; CI=-0.89,-0.26); undertook 75 minutes vigorous physical activity/week (-0.51 nmol/L; CI=-0.90,-0.13); former smokers (-0.34 nmol/L; CI=-0.55,-0.12); had hypertension (-0.53 nmol/L; CI=-0.82,-0.24), cardiovascular disease (-0.35 nmol/L; CI=-0.55,-0.15), cancer (-1.39 nmol/L; CI=-1.79,-0.99), or diabetes (-1.43 nmol/L; CI=-1.65,-1.22). SHBG was directly associated with age, and inversely associated with BMI. LH was directly associated with age in men >70 years.

**Limitations:**

Cross-sectional analysis, heterogeneity between studies and in timing of blood sampling, and imputation for missing data.

**Conclusion:**

Multiple factors are associated with variation in male testosterone, SHBG and LH concentrations. Reduced testosterone and increased LH may indicate impaired testicular function after age 70 years. Interpretation of individual testosterone measurements should account particularly for age >70 years, obesity, diabetes and cancer.

**Primary funding sources:**

Medical Research Future Fund; Government of Western Australia; Lawley Pharmaceuticals.

**Registration:**

PROSPERO: CRD42019139668

## Introduction

Lower testosterone concentrations are associated with a range of poor health outcomes in ageing men, including higher risks of diabetes, dementia, and death, with some evidence for causation with respect to diabetes (1–4). However, it remains unclear whether declining testosterone concentrations are intrinsic to male ageing via structural deterioration of the hypothalamic-pituitary-testicular (HPT) axis or reflect functional inhibition resulting from age-related comorbidities (5,6). Some older men maintain circulating testosterone concentrations comparable to younger men (7), but testosterone concentrations even in very healthy older men as a group are lower than in healthy young men (8,9). The considerable variation in testosterone concentrations within and across age strata (10) may impact upon the application of testosterone reference ranges to assist in the diagnosis of male hypogonadism (11–14).

Sociodemographic, lifestyle and behavioural factors have been associated with differences in testosterone concentrations, as have medical comorbidities, in previous individual studies with uncertainty over the consistency and magnitude of such associations (5,6,15–18). Several previous studies assayed testosterone concentrations using immunoassays, rather than using mass spectrometry which provides more accurate results (19,20). Mass spectrometry also offers greater accuracy and precision than immunoassays for the active metabolites of testosterone, dihydrotestosterone (DHT, a ligand for the androgen receptor) and estradiol (a ligand for estrogen receptors, which mediates the action of testosterone in organs such as bone), both present in men in much lower concentrations than testosterone (8,21). However, there are limited studies exploring age-related changes in DHT and estradiol concentrations measured by mass spectrometry in men. Even the cohort studies that have measured sex hormones using mass spectrometry have had limited capacity to generalise the findings across different age strata or other geographic regions (5,6,8,17,22,23).

To better understand the relationship of circulating testosterone concentrations with age, and with other sociodemographic, lifestyle, and medical factors, in men of varying ages from around the world, we conducted the first individual participant data (IPD) meta-analyses of all major cohort studies that measured testosterone by mass spectrometry in community-dwelling men. By obtaining, checking and harmonising raw data from studies selected via a systematic review, and using pre-specified, highly flexible non-linear models, this approach facilitated descriptions of trends in adult men and enabled more precise estimates of associations with specific factors, relevant to men across different regions. Thus, these factors would be important to consider when interpreting testosterone results from individual men. Population, exposure, and outcomes characteristics included: men in the general community; sociodemographic, lifestyle, and prevalent health status factors (predictor variables); and endogenous circulating total testosterone, DHT and estradiol, all measured using mass spectrometry, luteinising hormone (LH, the pituitary hormone stimulating testicular testosterone production), and sex hormone-binding globulin (SHBG, the primary carrier protein for testosterone in the circulation) (dependent variables).

## Methods

The Androgens In Men Study (AIMS) protocol was submitted to PROSPERO (23 July 2019), registered (20 November 2019; CRD42019139668) and published (24,25). Cross-sectional random effects Individual Participant Data Meta-Analyses (IPDMAs) were performed because variation in effect estimates among studies were assumed attributable, at least in part, to differences in local factors (26). A PRISMA-IPD reporting checklist is included (Supplementary Table S1). This analysis was approved by the Human Research Ethics Office of the University of Western Australia.

### Data sources and searches

A systematic review (to July 2019) identified prospective cohort studies (25). Details of the original search and a bridge search to May 2023 are provided (Supplementary Material).

### Study selection

Eligible studies were prospective cohort studies of community-dwelling adult men with total testosterone concentrations measured using mass spectrometry and ≥5 years follow-up for specific health outcomes (24). 11 suitable studies were identified from the systematic review, nine provided IPD-level data (27–39), and two provided aggregate data statistics (AD) (40,41). A flow chart and summary attributes are presented (Supplementary Fig. S1; Appendix Table A1). Further details on the systematic review, including all methods, PRISMA flow chart, attributes of selected items, and preliminary meta-analyses of published estimates, were reported (25).

### Data extraction and quality assessment

Variables for planned IPDMAs were agreed in advance (Supplementary Table S2) (24). The Newcastle-Ottawa Quality Assessment scale was used (Supplementary Material). Datasets from individual studies were securely sent, stored in a central repository, and checked (Supplementary Methods). IPD-level data were provided by nine studies for 17 requested variables, with nine additional variables provided by only some studies but deemed satisfactory for analysis (Supplementary Table S2). Rules were devised for harmonisation (Supplementary Table S3). No other important issues were identified in checking IPD.

#### Sex hormones

Total testosterone (nmol/L), DHT (nmol/L) and estradiol (pmol/L) were measured using mass spectrometry, testosterone in all and DHT and estradiol in some studies. SHBG (nmol/L) and LH (IU/L) were measured using immunoassays. Equilibrium dialysis for measurement of testosterone not bound to SHBG or other binding proteins had not been performed. Further details were documented for each respective study (25). Cohort recruitment criteria are summarized, with most studies collecting blood samples in the morning (Appendix Methods, Supplementary Table S4A).

#### Sociodemographic and lifestyle variables

Participant age (years) and body mass index (kg/m^2^) at time of blood sampling for testosterone assay (baseline) were provided or calculated from provided variables (Appendix Table A1). Education status was harmonised as attained university degree or equivalent (yes/no) and marital status as married or in a de facto relationship (yes/no). Alcohol consumption and duration of vigorous physical activity were harmonisation using thresholds of 19.5 g/day and 75 min/week. Smoking status was categorised as Never/Former/Current. Reference values (continuous variables), reference levels (categorical factors), and the rationale for harmonisation rules are provided (Supplementary Tables S2-S3).

#### Prevalent health and medical conditions

General health status was harmonised as Good/Excellent (yes/no), and drug use status (lipid-lowering medications, psychotropic drugs) was either supplied or derived using ATC codes or by reviewing lists of medications used. If status of a health condition was not supplied, additional information was used (e.g. for diabetes status: medication usage, fasting glucose, or HbA1c measurements). Health condition definitions (e.g. for hypertension, cancer, CVD, chronic obstructive pulmonary disease [COPD]), including International Classification for Diseases (ICD)-9 and ICD-10 codes are presented (Supplementary Table S3).

### Data synthesis and analysis

The two-stage IPDMA approach was adopted, to facilitate analysis of studies with IPD and also studies where only AD were available (42). This fits the same statistical model to IPD from each study separately (Stage 1) and then combines estimates from the fitted models (study-specific coefficient estimates and covariance matrices) in a random-effects meta-analysis (Stage 2). IPDMAs were firstly applied to the full set of analyses using the nine supplied IPD-level datasets. Analyses of the IPD-level datasets were given precedence because it was possible for a more comprehensive appraisal of data quality, risk of bias, and model fit diagnostics, as compared with supplied AD (42). AD from two additional studies (supplied coefficient estimates and covariance matrices) were used in a sensitivity analysis, to see if their inclusion affected results. In the sensitivity analysis, IPDMAs were repeated, with the inclusion of those two additional sets of AD in Stage 2 (for models including sociodemographic and lifestyle predictors, and prevalent health conditions of CVD and diabetes: Supplementary Methods). Analyses were performed in R version 4.0.2.

Cross-sectional IPDMAs involved modelling relationships between predictors of interest (independent variables, IVs) and dependent variables (total testosterone, SHBG, LH, DHT, estradiol concentrations, DVs). Estimates of associations were presented as marginal effects calculated from a series of pre-specified multivariable models that were fitted to IPD (Appendix Table A2). Analyses show the estimated association of each hormone with each: (i) sociodemographic predictor controlled for all other sociodemographic predictors in Model 1; (ii) lifestyle predictor controlled for all other lifestyle and all sociodemographic predictors in Model 2; and (iii) prevalent health condition controlled for all sociodemographic and lifestyle predictors in Models 3-16.

Summary estimates for associations between each hormone variable and predictor of interest are presented in tables and graphically in summary curves (continuous predictors) or forest plots (categorical predictors). Measures of effect size are mean difference (MD) for an increase in one SD around the reference value (for continuous variable, Supplementary Table S5) or MD compared to the reference level (presence vs absence for categorical variable). Full details are provided including methods for imputation of missing data (Appendix Methods, Supplementary Methods, Supplementary Tables S2, S5 & S6).

The relative extent of heterogeneity was quantified using *I*^2^ (43). 95% confidence intervals (CIs) of *I*^2^ were also reported, and the range of effect sizes reported where there was appreciable relative heterogeneity (i.e. *I*^2^ CI >50%; Supplementary Methods). Contour-enhanced funnel plots were constructed to explore the prospect for publication bias. The sensitivity of results to ethnicity type was explored in subgroup analyses (Supplementary Results). Prediction intervals are provided showing estimates of the interval containing the true effect for a potential new cohort study, with 95% probability (44).

### Funding sources

Are detailed in the Appendix.

## Results

Excluding men with prior orchidectomy (n=64), using androgens/anti-androgens (n=287) or without testosterone measurements (n=6,501), there were IPD for n=21,074 participants from nine studies and AD statistics for n=4,075 from two studies (Supplementary Fig. S1). Median ages ranged from 49-76 years, and median testosterone concentrations from 12.4-20.4 nmol/L (Appendix Table A1). Testosterone and SHBG measurements were available in all 11 studies. LH, DHT and estradiol measurements were available in 6, 7, and 9 studies, respectively. Studies were generally of high quality with scores (total stars) from Newcastle-Ottawa Quality Assessments ranging from six to nine (25). The bridge search revealed another two potentially eligible cohorts involving 4,366 men (Supplementary Methods, Supplementary Table S4B).

### Associations with sociodemographic factors (Model 1)

**Figure 1:**
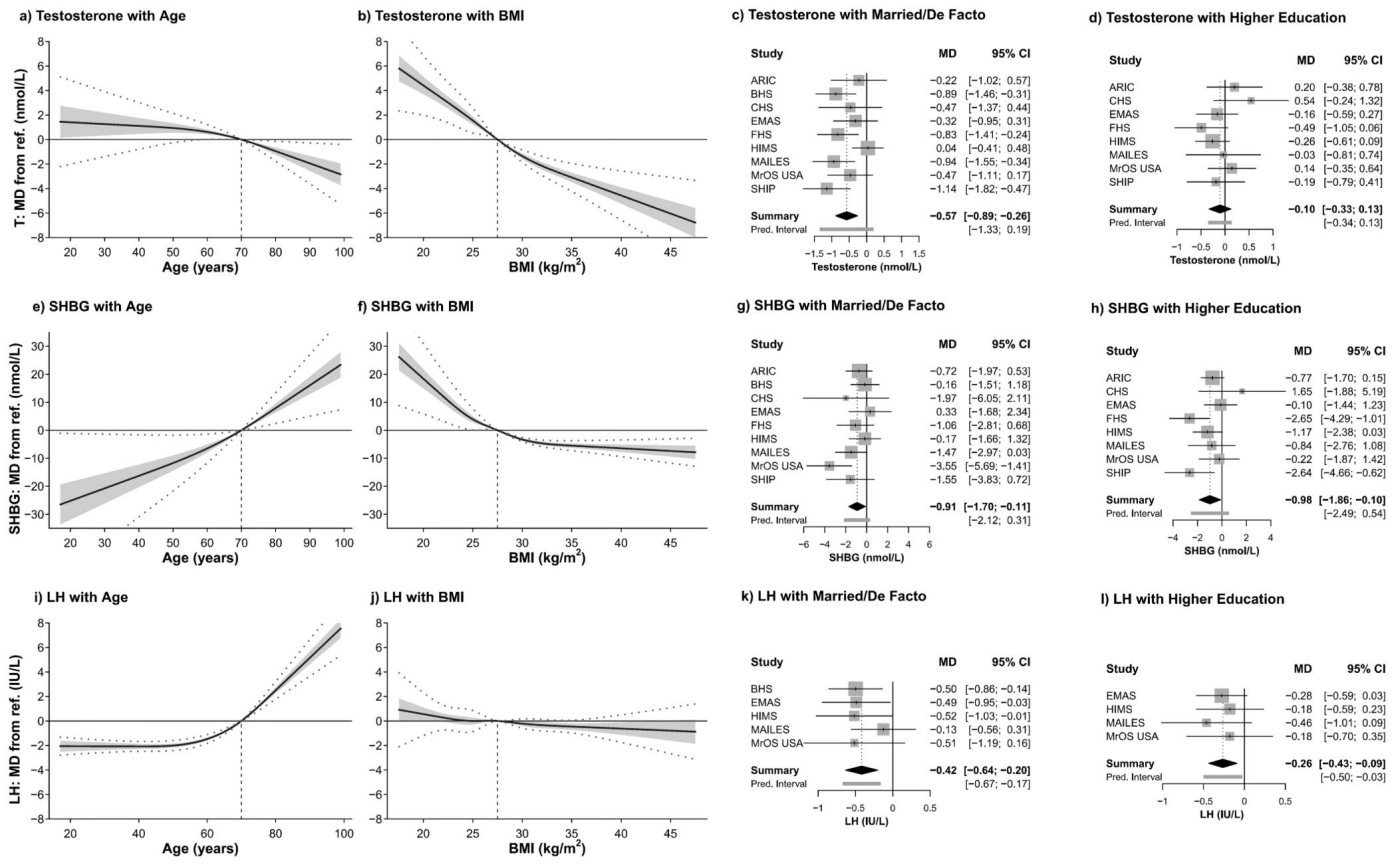
Summary curves and forest plots for the associations of sociodemographic factors with testosterone, SHBG, and LH concentrations after controlling for all other sociodemographic predictors in Model 1 (refer Appendix Table A1). MD = mean difference; vertical dashed line on summary curves identifies the reference level (ref.) for the predictor of interest; dotted lines show 95% prediction intervals; forest plots show the MD from the reference level of the categorical predictor (refer Supplementary Tables S2, S3). MD=mean difference, CI=confidence interval, T=testosterone, SHBG=sex hormone-binding globulin, LH=luteinising hormone, BMI=body mass index, Pred. interval=prediction interval. ARIC=Atherosclerosis Risk in Communities Study, BHS=Busselton Health Study, CHS=Cardiovascular Health Study, EMAS=European Male Ageing Study, FHS=Framingham Heart Study, HIMS=Health In Men Study, MAILES=Men Androgen Inflammation Lifestyle Environment and Stress study, MrOS USA=Osteoporotic Fractures in Men USA study, SHIP=Study of Health in Pomerania SHIP.

Model 1 includes adjustment for sociodemographic factors (age, BMI, marital status and education). Testosterone decreased with age, while SHBG and LH increased, with no overall differences in DHT or E2 (Table 1). However, the association of testosterone with age was non-linear, with negligible change among men aged 17-70 years, and an inverse association in men >70 years (Fig. 1a). The change in mean testosterone per SD increase about the mid-point of age range 17-70 years (1SD increase about age 43.5, from 35.7-51.3 years) was -0.27 nmol/L (CI=-0.71,0.18) compared to 70-99 years -1.55 nmol/L (CI=-2.05,-1.06, for 1SD increase about age 84.5, from 76.7-92.3 years). Similarly, men who were >70 years old demonstrated steeper increases in SHBG and LH with age (Fig. 1e,i). There was little change in mean LH with age in men <70 years (per SD increase 0.10 IU/L, CI=-0.08,0.28), but an increase with age in men ≥70 years (per SD increase 4.14 IU/L, CI=3.71,4.56) (Fig. 1l). Although there was no overall difference (Table 1), mean estradiol increased with age in men <70 years, but not older men (Supplementary Fig. S2e).

Testosterone was inversely associated with BMI (1SD increase about 27.5 kg/m^2^ from 25.5-29.6 kg/m^2^ -2.42 nmol/L, CI=-2.70,-2.13), as were SHBG and DHT (Table 1). The association of SHBG with BMI was non-linear, becoming less steep for BMI >27.5 kg/m^2^ (Fig. 1f). Similarly, only men with BMI >32 kg/m^2^ had higher estradiol concentrations (Supplementary Fig. S2f). Men who were married/in a de facto relationship had lower mean testosterone (-0.57 nmol/L, CI=-0.89,-0.26), SHBG (-0.91 nmol/L, CI=-1.70,-0.11), LH (-0.42 IU/L, CI=-0.64,-0.20) and estradiol (-4.9 pmol/L, CI=-8.7,-1.2), with no difference in DHT (Table 1; Fig. 1c,g,k; Supplementary Fig. S2c,g). Men with higher education level had lower SHBG (-0.98 nmol/L, CI=-1.86,-0.10), LH (-0.26 IU/L, CI=-0.43,-0.09) and DHT (-0.03 nmol/L, CI=-0.05,-0.01), with no difference in testosterone or estradiol (Table 1; Fig. 1d,h,i; Supplementary Fig. S2d,h).

Estimates of *I*^2^ showing variable relative heterogeneity for associations of sex hormones with different factors and descriptions of the prediction intervals are provided for these and subsequent analyses (Appendix Results, Appendix Table A3).

### Associations with lifestyle factors (Model 2)

Model 2 includes adjustment for all sociodemographic factors in Model 1, and for lifestyle factors (alcohol consumption, physical activity, smoking status). Frequent drinkers had lower mean SHBG (-1.53 nmol/L, CI=-2.49,-0.57), with no differences in testosterone, LH, DHT or estradiol (Table 1, Supplementary Fig. S4a,e,i; Supplementary Fig. S5a,e). Testosterone was lower in men undertaking ≤75 minutes vigorous physical activity/week (-0.51 nmol/L, CI=-0.90,-0.13) as was SHBG (-0.66 nmol/L, CI=-1.20,-0.12) with no differences in LH, DHT or estradiol (Table 1; Supplementary Figs. S4 b,f,j & S5b,f). Current smokers had higher mean testosterone (0.89 nmol/L, CI=0.36,1.42), SHBG (4.32 nmol/L, CI=2.72,5.90) and LH (0.57 IU/L, CI=0.37,0.77) compared to never-smokers (Table 1; Supplementary Fig. S4d,h,l), with no differences in DHT or estradiol (Supplementary Fig. S5d,h). Former smokers had lower mean testosterone (-0.34 nmol/L, CI=-0.55,-0.12), SHBG, DHT and estradiol versus never-smokers (Table 1; Supplementary Fig. S4c,g,k & S5c,g).

### Associations with prevalent health and medical conditions (Models 3-16)

**Figure 2:**
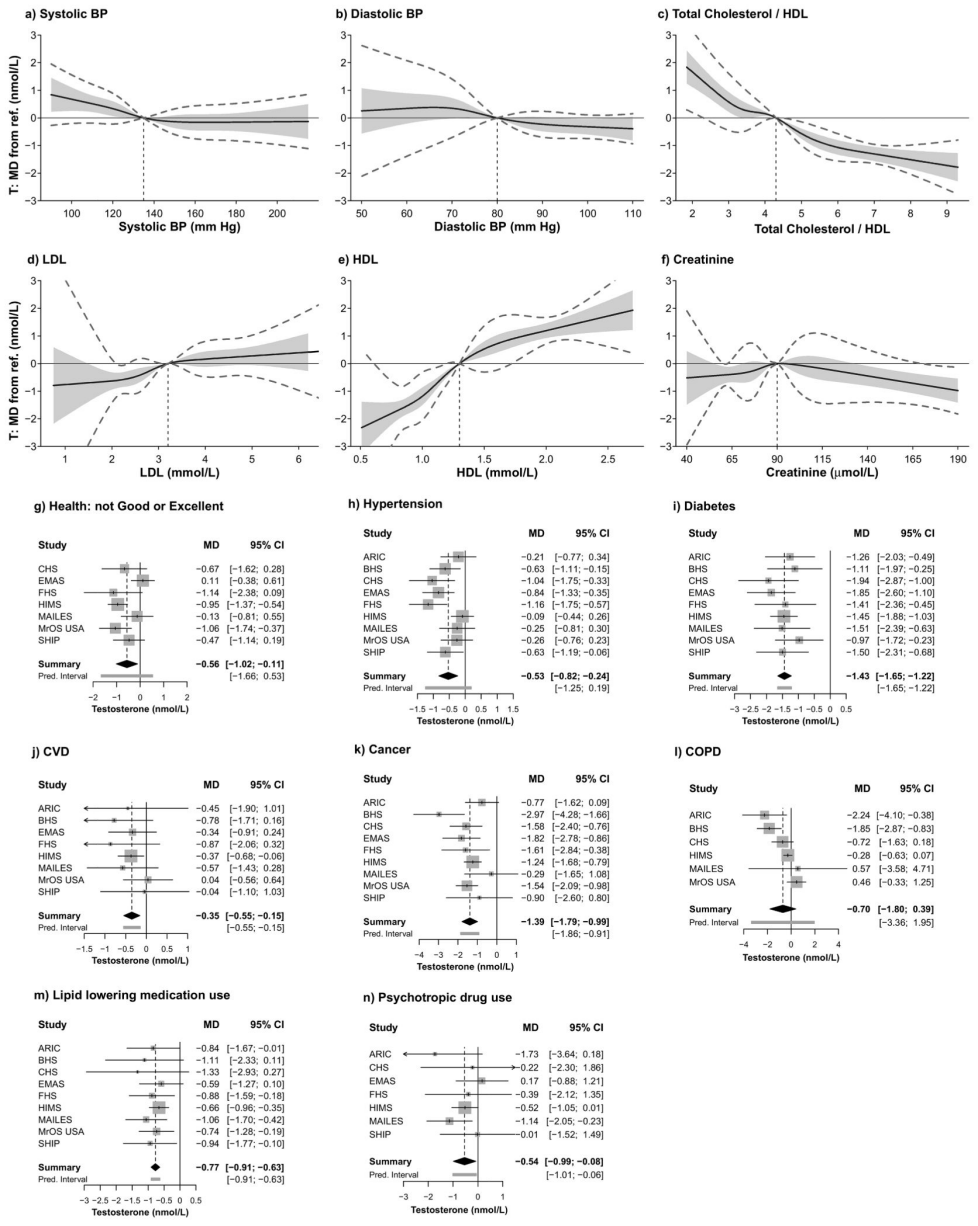
Summary curves and forest plots for the associations of prevalent health conditions with testosterone concentration after controlling for all sociodemographic and lifestyle predictors (refer Appendix Table A1). MD = mean difference; vertical dashed line on summary curves identifies the reference level (ref.) for the predictor of interest; dotted lines show 95% prediction intervals; forest plots show the MD from the reference level of the categorical predictor (refer Supplementary Tables S2, S3). MD=mean difference, T=testosterone, BP=blood pressure, HDL=high density lipoprotein, LDL=low density lipoprotein, CVD=cardiovascular disease, COPD=chronic obstructive pulmonary disease, CI=confidence interval, Pred. interval=prediction interval. ARIC=Atherosclerosis Risk in Communities Study, BHS=Busselton Health Study, CHS=Cardiovascular Health Study, EMAS=European Male Ageing Study, FHS=Framingham Heart Study, HIMS=Health In Men Study, MAILES=Men Androgen Inflammation Lifestyle Environment and Stress study, MrOS USA=Osteoporotic Fractures in Men USA study, SHIP=Study of Health in Pomerania SHIP.

Models 3-16 adjust for all sociodemographic and lifestyle predictors shown in Models 1 and 2. Higher diastolic blood pressure (BP) was associated with lower testosterone (-0.40 nmol/L, CI=-0.72,-0.08 nmol/L), SHBG and LH, higher systolic BP with lower testosterone (-0.35 nmol/L, CI=-0.61,-0.08), and hypertension with lower testosterone (-0.53 nmol/L, CI=-0.82,-0.24) and SHBG, and not with other hormones (Table 1, Fig. 2a,b, Supplementary Figs. S6-S9a,b). Men with Fair/Poor/Very Poor self-rated general health had lower testosterone (-0.56 nmol/L, CI=-1.02,-0.11), and higher SHBG and LH, with no differences in DHT or estradiol (Table 1, Fig. 2g, Supplementary Figs. S6-S9g).

Men with CVD had lower testosterone (-0.35 nmol/L, CI=-0.55,-0.15) with no difference in SHBG or other hormones, while COPD was not associated with any hormones (Table 1, Fig. 2j,l, Supplementary Figs. S5-S8j,l). Men with cancer had lower testosterone (-1.39 nmol/L, CI=-1.79,-0.99), higher LH, and lower DHT and estradiol, with no difference in SHBG (Table 1, Fig. 2k, Supplementary Figs. S6-S9k). Men with diabetes had lower testosterone (-1.43 nmol/L, CI=-1.65,-1.22), SHBG, DHT and marginally lower estradiol, with no difference in LH (Table 1, Fig. 2i, Supplementary Figs. S6-S9i).

Across the range of values, total cholesterol to HDL ratio was inversely associated, and LDL and HDL directly associated, with testosterone, SHBG and DHT, with no differences for LH and estradiol (Table 1, Fig. 2c,d,e, Supplementary Figs. S6-S9c,d,e). However, there were non-linear associations within these overall trends. Estradiol was inversely associated with total cholesterol to HDL ratio when the ratio was <2.75 (Supplementary Fig. S9c). Men with higher creatinine had lower SHBG and higher estradiol, testosterone was positively associated for creatinine 55-71 μmol/L, while testosterone and DHT were inversely associated for creatinine >136 μmol/L (Table 1, Fig. 2f, Supplementary Figs. S6-S9f). LH was higher in men with LDL <1.9 mmol/L or creatinine >90 umol/L (Supplementary Fig. S7d,f). Men taking lipid-lowering medications had lower testosterone (-0.77 nmol/L, CI=-0.91,-0.63), SHBG, DHT and estradiol concentrations; while men on psychotropic drugs had lower testosterone (-0.54 nmol/L, CI=-0.99,-0.08) and estradiol concentrations, without other associations (Table 1, Fig. 2m,n, Supplementary Figs. S6-S9m,n).

### Other analyses

Sensitivity analyses including examining the effect of imputing missing data, and bias assessments did not substantively alter the findings (Supplementary Methods, Supplementary Results, Supplementary Figs. S11-S19). Incorporating AD from two additional studies resulted in slight differences to summary estimates and heterogeneity but these differences did not substantively change results (Fig. 3).

### Exploratory analyses

Additional adjustment by controlling for lifestyle factors, and for prevalent CVD or diabetes, did not substantively change the summary estimates for associations of sociodemographic factors including age and BMI with total testosterone (Appendix Table A4). In subgroup analyses (not pre-specified) excluding men with hypertension, diabetes, CVD, cancer, COPD, on lipid-lowering medications or with serum creatinine >150 μmol/L, the decline in testosterone in men >70 years was attenuated, while the increase in LH in men >70 years was unchanged (Supplementary Results, Supplementary Figs. S20, S21).

## Discussion

While other individual studies have reported associations of sociodemographic, lifestyle and medical factors with testosterone concentrations (5,6,15–18), this is the first meta-analysis involving all major cohort studies with testosterone measured using mass spectrometry (24,25). Our IPDMAs provide a unique opportunity to draw conclusions regarding circulating testosterone, accurately measured using mass spectrometry, relevant to men across the lifespan from diverse regions of the world. Additional novel insights are provided by the parallel IPDMAs of SHBG and LH, and mass spectrometry-measured DHT and estradiol, which show both contrasting and consistent associations with factors influencing circulating testosterone.

In men aged 17-99 years from around the world, mean testosterone concentrations did not differ with age until ≥70 years. Above this age testosterone concentrations declined by ~1.6 nmol/L per 15.6 years, while LH increased with age. The decline in testosterone after age 70 years was less apparent in the subgroup of men free of hypertension, diabetes, CVD, cancer, COPD, lipid-lowering medications or elevated creatinine. Higher BMI was associated with mean testosterone concentration ~2.5 nmol/L lower (per 4.1 kg/m^2^). The presence of either diabetes or cancer was associated with mean testosterone concentrations ~1.5 nmol/L lower, and being married, less physically active, self-reporting poorer health, having hypertension or CVD, or use of lipid-lowering or psychotropic medications, were each associated with mean testosterone concentrations ~0.5 nmol/L lower.

While SHBG increased across the age span, testosterone and LH were stable until after age 70 years, whereupon divergent associations of testosterone and LH with age emerged. The magnitude of the age-associated increase in SHBG was pronounced, and further investigation is warranted to explore whether this might alter the bioavailability of testosterone to access target tissues. Previous studies limited to men ≥70 years have reported longitudinal declines in testosterone concentrations and increases in LH with age (45,46). Our IPDMA, including data from men aged 17-99 years, provides new evidence suggesting that a change in HPT axis function may occur in men around age 70 years. The relative stability of mean testosterone until, and the decline after this age, raises the question whether a single reference range should be applied across men of all ages. A reference range for healthy nonobese young men has been proposed (9.2-31.8 nmol/L based on 2.5^th^-97.5^th^ percentiles in men aged 19-39 years, for assays standardised to a higher order reference method established by the Centers for Disease Control and Prevention) (12). It may be appropriate to adjust the lower cut-off when applying this to older men. Alternatively, an age-appropriate reference range has been proposed for men ≥70 years (6.4-25.7 nmol/L based on 2.5^th^-97.5^th^ percentiles in very healthy older men) (8,11).

Longitudinal data from the European Male Ageing Study associated age and poorer health with the transition to lower testosterone and higher LH concentrations (47). In our cross-sectional analysis, in the subgroup of men without common medical comorbidities LH was directly associated with age after 70 years. The observed epidemiological trend is consistent with Leydig cell impairment in older men, but further research is needed to determine whether, and if so what proportion of older men might have organic hypogonadism due to testicular damage or atrophy.

Higher BMI was associated with lower mean testosterone, DHT and SHBG, with marginal difference in LH. The magnitude of the inverse association between BMI and mean testosterone concentrations was substantial, with narrow confidence intervals, and was consistent across the range of BMI, reflecting the contributions of central adiposity and insulin resistance to lower total testosterone concentrations (48). The inverse association of SHBG with BMI has been related to underlying central adiposity, with insulin resistance and/or hepatic lipogenesis affecting liver synthesis of SHBG (48). We found that this association was non-linear, the gradient becoming shallower with BMI values >30 kg/m^2^. Therefore, at higher BMI values, lower SHBG may not in itself account for lower mean testosterone concentrations. An association of BMI with higher estradiol concentrations (reflecting aromatisation of testosterone within adipose tissue) was only found in men with BMI >32 kg/m^2^.

Being married, or in a de facto relationship, was associated with lower mean testosterone, SHBG, LH and estradiol, to a lesser magnitude than seen with BMI. We noted a similar finding in UK Biobank men for testosterone measured with immunoassay, and SHBG, being lower in men with a partner (18). The postulated explanation was this might reflect stresses of family life, including children in the household. There was heterogeneity in the estimates, the association being strongest in cohorts with middle-aged men (BHS, FHS, MAILES, SHIP) and less apparent in cohorts with older men (ARIC, CHS, EMAS, HIMS, MrOS USA). Therefore, the IPDMA result confirms the association of marriage (or similar long-term relationship) with lower testosterone concentrations, which is independent of age, but less prominent in older men.

Men who were less physically active had lower testosterone and SHBG. Current smokers had higher mean testosterone, SHBG and LH, and ex-smokers lower testosterone, SHBG, DHT and estradiol, compared with never-smokers. While these are cross-sectional associations, and the possibility of confounding from unmeasured variables or reverse causation exists, a plausible explanation would be that differences are driven primarily via changes in SHBG, although the higher LH in current smokers suggests possible modulation of the HPT axis. Men who self-reported poorer health had lower mean testosterone, and higher SHBG and LH. Testosterone and SHBG were inversely associated with systolic BP; testosterone, SHBG and DHT were inversely associated with the ratio of cholesterol to HDL; and directly associated with HDL and LDL, generally consistent with an association of higher sex hormones and SHBG with favourable cardiovascular risk markers. Of note, diabetes and cancer were associated with the largest differences in mean testosterone. Men with diabetes had lower testosterone, SHBG, DHT and estradiol. By contrast, men with cancer had lower testosterone, DHT and estradiol but higher LH, suggestive of testicular impairment in this setting.

The size of our IPDMA analysis population enabled us to estimate the associations of specific sociodemographic, lifestyle and medical factors with differences in mean testosterone concentrations with high precision. These findings may be relevant for the evaluation of men with suspected hypogonadism. Androgen deficiency is a clinical syndrome, whose diagnosis is based on the presence of indicative symptoms and signs, with confirmatory biochemical testing requiring interpretation of results (11–14). However, differences in testosterone concentrations attributable to various factors, including those which are potentially reversible, need to be accounted for. In any individual man, sociodemographic, lifestyle and medical factors should be considered when interpreting a testosterone result, particularly when that result is closer to the lower bound of the reference interval. These factors should also be considered as potential confounders in analyses evaluating the associations of testosterone concentrations with health outcomes in men.

Strengths of this work include the inclusion of 11 major prospective cohort studies, all of which used mass spectrometry to assay testosterone concentrations, in IPDMAs. In some studies, the low concentrations of DHT and estradiol found in men were also measured more precisely and accurately using mass spectrometry assays. The combined dataset represents many men across the span of ages, from different geographic regions of the world (27–41). Consistent and clear associations were identified, particularly for testosterone, SHBG and LH. Limitations of the work include its cross-sectional nature precluding determination of causation. Two of the 11 studies provided AD rather than IPD, accommodated into the structure of the two-stage IPDMA. As some variables were recorded differently across studies, these were categorised to enable data to be harmonised. The possibility of confounding from unmeasured variables and reverse causation cannot be excluded. Across all IPDMAs, the percentage of cases with missing values was sufficient to warrant imputation, with the additional benefits of maximising available data and statistical power, and imputing key variables when completely missing. The validity of imputations was contingent upon the assumption that missingness was conditional upon observed data, within and between the studies.

Whilst testosterone, and in some cohorts DHT and estradiol, were all assayed using mass spectrometry, these were performed in different laboratories at different times, which may have contributed to the observed degree of heterogeneity. However, mass spectrometry is the gold standard and should provide greater consistency than would be the case with a range of different immunoassays (9,19). Calculation of free testosterone was outside the scope of the current work. There was considerable heterogeneity in the estimates, nevertheless the findings across cohorts were generally consistent. Most studies, but not all, collected morning blood samples, which might have contributed to the observed heterogeneity. While two additional cohorts were identified in the bridge search, they would have to be approached for data to determine eligibility. Given the number of participants involved compared with the analysed 11 cohorts the results of a future IPDMA including these would likely be similar. Men within the combined dataset were primarily of White ethnicity, from Australia, Europe and North America, hence our results require confirmation in men of other ethnicities, and men from South America, Africa and Asia.

In conclusion, multiple factors are associated with variation in male testosterone, SHBG and LH concentrations, with evidence of primary impairment of testicular hormone production after age 70 years. Interpretation of individual testosterone measurements should account particularly for age >70 years, higher BMI, and the presence of diabetes or cancer. Additional research is needed to determine mechanisms underlying the association of marriage with lower testosterone concentrations in middle-aged men, and the implications of impaired Leydig cell function for health of older men.

## Supplementary Material

Appendix

Supplementary material

## Figures and Tables

**Figure 3:**
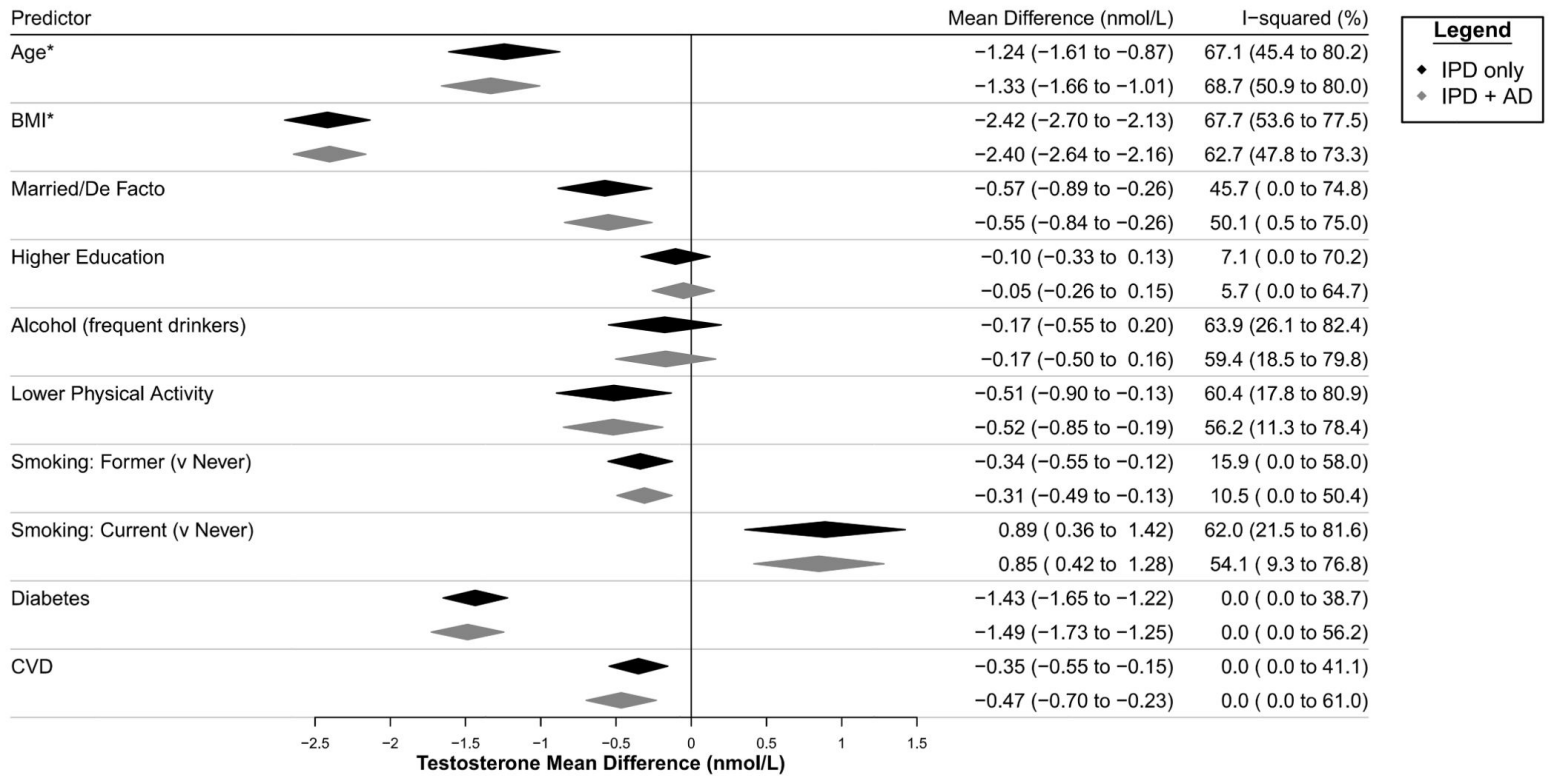
Sensitivity of summary estimates (IPD only: for Models 1, 2, 7 and 10) to the inclusion of aggregate level data (IPD + AD) provided by two additional studies. Summary estimates show the mean difference from the reference level of the categorical predictor. * = summary estimates presented as change for 1 standard deviation increase around the Ref. value (Supplementary Table S5). BMI=body mass index.

**Table 1:** Summary effect sizes describing cross-sectional associations between androgen concentration and sociodemographic, lifestyle, health and medication factors from meta-analyses of multiply-imputed individual participant data.

Model	Predictor	Level^b^	Effect size^c^				
			Testosterone (mnol/L)	SHBG (mnol/L)	LH (IU/L)	DHT (mnol/L)	Estradiol (pmol/L)
*Social/demographic predictors*							
1	Age^a^		-1.24 (-1.61 to -0.87)	11.33 (9.04 to 13.62)	3.16 (2.86 to 3.46)	-0.06 (-0.16 to 0.05)	2.66 (-1.69 to 7.02)
1	BMI^a^		-2.42 (-2.70 to-2.13)	-5.92 (-6.88 to -4.95)	-0.17 (-0.40 to 0.05)	-0.29 (-0.34 to -0.25)	0.40 (-0.79 to 1.59)
1	Married or de facto:	Yes	-0.57 (-0.89 to -0.26)	-0.91 (-1.70 to-0.11)	-0.42 (-0.64 to -0.20)	-0.03 (-0.10 to 0.05)	-4.94 (-8.70 to-1.18)
1	Higher education:	Yes	-0.10 (-0.33 to 0.13)	-0.98 (-1.86 to-0.10)	-0.26 (-0.43 to -0.09)	-0.03 (-0.05 to -0.01)	-1.18 (-3.48 to 1.12)
*+ Lifestyle predictors*							
2	Alcohol consumed:	≥19.2g/d	-0.17 (-0.55 to 0.20)	-1.53 (-2.49 to-0.57)	-0.38 (-0.82 to 0.05)	-0.02 (-0.06 to 0.01)	0.77 (-0.91 to 2.45)
2	Physical activity^d^	≤75min	-0.51 (-0.90 to-0.13)	-0.66 (-1.20 to-0.12)	0.05 (-0.37 to 0.47)	-0.04 (-0.09 to 0.02)	-0.38 (-1.85 to 1.09)
2	Smoking (vs Never):	Former	-0.34 (-0.55 to-0.12)	-0.61 (-1.10 to-0.12)	0.09 (-0.19 to 0.37)	-0.07 (-0.10 to-0.03)	-3.35 (-5.96 to -0.73)
		Current	0.89 (0.36 to 1.42)	4.31 (2.72 to 5.90)	0.57 (0.37 to 0.77)	0.03 (-0.18 to 0.23)	-0.78 (-3.02 to 1.47)
*+ Prevalent health*							
3	Diastolic BP^a^		-0.40 (-0.72 to -0.08)	-0.99 (-1.86 to-0.12)	-0.35 (-0.55 to-0.14)	0.02 (-0.02 to 0.06)	0.36 (-1.34 to 2.07)
4	Systolic BP^a^		-0.35 (-0.61 to -0.08)	-0.41 (-1.10 to 0.28)	0.09 (-0.14 to 0.31)	0.01 (-0.03 to 0.04)	0.68 (-0.81 to 2.17)
5	Hypertension:	Yes	-0.53 (-0.82 to -0.24)	-1.31 (-2.34 to-0.28)	0.05 (-0.18 to 0.29)	-0.05 (-0.11 to 0.01)	0.40 (-1.12 to 1.91)
6	General health:	<Good^e^	-0.56 (-1.02 to -0.11)	1.11 (0.19 to 2.03)	0.70 (0.26 to 1.13)	-0.05 (-0.20 to 0.10)	0.19 (-2.98 to 3.36)
7	CVD:	Yes	-0.35 (-0.55 to-0.15)	0.05 (-0.71 to 0.80)	0.10 (-0.52 to 0.72)	-0.02 (-0.08 to 0.05)	0.32 (-1.71 to 2.36)
8	Cancer:	Yes	-1.39 (-1.79 to-0.99)	-1.09 (-2.82 to 0.64)	0.76 (0.43 to 1.08)	-0.15 (-0.23 to-0.07)	-4.47 (-6.74 to -2.20)
9	COPD:	Yes	-0.70 (-1.80 to 0.39)	-0.10 (-1.93 to 1.74)	0.15 (-0.23 to 0.53)	-0.11 (-0.25 to 0.03)	-1.08 (-5.29 to 3.13)
10	Diabetes:	Yes	-1.43 (-1.65 to-1.22)	-2.39 (-3.26 to-1.52)	0.54 (-0.16 to 1.25)	-0.18 (-0.21 to-0.16)	-1.89 (-3.74 to -0.04)
11	Cholesterol /HDL^a^		-0.80 (-1.11 to -0.49)	-2.79 (-3.50 to-2.08)	-0.04 (-0.32 to 0.25)	-0.05 (-0.10 to-0.01)	-1.32 (-2.87 to 0.24)
12	LDL^a^		0.43 (0.23 to 0.62)	0.82 (0.17 to 1.46)	0.17 (-0.11 to 0.45)	0.05 (0.02 to 0.09)	0.69 (-0.50 to 1.89)
13	HDL^a^		1.19 (0.82 to 1.56)	3.53 (2.67 to 4.39)	-0.20 (-0.52 to 0.12)	0.11 (0.06 to 0.16)	1.21 (-0.83 to 3.24)
14	Creatinine^a^		0.19 (-0.07 to 0.46)	-2.15 (-2.76 to-1.54)	0.10 (-0.48 to 0.67)	0.03 (-0.01 to 0.07)	2.56 (1.19 to 3.94)
15	Lipid medications:	Yes	-0.77 (-0.91 to -0.63)	-2.17 (-3.23 to-1.10)	0.02 (-0.56 to 0.59)	-0.08 (-0.12 to-0.04)	-1.92 (-2.75 to-1.08)
16	Psychotropic drug use:	Yes	-0.54 (-0.99 to -0.08)	0.10 (-0.90 to 1.09)	-0.37 (-1.03 to 0.29)	-0.04 (-0.14 to 0.05)	-4.01 (-7.28 to -0.74)

a Effect sizes presented as change for 1 standard deviation increase around the Ref. value; Ref. values and standard deviations are listed in Supplementary Tables S3 (summary of harmonised variables) and S6 (reference values and standard deviations for continuous predictors).

b For categorical predictors effect size is the mean difference compared to men who were not married or in a de facto relationship, did not have higher education, consumed <19.2g/d of alcohol, did more physical activity, had Good/Excellent general health, or did not have the medical condition or use the medication listed, respectively.

c Values in parentheses are 95% confidence intervals of the summary estimates.

d Duration of vigorous-intensity physical activity ≤75 mins per week (versus > 75 mins per week).

e <Good = Fair, Poor or Very Poor (versus ≥Good = Good or Excellent).

SHBG=sex hormone-binding globulin, LH=luteinising hormone, DHT=dihydrotestosterone, BMI=body mass index, BP=blood pressure, CVD=cardiovascular disease, COPD=chronic obstructive pulmonary disease, HDL=high density lipoprotein, LDL=low density lipoprotein.
