# Supplementary material for: Factors associated with circulating sex hormones in men: Individual Participant Data meta-analyses
Source: Ann Intern Med. Author manuscript; Available in PMC 2024 Apr 5. (PMC10995451; doi:10.7326/M23-0342)
Supplement: Appendix [file EMS202204-supplement-Appendix.pdf]

Appendix

Factors associated with circulating sex hormones in men: Individual Participant Data meta-analyses.

Authors: Ross J Marriott, Kevin Murray, Robert J Adams, Leen Antonio, Christie M Ballantyne, Douglas C Bauer, Shalender Bhasin, Mary L Biggs, Peggy M Cawthon, David J Couper, Adrian S Dobs, Leon Flicker, David J Handelsman, Graeme J Hankey, Anke Hannemann, Robin Haring, Benjumin Hsu, Magnus Karlsson, Sean A Martin, Alvin M Matsumoto, Dan Mellström, Claes Ohlsson, Terence W O'Neill, Eric S Orwoll, Matteo Quartagno, Molly M Shores, Antje Steveling, Åsa Tivesten, Thomas G Travison, Dirk Vanderschueren, Gary A Wittert, Frederick C W Wu, Bu B Yeap.

Methods

Timing of blood sampling across cohorts

We requested an indicator variable identifying time of blood sample collection but this was provided in only 3 of the 9 IPD-level datasets received, so statistical adjustment for time of day was not possible. Accordingly, variable time of blood sampling among the studies might have contributed to observed heterogeneity. However, blood sampling was conducted in early morning for all participants in 5 of the 11 studies, and in the morning for all participants in 8 of the 11 studies, and all participants were fasting in 7 of the 11 studies (Supplementary Table S4A). Therefore, most of the blood sampling was undertaken in the morning, fasting.

Statistical analysis

Continuous predictors were modelled using restricted cubic splines and used values for centring and knot points that were calculated from the entire distribution of IPD and were consistent across studies (Supplementary Table S5). Within datasets, some of the harmonised hormone and predictor variables were completely missing, and percentages of incomplete cases were 0.05-47.3% (Supplementary Tables S2, S6). The percentage of incomplete cases in merged IPD varied with analysis model and ranged from 2.5% (Model 1) to 18.9% (Model 9; Supplementary Table S6). Missing values in the independent variables (IVs) were imputed using Substantive Model Compatible Fully Conditional Specification (SMCFCS). A congenial set of 40 imputations was done separately for each IPDMA.[1] In sensitivity analyses, SMCFCS imputations were repeated to impute the predictor of interest when completely missing from any dataset. Results from fitting each model to each imputed dataset in Stage 1 were pooled using Rubin's rules, prior to combining in Stage 2 (Supplementary Methods).

Multivariate meta-analysis for estimation of non-linear summary curves was used to combine study-specific estimates of spline model coefficients and covariance matrices in Stage 2.[2] This was done using the ‘mixmeta’ and ‘dlnm’ packages in R. The mixmeta package is an extension of the “mvmeta” package, in that it provides more general and updated options for these types of analyses, although mvmeta was required to calculate prediction intervals (Supplementary Methods).[3] The metagen and forest functions of the package ‘meta’ were used for summarising associations with categorical predictors.[2-4]

Results

Associations with sociodemographic factors (Model 1)

Estimates of I^2 showed appreciable relative heterogeneity for associations of testosterone with BMI, SHBG with age and BMI, and estradiol and DHT with age, with relatively wide CIs indicating high uncertainty in other estimates (Appendix Table A2). However, the shape of associations of testosterone with BMI was generally consistent among studies, with estimated change per SD increase in BMI around 27.5 kg/m^2 ranging from -3.18 (CI=-3.78,-2.59) to -2.01 nmol/L (CI=-2.37,-1.66) (Supplementary Fig. S3). Prediction intervals showed that the true effect for a new study would likely be consistent with these results, with possible exceptions for the associations of testosterone and SHBG with married/de facto status and of SHBG with higher education (Figure 1).

Associations with lifestyle factors (Model 2)

Relative heterogeneity was appreciable for the MD of DHT between Current- and Never-smokers, in other cases 95% CIs of I^2 were relatively wide indicating high uncertainty in estimates (Appendix Table A2; Supplementary Fig. S5d).

Associations with prevalent health and medical conditions (Models 3-16)

There was low relative heterogeneity for associations of testosterone with systolic BP, CVD, and diabetes, SHBG with psychotropic drug use, DHT with systolic and diastolic BP, and estradiol with COPD, LDL and creatinine. There was appreciable relative heterogeneity for associations of LH with creatinine and DHT with general health, and broad CIs in other I^2 estimates (Appendix Table A2; Supplementary Fig. S6g,S10). Prediction intervals showed the true effect for a new study would likely be consistent for associations with diabetes, CVD, cancer, lipid-lowering medication and psychotropic drug use, with broader uncertainty in predicting the true association of testosterone with other health conditions, reflecting in part the variation within studies, estimated heterogeneity between studies, and the number of IPD-level datasets analysed (Figure 2).

References cited within Appendix

1. Rubin DB. Multiple imputation for nonresponse in surveys. New York: Wiley, 1987.
2. Gasparrini A, Armstrong B, Kenward MG, Multivariate meta-analysis for non-linear and other multi-parameter associations. Statist Med 2012; 31: 3821–39.
3. Sera F, Armstrong B, Blangiardo M, Gasparrini A. An extended mixed-effects framework for meta-analysis. Statist Med 2019; 38: 5429–44.
4. Balduzzi S, Rücker G, Schwarzer G. How to perform a meta-analysis with R: a practical tutorial. Evid Based Ment Health 2019; 22: 153–160.

Funding sources

This work was funded by a Rapid Applied Translation Grant from the Australian Government's Medical Research Future Fund, a Future Health Research and Innovation Focus grant from the Government of Western Australia's Department of Health, and via

philanthropic donations by Lawley Pharmaceuticals to the University of Western Australia. Funding statements for each individual study involved in this meta-analysis are provided in the Supplementary Materials. The funders had no role in the collection, analysis, and interpretation of data; nor in the writing or submission of this article.

Acknowledgements

We thank Tinh-Hai Collet (Service of Endocrinology, Diabetes, Nutrition and Therapeutic Education, Department of Medicine, Geneva University Hospitals), Marcus Dörr (Department of Internal Medicine B Cardiology, Angiology & Pneumology, University Medicine Greifswald), Östen Ljunggren (Department of Medical Sciences, Uppsala University) and Liesbeth Vandenput (Centre for Bone and Arthritis Research, Sahlgrenska Academy Institute of Medicine, University of Gothenburg). We thank John Acres, Mark Divitini, Tiffany Gill, Stephanie Harrison, Kristin Henselin, Matthew Knuiman, Karen Mutalik, Maria Nethander, Lisa Reeves, Jos Tournoy, the staff and participants of the ARIC Study, the Busselton Health Study, the Cardiovascular Health Study, the CHAMP Study, the EMAS Study, the Framingham Heart Study, the Health In Men Study, the MAILES Study, the MrOS USA Study (https://mrosonline.ucsf.edu), MrOS Sweden Study and the SHIP Study for their important contributions, and the Busselton Population Medical Research Foundation for access to data. This manuscript has been reviewed by the ARIC Study and CHS, for scientific content and consistency of data interpretation with previous ARIC Study and CHS publications.

Data sharing statement

The study protocol and systematic review are both published in an open access journal.[24,25] Computer code used to generate the results reported is available upon reasonable request. IPD

and AD are confidential but applications for data access can be made to individual studies (refer to Supplementary Material regarding generation of AD statistics from the respective cohorts). Availability of data to share are governed by the ethical approvals of the original cohort studies contributing to the meta-analyses.

Table A1. Summary attributes: cross-sectional androgen data at baseline by study.[a] Atherosclerosis Risk in Communities Study *ARIC*,[27] Busselton Health Study *BHS*,[28] Cardiovascular Health Study *CHS*,[29,30] European Male Ageing Study *EMAS*,[31] Framingham Heart Study *FHS*,[32,33] Health In Men Study *HIMS*,[34] Men Androgen Inflammation Lifestyle Environment and Stress study *MAILES*,[35] *MrOS* Osteoporotic Fractures in Men USA study,[36-38] Study of Health in Pomerania *SHIP*,[39] Concord Health and Ageing in Men Project *CHAMP*,[40] *MrOS* Osteoporotic Fractures in Men Study in Sweden[41]

Study	Data	Country	Baseline[b]	n[c]	Age[c] (years)	BMI[c] (kg/m^2)	Testosterone (nmol/L)	SHBG (nmol/L)	LH (IU/L)
ARIC	IPD	USA	1996 - 1998[d]	1,556	63.0 (58.0-68.0)	27.7 (25.4-30.7)	13.1 (10.0-16.6)	31.5 (23.2-41.7)	Not available
BHS	IPD	Australia	1994 - 1995[e]	2,021	49.9 (37.6-64.8)	26.2 (24.1-28.6)	13.0 (10.1-16.6)	27.3 (20.4-35.7)	3.4 (2.5-4.6)
CHS	IPD	USA	1994 - 1995[f]	1,123	76.0 (73.3-79.9)	26.4 (24.2-28.8)	12.7 (9.5-16.5)	58.5 (44.7-79.0)	Not available
EMAS	IPD	European	2003 - 2005[g]	2,832	59.2 (50.1-69.2)	27.4 (24.9-30.0)	16.2 (12.6-20.4)	39.5 (29.1-52.3)	5.3 (3.8-7.2)
FHS	IPD	USA	2002 - 2006[h]	3,334	49.0 (39.0-59.0)	27.7 (25.2-30.5)	20.4 (15.7-26.2)	43.6 (30.6-60.4)	Not available
HIMS	IPD	Australia	2001 - 2004[i]	4,121	76.0 (74.0-79.0)	26.3 (24.2-28.6)	12.4 (9.5-15.6)	39.6 (31.4-50.4)	4.4 (3.0-6.8)
MAILES	IPD	Australia	2002 - 2006[j]	1,975	55.0 (46.0-64.0)	28.0 (25.4-30.7)	16.5 (12.8-20.6)	31.0 (23.8-41.0)	4.7 (3.0-6.3)
MrOS USA	IPD	USA	2000 - 2002[k]	2,002	73.0 (68.0-77.0)	27.0 (24.8-29.4)	13.4 (10.3-17.0)	45.9 (35.3-58.7)	4.9 (3.5-7.5)
SHIP	IPD	Germany	1997 - 2010[l]	2,110	51.0 (37.0-64.0)	27.4 (25.0-30.0)	15.4 (11.9-19.6)	44.6 (33.0-58.8)	Not available
CHAMP	AD	Australia	2005 - 2007[m]	1,659	76.0 (72.0-80.0)	27.6 (25.1-30.2)	14.4 (11.1-18.3)	46.6 (35.3-61.1)	7.3 (5.1-11.1)
MrOS Sw.	AD	Sweden	2001 - 2004[n]	2,416	75.4 (72.9-78.5)	26.1 (23.9-28.4)	15.2 (11.8-19.1)	39.5 (29.3-53.2)	Not available

a. Medians and 25th and 75th percentiles presented for continuous variables.
b. The period when blood samples were taken for subsequent testosterone mass spectrometry assay, which defines the start of follow-up for future AIMS analyses of prospective health outcomes.[24] Except for cardiovascular outcomes of MrOS USA (from Dec 2003 onwards).
c. n = total sample size for adult male participants after exclusions and for whom mass spectrometry measurements of testosterone were available. Age and body mass index (BMI) are presented for these n participants.
d. Visit 4 of the ARIC Study.[27]
e. 1994/95 survey of the BHS.[28]
f. Visit 7 of the CHS. Excludes men being treated for cancer at enrolment (1989 or 1992), and those with history of cardiovascular disease at baseline.[29,30]
g. EMAS participants were from Belgium, Estonia, Hungary, Italy, Poland, Spain, and the United Kingdom (Manchester).[31]
h. Exam 7 of Offspring cohort and Exam 1 of Gen3 cohorts combined.[32,33]
i. HIMS[34]

j. Wave 1 of the FAMAS and Wave 2 of the NWAHS combined. Excludes men with dementia at baseline.[35]
k. Includes data for the sub-cohorts only and excludes participants who were later included as cases, as part of this study's case-cohort design.[36-38]
l. SHIP-0 (1997-2001) and SHIP-Trend (2008-12) cohorts combined.[39]
m. CHAMP[40]
n. MrOS Sweden[41]

BMI=body mass index, SHBG=sex hormone-binding globulin, LH=luteinising hormone, IPD=individual participant data, AD=aggregate data.

Table A2. Analysis design and statistical hypotheses.

Model(s)	Mathematical Representation[d]	Hypothesis(es)	Context
1[a]	T = f(sociodemographic variables)	Sociodemographic variable *k* has an independent association with the level of endogenous *T*.	That is, after controlling for other *K*-1 socio-demographic variables in the model, where *k* = 1, …, *K*.
2[b]	T = f(Model 1 terms + lifestyle variables)	Lifestyle variable *l* has an independent association with the level of endogenous *T*	That is, after controlling for other *L*-1 lifestyle and *K* sociodemographic variables in the model, where *l* = 1, …, *L*.
3-16[c]	T = f(Model 2 terms + prevalent condition_j)	Health condition *j* has an independent association with the level of endogenous *T*	That is, after controlling for *L* lifestyle and *K* sociodemographic variables in the model.

a. Sociodemographic variables: Age, body mass index (BMI), Marital status, Education.
b. Lifestyle variables: Alcohol consumption, Smoking status, Physical Activity
c. Health condition predictors used in each of Models 3-16, respectively, were: Diastolic blood pressure, systolic blood pressure, hypertension, health status, cardiovascular disease (CVD), cancer, chronic obstructive pulmonary disease (COPD), diabetes, Total cholesterol / HDL, low density lipoprotein (LDL), high density lipoprotein (HDL), creatinine, lipid-lowering medication use, psychotropic drug use.
d. T = total testosterone (dependent variable). Analyses repeated by replacing T for sex hormone-binding globulin (SHBG), luteinising hormone (LH), dihydrotestosterone (DHT), estradiol as the dependent variable, for those studies that had provided measurements.

Table A3. Relative heterogeneity for testosterone, SHBG, LH, DHT and estradiol associations (%).

		Heterogeneity (I^2; %)[a]				
	Dependent var:	Testosterone	SHBG	LH	DHT	Estradiol
Model	Predictor					
Social/demographic predictors						
1	Age	67.1 (45.4 to 80.2)	95.5 (94.1 to 96.7)	23.1 (0.0 to 63.6)	81.1 (65.1 to 89.8)	85.6 (77.0 to 91.0)
1	BMI	67.7 (53.6 to 77.5)	74.2 (63.8 to 81.6)	22.0 (0.0 to 56.4)	50.9 (14.4 to 71.9)	40.3 (3.7 to 63.0)
1	Married or de facto	45.7 (0.0 to 74.8)	24.7 (0.0 to 64.6)	0.0 (0.0 to 65.4)	53.1 (0.0 to 82.7)	71.8 (38.8 to 87.0)
1	Higher education	7.1 (0.0 to 70.2)	38.9 (0.0 to 73.0)	0.0 (0.0 to 50.4)	0.0 (0.0 to 25.4)	40.1 (0.0 to 76.3)
+ Lifestyle predictors						
2	Alcohol consumed	63.9 (26.1 to 82.4)	53.8 (1.9 to 78.2)	70.0 (23.6 to 88.3)	0.0 (0.0 to 78.3)	20.7 (0.0 to 64.1)
2	Physical activity[b]	60.4 (17.8 to 80.9)	0.0 (0.0 to 60.1)	57.9 (0.0 to 84.3)	19.3 (0.0 to 84.2)	3.3 (0.0 to 72.2)
	Smoking:					
2	Former v Never	15.9 (0.0 to 58.0)	0.0 (0.0 to 54.5)	30.3 (0.0 to 73.2)	0.0 (0.0 to 78.3)	54.8 (0.0 to 80.6)
2	Current v Never	62.0 (21.5 to 81.6)	69.2 (37.5 to 84.6)	0.0 (0.0 to 43.5)	87.5 (73.3 to 94.2)	6.9 (0.0 to 73.3)
+ Prevalent health						
3	Diastolic BP	64.4 (47.1 to 76.0)	45.0 (14.7 to 64.5)	65.3 (37.4 to 80.7)	9.6 (0.0 to 46.2)	44.8 (7.7 to 67.0)
4	Systolic BP	20.5 (0.0 to 48.5)	26.2 (0.0 to 52.1)	64.2 (40.0 to 78.7)	0.0 (0.0 to 49.7)	42.1 (6.8 to 64.0)
5	Hypertension	53.6 (1.5 to 78.1)	59.6 (15.9 to 80.6)	0.0 (0.0 to 79.3)	46.5 (0.0 to 80.4)	12.8 (0.0 to 75.0)
6	General health	60.1 (8.5 to 82.6)	0.0 (0.0 to 70.0)	20.9 (0.0 to 89.8)	81.4 (51.5 to 92.9)	61.3 (5.6 to 84.2)
7	CVD	0.0 (0.0 to 41.1)	5.2 (0.0 to 69.6)	73.3 (33.3 to 89.3)	22.7 (0.0 to 90.0)	36.0 (0.0 to 73.0)
8	Cancer	35.0 (0.0 to 70.1)	61.0 (19.2 to 81.2)	0.0 (0.0 to 66.9)	36.2 (0.0 to 76.2)	0.0 (0.0 to 71.2)
9	COPD	70.9 (32.4 to 87.5)	44.3 (0.0 to 78.0)	0.0 (0.0 to 75.8)	43.8 (0.0 to 81.2)	5.5 (0.0 to 18.7)
10	Diabetes	0.0 (0.0 to 38.7)	23.1 (0.0 to 63.6)	73.8 (34.7 to 89.5)	0.0 (0.0 to 79.2)	0.0 (0.0 to 65.1)
11	Cholesterol /HDL	41.5 (11.2 to 61.4)	51.9 (28.3 to 67.7)	37.3 (28.3 to 67.7)	36.5 (0.0 to 64.5)	45.3 (12.4 to 65.8)
12	LDL	36.4 (2.8 to 58.3)	55.2 (33.7 to 69.7)	58.6 (33.7 to 69.7)	0.0 (0.0 to 50.2)	0.0 (0.0 to 33.8)
13	HDL	47.4 (21.0 to 65.0)	59.8 (41.1 to 72.5)	72.3 (41.1 to 72.5)	14.9 (0.0 to 51.4)	37.8 (0.0 to 61.6)
14	Creatinine	49.3 (24.1 to 66.1)	56.6 (36.0 to 70.6)	72.3 (55.0 to 83.0)	65.0 (41.4 to 79.1)	2.7 (0.0 to 45.4)
15	Lipid medications	0.0 (0.0 to 64.8)	43.2 (0.0 to 73.8)	75.9 (40.9 to 90.1)	0.0 (0.0 to 73.5)	0.0 (0.0 to 70.8)

16	Psychotropic drug use	0.0 (0.0 to 68.8)	0.0 (0.0 to 45.1)	0.0 (0.0 to 94.6)	0.0 (0.0 to 86.7)	0.0 (0.0 to 76.4)

[a] Values in parentheses are 95% confidence intervals of the estimates. Calculated using formulas in Borenstein et al (Supplementary Material).

[b] Duration of vigorous-intensity physical activity ≤ 75 mins per week.

SHBG=sex hormone-binding globulin, LH=luteinising hormone, DHT=dihydrotestosterone, BMI=body mass index, BP=blood pressure, CVD=cardiovascular disease, COPD=chronic obstructive pulmonary disease, HDL=high density lipoprotein, LDL=low density lipoprotein.

Table A4. Sensitivity of summary estimates of effect size for total testosterone (nmol/L) to adjustments from additional model terms. For reference, estimates that were presented in the main set of analysis results are in **bold**.

Predictor	Model 1 *(Social/demographic)*	Model 2 *(Model 1 + Lifestyle)*	Model 7 *Model 2 + CVD*	Model 10 *Model 2 + Diabetes*
Social/demographic predictors				
Age	**-1.24 (-1.61,-0.87)**	-1.11 (-1.47,-0.75)	-0.98 (-1.37,-0.59)	-1.04 (-1.40,-0.68)
BMI	**-2.42 (-2.70,-2.13)**	-2.37 (-2.66,-2.09)	-2.38 (-2.69,-2.07)	-2.31 (-2.60,-2.02)
Married or de facto	**-0.57 (-0.89,-0.26)**	-0.50 (-0.78,-0.21)	-0.50 (-0.82,-0.17)	-0.53 (-0.81,-0.24)
Higher education	**-0.10 (-0.33, 0.13)**	-0.09 (-0.30, 0.12)	-0.13 (-0.34, 0.08)	-0.10 (-0.30, 0.10)
Lifestyle predictors				
Alcohol consumed		**-0.17 (-0.55, 0.20)**	-0.15 (-0.55, 0.25)	-0.20 (-0.57, 0.17)
Physical activity[b]		**-0.51 (-0.90,-0.13)**	-0.46 (-0.83,-0.09)	-0.49 (-0.86,-0.12)
Smoking:				
Former v Never		**-0.34 (-0.55,-0.12)**	-0.38 (-0.57,-0.19)	-0.30 (-0.51,-0.10)
Current v Never		**0.89 (0.36, 1.42)**	1.00 (0.54, 1.47)	0.92 (0.40, 1.44)
Prevalent health				
CVD			**-0.35 (-0.55,-0.15)**	
Diabetes				**-1.43 (-1.65,-1.22)**

[a] Values in parentheses are 95% confidence intervals of the estimates. Calculated using formulas in Borenstein et al (Supplementary Material).
[b] Duration of vigorous-intensity physical activity ≤ 75 mins per week.
CVD=cardiovascular disease, BMI=body mass index.
