# Supplementary material for: Factors associated with circulating sex hormones in men: Individual Participant Data meta-analyses
Source: Ann Intern Med. Author manuscript; Available in PMC 2024 Apr 5. (PMC10995451; doi:10.7326/M23-0342)
Supplement: Supplementary material [file EMS202204-supplement-Supplementary_material.pdf]

Supplementary Materials

Factors associated with circulating sex hormones in men: Individual Participant Data meta-analyses.

Authors: Ross J Marriott, Kevin Murray, Robert J Adams, Leen Antonio, Christie M Ballantyne, Douglas C Bauer, Shalender Bhasin, Mary L Biggs, Peggy M Cawthon, David J Couper, Adrian S Dobs, Leon Flicker, David J Handelsman, Graeme J Hankey, Anke Hannemann, Robin Haring, Benjumin Hsu, Magnus Karlsson, Sean A Martin, Alvin M Matsumoto, Dan Mellström, Claes Ohlsson, Terence W O'Neill, Eric S Orwoll, Matteo Quartagno, Molly M Shores, Antje Steveling, Åsa Tivesten, Thomas G Travison, Dirk Vanderschueren, Gary A Wittert, Frederick C W Wu, Bu B Yeap.

Table of Contents

Supplementary Methods 3
- IPD integrity and risk of bias within studies 4
- Multiple imputations 4
- Modelling of categorical predictors and forest plots 6
- Non-linear modelling of continuous predictors in IPDMAs 6
- Heterogeneity 9
- Sensitivity Analysis 1: Imputing the predictor of interest 9
- Sensitivity Analysis 2: Modelling age using a restricted cubic spline 11
- Sensitivity Analysis 3: Ethnicity 12
- Sensitivity Analysis 4: Adjustments from the inclusion of additional model terms. 13
- Sensitivity Analysis 5: Including aggregate data from additional studies 13
- Funnel plots for assessing publication bias 14

Supplementary Results 14
- IPD integrity and risk of bias within studies 14
- Heterogeneity 18
- Sensitivity Analysis 1: Imputing the predictor of interest 20
- Sensitivity Analysis 2: Modelling age using a restricted cubic spline 21
- Sensitivity Analysis 3: Ethnicity 21
- Sensitivity Analysis 4: Adjustments from the inclusion of additional model terms. 23
- Sensitivity Analysis 5: Including aggregate data from additional studies 23
- Funnel plots for assessing publication bias 24
- Exploratory subgroup analysis of men without common age-associated medical comorbidities 25

Funding statements 27

Supplementary Tables 29
- Table S1. PRISMA-IPD Checklist 29
- Table S2. Harmonised variables summary 35
- Table S3. Definitions of harmonized variables 37
- Table S4A. Recruitment criteria for participants 41
- Table S4B. Recruitment criteria for participants 44
- Table S5. Values used for spline modelling of continuous predictors 45
- Table S6. Missing data summary 46

Supplementary Figures 52

References cited 75

Supplementary Methods

This first Androgens In Men Study (AIMS) meta-analysis is exploratory, in that it summarises available evidence with the aim of providing more general descriptions of factors influencing hormone concentrations in men. Accordingly, we focus on describing the nature of estimated trends, size of estimated effects, and heterogeneity using pre-specified statistical models, with all results presented for completeness, rather than a focus on testing specific hypotheses.

Search strategy and bridge search

A systematic review was conducted between 14 June to 31 December 2019. The literature search used 4 online search tools, with no date restrictions. This covered the MEDLINE and EMBASE databases, and the OpenGrey and Mednar grey literature search tools from inception up to 18-22 July 2019. The final selection of articles was completed on 31 December 2019. There were 2,177 articles (1,738 published and 439 grey literature) identified. Two reviewers independently screened the de-duplicated articles against pre-specified criteria. 20 articles were identified as within scope, plus 5 that were possibly in scope, requiring further investigation. From this, 11 prospective cohort studies were selected as being suitable to approach for IPD-level data. This process of systematic review has been peer-reviewed and published.[1]

To screen for additional cohorts that may have published relevant data since the original search, a bridge search was undertaken on 2 May 2023. This covered the MEDLINE database using the original search strategy, date limited from 15 July 2019 to 2 May 2023. There were 333 articles identified. One reviewer screened all articles against the original pre-specified criteria. On completion of screening based on title and abstracts, 28 articles progressed to

further evaluation. Nine of these articles described studies of interest. Five articles identified potentially eligible cohort studies, all previously identified in the original search (EMAS 3, MAILES 1, ARIC 1). Two articles cited one cohort study, not identified in the original search, which had since become potentially eligible (NHANES 2011-14). An additional two articles cited a cohort study first reported after completion of the original search (Henan Rural Cohort Study).

IPD integrity and risk of bias within studies

Newcastle-Ottawa Quality Assessments reported the number of stars against each scoring category to assess overall risk of bias within each study.[1, 2] Once individual participant data (IPD) were received, cross-tabulations and summary statistics were provided back to data managers of respective studies for checking and feedback. The means and standard deviations of testosterone were calculated using IPD with similar exclusions to those that were used in publications and compared with the published estimates. IPD were also checked for conspicuously low or high values using scatterplot matrices, box and whisker plots, and by fitting preliminary models to complete-case IPD for assessing plots of residual diagnostics and DFBETAs. Potential outliers identified from this process were queried with the respective data managers. Various aspects of IPD integrity were identified and discussed (Supplementary Results).

Multiple imputations

IPD were multiply imputed using Substantive Model Compatible Fully Conditional Specification (SMCFCS), with R software.[3] This method assumes that missing observations are missing at random, conditional on observed variables, and that the method of imputation is suitably consistent or “congenial” with each substantive model of interest.[4, 5] Each set of 40

imputations was produced using an imputation model that was assumed to be congenial with each specific IPD Meta-Analysis (IPDMA) model. The "smformula" argument of the "smcfcs" function allowed specification of each imputation model to include: (i) the androgen (e.g., testosterone) as the dependent variable; (ii) continuous predictors modelled using cubic splines with the same knot points as specified for the respective IPDMA model; and (iii) categorical covariates with consistent coding as used in the IPDMA model. In the presented analyses the authors wanted to acknowledge that the true effect size in each of the cohort studies may vary due to differences in local factors, and so random effects IPDMAs were used. Therefore, a congenial SMCFCS imputation model was approximated by also including: (iv) interaction terms between study and each of the other model terms. Multi-level imputation models, including JM-jomo and FCS-2 stage[6-8] were also investigated, but these methods were aborted due to problems with imputations of the systematically missing variables; likely because of the relatively small number of studies (i.e., 3-9) with IPD requiring imputation. Although this implementation of SMCFCS was not a multi-level method, it was considered optimal because of efficiencies in imputation model specification and computation time. For each imputation 20 iterations were done and the rejection limit was increased above the default setting if warnings identified that imputed values were rejected.

Occasionally, the SMCFCS imputation model was reduced for the purpose of resolving convergence problems. A cubic polynomial was specified for age because convergence could not be achieved when imputing a cubic spline for age. For analyses of estradiol, the model term for COPD status was inestimable because it had zero events observed in MAILES data. Therefore, imputations and the IPDMA was done for only those studies that supplied IPD (after exclusions) that included estradiol measurements and participants with a history of

COPD: ARIC, BHS, CHS, HIMS, MrOS USA. IPD from the CHS were omitted from IPDMAs of the association with prevalent CVD because participants with history of CVD were excluded in those data.[9] Creatinine was log-transformed prior to imputations to account for pronounced skew. Prior to log-transformation a small constant of 0.02 was added to account for a measurement of 0 μmol/L, because adding 0.02 did not change the log-transformed values of the smallest 25 non-zero values, when rounded to two decimal places.

Modelling of categorical predictors and forest plots

This is a two-stage IPD meta-analysis. Reference values for categorical predictors are listed in Supplementary Table S2. The study-specific estimates shown in forest plots were obtained from Stage 1 modelling, where the same multivariable model was fitted to all datasets from each study separately. These estimated coefficients and their standard errors were used as inputs in Stage 2 modelling using REML estimation and the metagen function, from the R package 'meta'. Outputs from this model included the summary estimate, the 95% confidence intervals of the summary estimate, and the prediction interval.

Non-linear modelling of continuous predictors in IPDMAs

Continuous variables were centred by the same values across studies and modelled using restricted cubic splines. For spline modelling, the same knot values were used in all studies, to ensure that estimates could be combined in Stage 2 in a coherent way.[10] Restricted cubic splines were fitted with knot values at the 5th, 27.5th, 50th, 72.5th, and 95th percentiles, which are recommended knot placements from Harrell,[11] as determined from the entire IPD from all studies[10]:

Variable	Range common to all studies	Centering value	Knot points
**Age**	70-74	70	40, 70, 78
**BMI**	17.7-40.4	27.5	21.6, 25.0, 27.1, 29.4, 34.8
**Systolic BP**	92-200	135	108.0, 123.0, 134.5, 148.0, 174.5
**Diastolic BP***	54-108	80	60.0, 72.0, 79.0, 85.0, 99.5
**Creatinine**	61.9-247.8	90	62.0, 78.0, 88.0, 97.3, 123.9
**HDL**	0.5-2.7	1.3	0.8, 1.0, 1.2, 1.4, 1.9
**LDL**	1.0-6.1	3.2	1.8, 2.6, 3.1, 3.7, 4.8
**Total cholesterol : HDL ratio**	1.9-9.3	4.3	2.5, 3.4, 4.1, 4.9, 6.8

Age was problematic for spline modelling because of varying distributions of participant ages among studies:

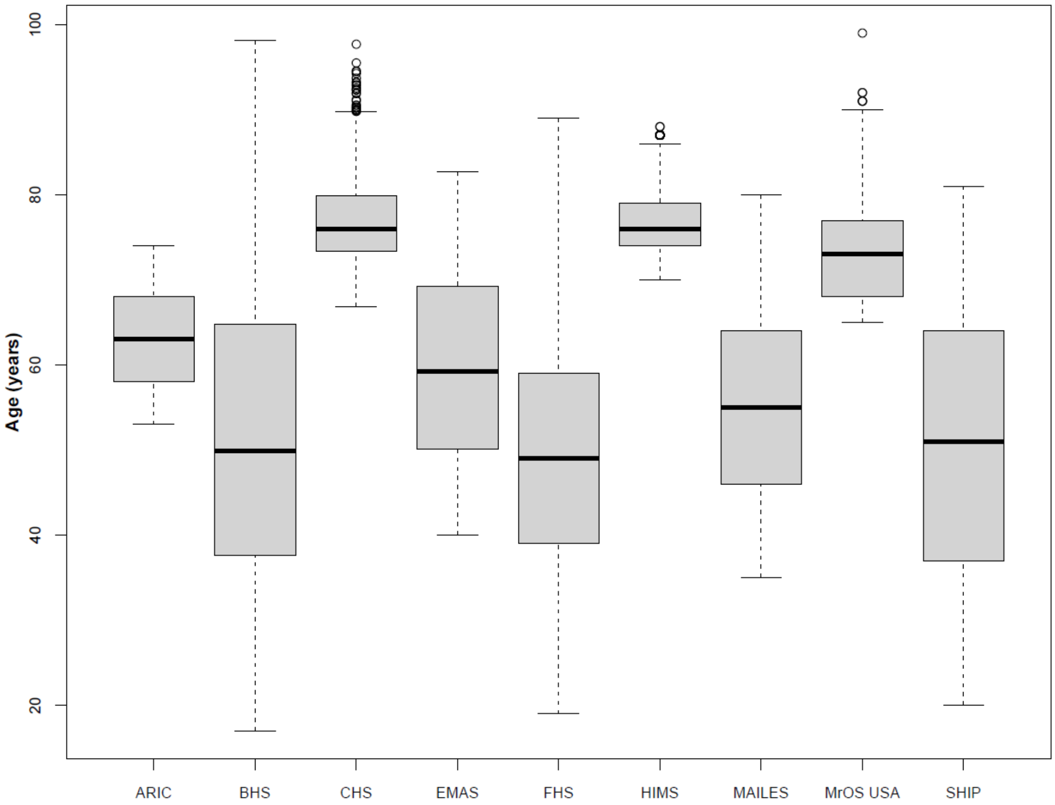

The age range across all studies combined was 17-99 years but the overlapping age range among studies was just 70-74 years, which presented a challenge for the selection of knot values. To account for different distributions of age, we used an approach similar to that of Riley et al.[10] A restricted cubic spline was fitted to age, with an internal knot set at 70 yr (60th

percentile) and outer knots at 40 yr (11.5th percentile) and 78 yr (88.5th percentile). The first and second knots were placed at values where the data ended in some studies (EMAS and HIMS, respectively), and the second knot was within the observed age range across all studies. Age was centred at 70 yr, to ensure that its spline term was centred within observed IPD for model fitting to each dataset in Stage 1 of the IPDMAs.

Multivariate meta-analysis for estimation of non-linear summary curves was used to combine study-specific estimates of spline model coefficients and covariance matrices in Stage 2.[12] This was done using the 'mixmeta' and 'dlnm' packages in R. The mixmeta package is an extension of the previously developed package "mvmeta", in that it provides more general and updated options for these types of analyses.[13] However, prediction intervals were obtained by applying the predict function—with the interval argument set to 'prediction'—to the R object returned from fitting the model using mvmeta, because this had not been implemented in mixmeta. The mvmeta-estimated covariance matrix $\boldsymbol{\Sigma}$ of the pooled spline coefficients was then used as the input for the 'vcov' argument of the 'crosspred' function (from the dlnm package). The $\boldsymbol{\Sigma}$ matrix is the sum of independent within-study and between-study covariance matrices.[12] The resulting predicted values (summary curve), their standard errors, and the 97.5th percentile of the *t* distribution, with *k*-2 degrees of freedom—where *k* is the number of studies—were then used to calculate 95% prediction intervals for each summary curve. The metagen function of the package 'meta' was used to obtain summary estimates for associations with categorical predictors and the forest function was applied to the returned R object, to generate forest plots. [12-14]

Heterogeneity

The relative extent of heterogeneity was quantified using I^2.[15] I^2 was used to represent heterogeneity because, unlike other measures, it does not depend on the number of studies or scale of measurement.[16] However, estimates may be biased for meta-analyses of a small number of studies (e.g., ≤ 7), and so, following von Hippel,[17] 95% confidence intervals (CIs) were presented. Ninety five percent CIs were calculated using the method in Borenstein et al.,[16] which uses the value of the Q statistic and its degrees of freedom. On two occasions this method failed to produce a realistic estimate (i.e., a CI of zero width), and in those cases the CIs were calculated using the method of Viechtbauer.[18]

The 95% CIs of I^2 were reported for all analyses and the range of effect sizes reported where appreciable relative heterogeneity was indicated. Higgins et al.[14] tentatively assigned an I^2 value of 50% as "moderate". Therefore, in this study it was considered that an appreciable level of relative heterogeneity was present when more than 50% of the observed variation could be attributed to variation in the true effects. This was indicated wherever the 95% CI for I^2 was above 50%: $[I^2_{\text{Lower}}, I^2_{\text{Upper}}] > 50\%$. We chose not to interpret point estimates of I^2 because of an increased prospect for bias when estimated for relatively small numbers of studies[16] and otherwise high uncertainty in the point estimate when the 95% CI was comparatively wide.

Sensitivity Analysis 1: Imputing the predictor of interest

The following predictors of interest were not available in all IPD-level datasets: history of anxiety, COPD, cardiovascular disease (CVD), and dementia, cognition score, diastolic blood pressure (BP), ethnicity, general health, higher education, ethnicity, psychotropic drug use,

and waist circumference (Table S2). CVD could not be systematically imputed for CHS IPD because history of CVD was an exclusion criterion for those data.[9] Waist circumference was not included in analyses because of its high correlation with BMI.[19] Ethnicity was not imputed because "White" was the predominant (if not the only) ethnicity type reported for almost all studies, so the sensitivity of results to omitting data for participants that were not of "White" ethnicity was explored instead (Sensitivity Analysis 3). Baseline cognition and depression were not imputed because they were measured too differently for harmonisation (refer "IPD integrity and risk of bias within studies" in Supplementary Results). Furthermore, prevalent dementia, baseline cognition, and anxiety were obtained from too few studies, and prevalent dementia was sparsely distributed (i.e., one case after exclusions for ARIC and FHS IPD).

Since measurements on the hormone variable were a selection criterion for study inclusion,[1, 20] the IPDMAs were done for only those studies that provided data on that hormone (dependent variable). For the main set of results, summary estimates were calculated using only those IPD-level datasets that included both the hormone and predictor of interest. In this sensitivity analysis we refer to this approach of omitting datasets without IPD on the predictor of interest or hormone as "Excluded". An alternative method is to impute the missing variable representing the predictor of interest, for datasets that included the hormone variable. In this sensitivity analysis we refer to this method as "Imputed". This analysis compares summary estimates and curves from IPDMAs calculated using the Excluded method with those calculated using the Imputed method, to evaluate the sensitivity to results from imputing the missing predictor of interest.

The SMCFCS method (described above) was used to impute the following missing predictors of interest: higher education for BHS (Model 1 analyses); diastolic BP for MrOS USA (Model 10 analyses); general health status for ARIC and BHS (Model 6 analyses); COPD status for EMAS, FHS, and SHIP (Model 9 analyses); and psychotropic drug use status for BHS and MrOS USA (Model 16 analyses).

Sensitivity Analysis 2: Modelling age using a restricted cubic spline

To account for modelling uncertainty due to knot placements for the restricted cubic spline being outside of observed data in some studies, the Model 1 IPDMAs were repeated by modelling age using a cubic polynomial, for comparison. A cubic polynomial was selected because, although it offered reduced flexibility of fit in comparison to a 3-knot restricted cubic spline, a similar number of non-intercept parameters (i.e., 3) could be estimated to describe non-linearity, without the need to specify knot values. Pooled multiply-imputed estimates obtained from the fit of each model to IPD from each study in Stage 1 were then combined in Stage 2 to obtain summary curves and 95% confidence envelopes. Summary curves with 95% confidence envelopes were overlayed on the same plots to visually compare estimates calculated using the different non-linear models for age.

Sensitivity Analysis 3: Ethnicity

An ethnicity variable was not provided by four of the studies although for SHIP it was reported that all participants were Caucasian. The other three studies (BHS, EMAS, HIMS) reported that the majority, if not all, participants were of white ethnicity. Gunnell et al.[21] reported that BHS participants were almost entirely of White ethnic background. It is estimated that HIMS is comprised of approximately 95% Caucasian participants, EMAS is comprised of approximately 95-99% Caucasian, and LeBlanc et al.[22] estimated that in MrOS USA data the percentage Caucasian ranged from 88.9-93.3%.

The sensitivity of results to the inclusion of ethnicity types other than "White" was investigated. This approach was used in preference to imputing the systematically missing ethnicity variable because in IPD where ethnicity type was known, non-White types were sparsely distributed, with the exception of ARIC which included approximately 25% Black participants. Firstly, for IPD that included ethnicity (ARIC, CHS, FHS, MAILES (FAMAS Wave 1 cohort), MrOS USA), summary estimates and summary curves for White ethnicity participants versus any ethnicity (White + not White) were compared. Secondly, results from the main analyses (all IPD) were compared with various subsets of IPD comprising White participants: (i) IPD identified as "White" from an ethnicity variable ("Ethnicity=White": ARIC, CHS, FHS, MAILES (FAMAS Wave 1 cohort), MrOS USA); (ii) subset (i) combined with IPD from one study comprised entirely of Caucasian participants ("White" + "All White"; ARIC, CHS, FHS, MAILES (FAMAS Wave 1 cohort), MrOS USA, SHIP); (iii) subset (ii) combined with IPD from two studies comprised almost entirely of White participants ("White" + "All White" + "Almost Entirely" White: ARIC, BHS, CHS, FHS, HIMS, MAILES (FAMAS Wave 1 cohort), MrOS USA, SHIP). These comparisons were

done for the associations of total testosterone with each of the Model 1 and Model 2 predictors, and with history of CVD (Model 7) and diabetes (Model 10).

Sensitivity Analysis 4: Adjustments from the inclusion of additional model terms.

Summary effects for the associations of total testosterone with sociodemographic (age, BMI, marital status, higher education) and lifestyle (alcohol consumption, physical activity, smoking status) predictors were estimated from more complex multivariable models to evaluate the sensitivity to further adjustments from the inclusion of additional model terms.

Sensitivity Analysis 5: Including aggregate data from additional studies

Aggregate statistics (AD: sample size, summary statistics, coefficient estimates, and variance estimates or covariance matrices, plot data) were supplied by two additional studies (CHAMP, MrOS Sweden). AD statistics were generated by data managers of each respective study using an R script provided. The R script contained the same syntax as used for Stage 1 of the respective IPDMAs. Once the data manager had read in the requisite IPD—which had not been supplied to AIMS—this R script generated and outputted the AD as a list object saved as an RDA file, plus one CSV file containing summary statistics for Table 1. These files were then sent to the AIMS Data Manager. AD coefficient estimates and variance estimates (or covariance matrices) were then combined with those generated from supplied IPD in Stage 2, to produce the summary estimates (categorical predictors) or summary curves (continuous predictors). These analyses were completed for associations of total testosterone with each of the socio-demographic (Model 1) and lifestyle (Model 2) predictors, and with history of CVD (Model 7) and diabetes (Model 10).

Summary estimates calculated by including AD were then compared with those from the

main set of results (i.e., using IPD only) in a forest plot to evaluate potential availability bias. For these comparisons, the summary effect of continuous predictors modelled using restricted cubic splines (age and BMI) were calculated from summary curves as the change arising from 1 SD increase around the Ref. value (Table S5).

Funnel plots for assessing publication bias

Contour-enhanced funnel plots were constructed to explore the prospect for publication bias in meta-analyses including AD. Egger's regression test was used for plots of at least 10 studies and because of the low power of these tests we used P<0.1 in tests for asymmetry.[23,24] Tests of funnel plot symmetry were done for the modelled associations of testosterone with predictors available in at least 10 studies.[25] In cases of significant asymmetry or if there were fewer than 10 studies we used the trim-and-fill method to estimate alternative summary estimates with imposed symmetry to evaluate the importance of asymmetry on results.[26]

Supplementary Results

IPD integrity and risk of bias within studies

Scores (total stars) from Newcastle-Ottawa Quality Assessments ranged from six to nine.[1] Relatively high scores largely reflected that these studies are: population-based; used a highly accurate method for measuring testosterone; adjusted for participant age and other risk factors; and (for subsequent planned analyses of prospective health outcomes) had at least five years of follow-up data. However, for the presented IPDMAs, there were several key limitations. For instance, the extent of statistical adjustment was limited to those covariates that were sought and available in IPD. Also, since these analyses were cross-sectional, results were potentially explainable by reverse causation.

Not all of the requested variables were supplied in IPD, and missing variables were either systematically imputed or datasets without the variable (i.e., if it was the dependent variable or predictor of interest) were excluded (Table S2). In the case of variables used for exclusions, however, neither of these options were considered appropriate. For instance, history of orchidectomy, which was used for exclusions, was unavailable for several studies (ARIC, CHS, FHS and MrOS USA). Furthermore, a limited history of linked medical records was available for identifying past orchidectomies in MAILES data. And for EMAS, only history of testicular disease status was available for use as a proxy. It is possible that the absence of reliable orchidectomy data for some studies might have resulted in the retention of individuals with abnormal sex hormone measurements.

Within datasets, some of the hormone variables were completely missing. LH was missing from ARIC, CHS, FHS, and SHIP IPD; DHT was missing from ARIC, FHS, MrOS USA, and SHIP IPD; and estradiol was missing from ARIC and CHS IPD (Table S2). Predictor variables that were completely missing included: Higher Education for BHS; diastolic blood pressure for MrOS USA (Model 3); general health status for ARIC and BHS (Model 6); CVD for CHS (Model 7, an exclusion criterion); COPD for EMAS, FHS, and SHIP (Model 9) and psychotropic drug use for BHS and MrOS USA (Model 16; Table S6). Patterns in partially-missing variables (i.e., variable supplied but with some missing values) differed among the studies, with lowest missingness for participant age (complete across all studies) and higher missingness for some of the lifestyle (alcohol consumption and vigorous physical activity) and health status variables (creatinine level for EMAS and MAILES, lipid medication and psychotropic drug use for EMAS). Ignoring the completely missing variables, datasets were ranked, from lowest to highest, in percentage of cases with missing data as: MrOS USA, HIMS, CHS, ARIC, SHIP, EMAS, FHS, MAILES, BHS. Across studies, the percentage of

incomplete cases in merged datasets ranged from 2.5% (Model 1) to 18.9% (Model 9; Table S6). Although these percentages are not remarkably high, they appear to vary primarily as a consequence of whether the lifestyle variables with higher missingness from BHS data (alcohol consumption and vigorous physical activity) were included (i.e., Models 1 and 6 versus the rest). In the case of the Model 6 IPDMA, BHS data were excluded because the predictor of interest (general health status) was not available in those data. However, Sensitivity Analysis 1 shows that the inclusion of BHS data into that IPDMA, which involved imputations of the completely missing (general health status and education), as well as of the partially-missing (including alcohol and physical activity) variables, did not lead to substantively different estimates of the summary effects (Figs S11-15).

Data checking of IPD identified only a small number of values as errors or likely incorrect. One value of physical activity exceeded a duration of 10,000 minutes within a week and was set to missing. Four values of total testosterone concentration were higher than 2,000 ng/dL and were set to missing. Three values of diastolic blood pressure were less than 21.4 mm Hg and were set to missing. And one value of creatinine of 1,053 µmol/L was set to missing. Summary statistics for testosterone were comparable to published estimates. Therefore, there were no important issues identified from checking IPD. Although the AD statistics supplied by CHAMP and MrOS Sweden were generated using the same statistical models and software, it is acknowledged that, in general, AD statistics cannot be subject to the same level of independent scrutiny as those estimated from supplied IPD.[10]

The definitions for some variables differed among studies. In some cases, such as for baseline cognition and depression, the definitions were considered too disparate for harmonisation. For instance, depression was identified using hospital admissions ICD codes for ARIC and

BHS, self-report data for HIMS and MAILES, CES-D score and medication usage for FHS, an abbreviated 10-item CES-D score for CHS,[27] Beck's Depression Index (BDI-II) for EMAS,[28] medication usage for MrOS USA, and from CID-S interview for SHIP. The authors considered that validated instruments such as CES-D or BDI would likely have been more accurate than self-report or from using diagnosis codes or medication data, but any such differences in accuracy were unknown, so there was no clear rationale for possible down-weighting. For other variables, such as alcohol consumption and physical activity, it was possible to harmonise by transforming continuous variables to a common scale (grams of ethanol per day, minutes of vigorous-intensity activity per week), or by grouping categorical measures in relation to some threshold (19.2 g/d, 75 min/week), or both. The definitions, justifications and rationale for each harmonisation rule are presented in Table S3. However, in cases where the harmonisations resulted in discretisation of continuous measures, this resulted in loss of information for estimating the summary effects. It is also possible that when variables from different studies were collected by different methods, unknown influences of methodology might have affected results.

In some studies, education and physical activity data were recorded years earlier than the timing of blood sampling for hormone measurement. Education data for ARIC participants (baseline: Visit 4; 1996-99) were available at Visit 1 (1987-89), and for the FHS Offspring cohort (baseline: Exam 7; 1998-2001) were available at Exam 2 (1979-83). Physical activity data for ARIC participants were collected during Visit 3 (1993-95), whereas for CHS participants (baseline: Year 7; 1994-95) the measurements used were collected two years earlier (in Year 5). The use of this information assumes that there was little change in characteristics from the earlier dates until blood sampling. Therefore, longitudinal change in such characteristics might have affected results.

Another issue with temporal alignment concerns the historical extent of medical records for MAILES IPD. Although linked health data (hospital admissions data commencing 1 July 2001; emergency department presentations commencing 1 July 2003) were sought, these records were available for only a relatively limited lookback period, since the earliest baseline date (blood sampling) in MAILES IPD was 21 August 2002. Although prevalent health status was determined using other data sources when available (including self-report and medication usage data), for COPD there was only linked health data, which suggests that COPD histories might have been under-represented in MAILES participants.

Heterogeneity

Associations with testosterone

There was an appreciable level of relative heterogeneity for the association of testosterone with BMI, and the estimated change in testosterone for one SD increase in BMI around the reference value (27.5 kg/m^2) was consistently negative in sign among studies, ranging from -3.18 to -2.01 nmol/L. The shape of associations was consistent in that trends estimated higher testosterone for lower BMI, with the average steepness of non-linear trends appearing to vary slightly among studies (Supplementary Fig. S3b). The curve for MAILES demonstrated a relatively pronounced inflection at the second knot (25.0 kg/m^2), as compared to the other studies.

Associations with other androgens

Appreciable relative heterogeneity was demonstrated for non-linear associations of age modelled against SHBG, DHT, and estradiol. Effect sizes among studies ranged from -7.89 to 17.88 nmol/L, -0.80 to 7.82 pmol/L, and -0.01 to 0.35 nmol/L, respectively. The lowest effect

size for SHBG (-7.89 nmol/L for CHS) was negative in sign, although its 95% CI covered zero (-46.28 to 30.49) and for the majority of this study's observed age range (71-97.7 years) higher SHBG concentrations were estimated for older ages, as observed for the other studies (Supplementary Fig. S3c). Trends for estradiol with age varied among studies most notably in older men, although differences near the margins of age distributions should be interpreted with caution because data are typically sparse in the tails, where splines are constrained to linearity (Supplementary Fig. S3i). The 95% CI of the summary effect of estradiol with age covered zero (-1.69 to 7.02 pmol/L), as did the 95% CIs for MrOS USA, MAILES, HIMS, and SHIP effect sizes, but were above zero in other studies (BHS, EMAS, FHS). Compared to trends in DHT with BMI, the trends in DHT with age showed relatively small change among studies, with 95% CIs of the effect sizes covering zero in all cases except for EMAS (0.01 to 0.16 pmol/L; Supplementary Fig. S3g, h).

Other IPDMAs that demonstrated appreciable relative heterogeneity were the associations of: DHT with Current versus Never smokers; DHT with general health status; and LH with creatinine. Effect sizes for the mean difference (MD) of DHT between Current and Never (reference level) smokers ranged from -0.17 to 0.20 nmol/L, being negative in sign for two studies (CHS, HIMS) but positive in sign for the other three (BHS, EMAS, MAILES; Supplementary Fig. S5d). Notably, all 95% CIs were non-overlapping with zero, with effect estimates negative for studies of older men but positive for studies of younger men. Effect sizes for the MD of DHT between Fair, Poor, or Very Poor versus Good or Excellent (reference level) ranged from -0.14 to 0.09 nmol/L, although this was for four studies (Supplementary Fig. S8g). The 95% CI for the summary effect size of LH with creatinine covered zero, as did the range of effect size estimates among the five studies analysed (-0.76

– 0.96 IU/L) although all showed increasing LH for higher creatinine concentrations (>115 μmol/L; Supplementary Fig. S7f).

Sensitivity Analysis 1: Imputing the predictor of interest

All ninety-five percent confidence intervals of summary estimates calculated for "Excluded" and "Imputed" IPD were overlapping, which demonstrates that systematic imputation of the predictor of interest, as opposed to excluding datasets without that variable, had negligible impact on results (Figs. S12-16). The 95% CI for the association of estradiol with COPD was much broader for studies providing COPD only (BHS, HIMS, MrOS USA) than when calculated from IPD including systematically-imputed COPD (BHS, EMAS, FHS, HIMS, MrOS USA, SHIP; Fig. S16). This is likely a consequence of an increased precision in the latter estimate because it was calculated from six studies, as compared to three. However, the decision to impute these missing categorical predictors of interest did not impact on the substantive interpretation of estimates.

Ninety-five percent confidence envelopes of summary curves were also overlapping for the associations of testosterone, SHBG, LH, and estradiol with diastolic BP (Figs. S12-14, S16). Diastolic BP was missing only from MrOS USA IPD, so the IPD from that study was either excluded, or diastolic BP was systematically imputed. Imputations for systematic missingness were not required in DHT analyses because DHT was not available in IPD from MrOS USA. Conspicuously narrower 95% confidence envelopes were observed for summary curves of "Excluded" IPD than of "Imputed" IPD in the testosterone and LH analyses. One explanation is that in both cases relatively high noise-to-signal in the systematically-imputed IPD resulted in an overfitted restricted cubic spline. This might have arisen in part because SMCFCS

imputation is not a hierarchical method but imputes the missing diastolic BP as having come from an average study.

Sensitivity Analysis 2: Modelling age using a restricted cubic spline

Repetition of Model 1 IPDMAs using a cubic polynomial function for age, which avoids issues with the knot placements, estimated similar summary curves, corroborating the results from restricted cubic spline modelling (Fig. S11). Although summary curves from the cubic polynomial model demonstrated some differences in trend for the youngest and oldest participants in DHT and estradiol analyses, 95% confidence envelopes overlapped those of the restricted cubic spline model in both cases (Fig. S11d,e). This evidence supports the assumption that the use of restricted cubic splines fitted with the specified knot values, which are outside of the age range for some studies, had not impacted substantively on findings.

Sensitivity Analysis 3: Ethnicity

White only versus All (ethnicity variable supplied in IPD)

Associations of testosterone concentration with age and BMI were similar when data for non-White participants were excluded, as compared to when those data were included, for studies that supplied an ethnicity variable (Fig. S17a,b). Both trends closely approximated the 95% confidence envelopes from analyses of all IPD, which suggests that there is negligible sensitivity to ethnicity type for these estimated non-linear associations.

The 95% CIs of summary estimates for the subgroup of IPD that included an ethnicity variable were overlapping with those in that subgroup that had been recorded as White (Fig. S18: “Ethnicity=Any” versus “Ethnicity=White”). These summary estimates represent the mean difference (MD) in testosterone between men grouped into the reference level and men:

in a married or de facto relationship; with higher education; frequent alcohol consumption; lower physical activity; who were former smokers; current smokers; had a history of CVD; or of diabetes. In all such comparisons the results are substantively the same, except for current smokers, where the lower bound of the 95% CI for “Ethnicity=Any” is less than zero and the lower bound for “Ethnicity=White” is above zero (Fig. S18f). Note, however, that the point estimates of the summary effect were very similar (0.91 and 0.89 nmol/L, respectively). Furthermore, since the “Ethnicity=Any” estimate was a subgroup of all IPD based on the presence of an ethnicity variable and not on ethnicity type, it is possible that wider 95% CIs could be explained by reduced precision alone. Indeed, the summary estimate for all IPD (0.89, 95% CI: 0.36 to 1.42 nmol/L; n=21,074) is similar to that for the “Ethnicity=Any” subset (0.91, 95% CI: -0.18 to 2.00 nmol/L; n=9,191) but has a narrower 95% CI. These results provide further support the assumption of negligible sensitivity in results attributable to ethnicity status.

Subset comprised of almost all or entirely White participants versus All IPD

There was little difference in the estimated associations of testosterone with age or with BMI, as calculated separately for the various subgroups of White participants (Fig. S17c,d). Trends for White participants closely approximated the 95% confidence envelopes from all IPD, suggesting that ethnicity had negligible influence on these estimated associations.

The 95% CIs of summary estimates for White only participants (variously defined and sub-setted as “Ethnicity=White”, “All White”, or “Almost entirely”) were overlapping with those for all participants (95% CIs for all IPD shown as vertical lines; Fig. S18). In some cases, the 95% CIs for White only participants covered zero whereas the 95% CI for all IPD did not, representing substantive differences in inference for these estimates. However, the signs of

point estimates from White only and all IPD were the same when there was no overlap of the 95% CIs with zero in the main analysis (Fig. S18a,d-h). Differences in coverage of 95% CIs for White only participants could be attributable to wider 95% CIs and reduced precision when estimated for smaller subsets of IPD.

Sensitivity Analysis 4: Adjustments from the inclusion of additional model terms.

Summary effects for the associations of total testosterone with sociodemographic and lifestyle predictors estimated from more complex multivariable models are shown together with those estimated from simpler models in the main analysis for comparison in Appendix Table A3.

Sensitivity Analysis 5: Including aggregate data from additional studies

Aggregate data (AD) from CHAMP (n=1,659) and MrOS Sweden (n=2,416) studies comprised summary statistics (for Table 1) as well as coefficient and variance estimates, or covariance matrices, and plot data obtained by statisticians from each of these studies by fitting Models 1, 2, 7, and 10 to IPD using R scripts that had been supplied to them. The CHAMP study did not have the required alcohol consumption or physical activity variables, so meta-analysis results for associations of total testosterone with these predictors could not be estimated using CHAMP AD. Also, AD from CHAMP for associations of testosterone with smoking status, diabetes, and CVD were obtained by fitting Models 2, 7, and 10 without the terms for alcohol consumption and physical activity.

Estimates of summary effects from meta-analyses that incorporated AD from CHAMP and MrOS Sweden (*IPD + AD*) were similar to, and had overlapping 95% CIs with, those estimated from studies that supplied IPD (*IPD*) (Fig. 3). Since all studies identified from the

systematic review provided either IPD or AD, and similar findings were obtained from meta-analyses of IPD only or IPD with AD, this shows that the influence of availability bias on results from studies not providing IPD was negligible.

Funnel plots for assessing publication bias

Funnel plots are presented for those meta-analyses that were of all available estimates, including AD. With AD from additional studies, the numbers of available estimates for meta-analyses were 10-11 (Fig. S19).

The funnel plot for the association of testosterone with age shows estimates for CHAMP, CHS, HIMS, and MrOS Sweden being more imprecise than estimates for the other seven studies (Fig. S19a). This is primarily an artefact of the spline modelling approach used and differences in sampling design among studies. Along with MrOS USA, these four studies were of the oldest participants (aged ≥65 yr) and therefore estimates of spline coefficients had inflated standard errors because the first knot (at 40 yr) was outside of the observed age ranges. However, estimates for the other seven studies, which collectively comprised the full range of participant ages (17-99 yr), showed comparatively high precision, both within and between studies, and are thus relatively close to this summary estimate on the funnel plot, because estimated spline coefficients contributed greater weighting towards the summary curve. Removing CHAMP, CHS, HIMS, and MrOS Sweden from the analysis resulted in a summary estimate of -1.04 nmol/L (CI -1.39, -0.68), which was less steep but not substantively different from estimates calculated using all IPD and AD (-1.33 nmol/L, CI -1.66, -1.01) or all IPD (-1.24 nmol/L, CI -1.61, -0.87).

All other funnel plots show the estimate for HIMS (largest sample size) towards the top and

the estimate for CHS (smallest sample size) towards the bottom of the plot (except for Fig. S18j, which did not include CHS), consistent with a more typical pattern, in the absence of bias or heterogeneity. Although meta-analyses of testosterone with BMI demonstrated appreciable relative heterogeneity, there was no significant departure from asymmetry (Fig. S19b). There was also no departure from asymmetry for meta-analyses of testosterone with married / de facto status, higher education, alcohol consumption, physical activity, Current v Never smokers, or with CVD or diabetes (Fig. S19c-f,h-j).

However, a significant departure from asymmetry was detected for the meta-analysis of testosterone with Former v Never smokers (Fig. S19g). The CHS estimate was least precise but within the white contour (p > 10%), suggesting that this pattern of asymmetry might not be consistent with publication bias. Estimates are distributed from top left (HIMS) to bottom right (CHS) on this plot, and repeating this analysis after removing the two extreme estimates (CHS, HIMS) gave a higher summary estimate (-0.26 nmol/L, CI -0.38, -0.13) that was not substantively different from that calculated from IPD and AD (-0.31 nmol/L, CI -0.49, -0.13) or IPD only (-0.34 nmol/L, CI -0.55, -0.12). A corresponding estimate obtained using trim-and-fill (5 imputed studies added) was lower but not substantively different (-0.49 nmol/L, CI -0.70, -0.28), further suggesting that the detected asymmetry was not likely important.

Exploratory subgroup analysis of men without common age-associated medical comorbidities

This analysis explored the possibility that the observed non-linear declines in baseline testosterone, and increases in LH, with the age in men older than 70 years might be due to increased presence of comorbidities in those older men. Summary curves were generated from two-stage IPD meta-analyses as described in the Methods and Supplementary Methods. IPD meta-analyses of testosterone with age, and of LH with age, were repeated by fitting the

models to the subgroup of men free of health conditions that are known to be age-related diseases and had prevalent status recorded in available IPD. Two such ‘disease-free’ subgroups were constructed:

(i) Subgroup A: Men free of hypertension, diabetes, CVD, cancer, COPD, who were not taking lipid-lowering medications, and had serum creatinine ≤ 150 µmol/L;

(ii) Subgroup B: Men free of hypertension, diabetes, CVD, cancer, who were not taking lipid-lowering medications, and had serum creatinine ≤ 150 µmol/L.

IPD meta-analyses of Subgroup A could not use all IPD because COPD status was not available in three of the studies (EMAS, FHS, SHIP). Therefore, a larger amount of IPD was analysed for Subgroup B, using IPD from all studies with measurements on the hormone variable, with the limitation that results from Subgroup B ignore the potential influence of COPD status. Furthermore, the IPD meta-analyses of Subgroups A and B were done using Model 2, which controls for additional model terms of modifiable lifestyle factors (baseline alcohol consumption, vigorous physical activity, and smoking status). Summary curves with 95% confidence intervals (CIs) and 95% prediction intervals from the subgroup IPD meta-analyses are overlaid on plots with those from the main analysis.

The estimated summary association for testosterone with age at baseline for disease-free subgroups showed a similar non-linear trend to that estimated from all IPD in the main analysis, although the decline with age was less steep and 95% CIs were overlapping with estimated testosterone at the reference age, across all ages (Figs. S20a, S21a). However, results for the association of LH with age were substantively consistent with those of the main analyses, with the summary curve, CIs and 95% prediction intervals showing an increasing trend with baseline age for men older than 70 years (Figs. S20b, S21b).

Funding statements for individual studies included in this meta-analysis

The ARIC study is funded by has been funded in whole or in part with Federal funds from the National Heart, Lung, and Blood Institute, National Institutes of Health, Department of Health and Human Services, under Contract nos. (75N92022D00001, 75N92022D00002, 75N92022D00003, 75N92022D00004, 75N92022D00005) and Dr. Ballantyne received NIH grant support through R01HL134320. The 1994/1995 Busselton Health Survey was supported by the Western Australian Health Promotion Foundation. The Cardiovascular Health Study was supported by contracts HHSN268201200036C, HHSN268200800007C, HHSN268201800001C, N01HC55222, N01HC85079, N01HC85080, N01HC85081, N01HC85082, N01HC85083, N01HC85086, 75N92021D00006, and grants U01HL080295 and U01HL130114 from the National Heart, Lung, and Blood Institute (NHLBI), with additional contribution from the National Institute of Neurological Disorders and Stroke (NINDS). Additional support was provided by R01AG023629 from the National Institute on Aging (NIA). A full list of principal CHS investigators and institutions can be found at CHS-NHLBI.org. The content is solely the responsibility of the authors and does not necessarily represent the official views of the National Institutes of Health. The EMAS Study is funded by The Commission of the European Communities Fifth Framework Programme 'Quality of Life and Management of Living Resources' Grant No. QLK6-CT- 2001-00 258 and supported by Arthritis Research UK. The Framingham Heart Study is conducted and supported by the National Heart, Lung, and Blood Institute (NHLBI) in collaboration with Boston University (Contract No. N01-HC-25195, HHSN268201500001I and 75N92019D00031). This manuscript was not prepared in collaboration with investigators of the Framingham Heart Study and does not necessarily reflect the opinions or views of the Framingham Heart Study, Boston University, or NHLBI.

The HIMS study is funded by project grants from the National Health and Medical Research Council of Australia. The MAILES Study is funded by a project grant from the National Health and Medical Research Council of Australia (627227). The MrOS Sweden Study is funded by The Swedish Research Council and Swedish ALF funding. The Osteoporotic Fractures in Men (MrOS) Study is supported by National Institutes of Health funding. The following institutes provide support: the National Institute on Aging (NIA), the National Institute of Arthritis and Musculoskeletal and Skin Diseases (NIAMS), the National Center for Advancing Translational Sciences (NCATS) and NIH Roadmap for Medical Research under the following grant numbers: U01 AG027810, U01 AG042124, U01 AG042139, U01 AG042140, U01 AG042143, U01 AG042145, U01 AG042168, U01 AR066160 and UL1 TR000128. SHIP is part of the Community Medicine Research network of the University of Greifswald, Germany, which is funded by the Federal Ministry of Education and Research (grants 01ZZ9603, 01ZZ0103 and 01ZZ0403), the Ministry of Cultural Affairs, as well as the Social Ministry of the Federal State of Mecklenburg–West Pomerania.

Supplementary Tables

Table S1. PRISMA-IPD Checklist of items to include when reporting a systematic review and meta-analysis of individual participant data (IPD): the Androgens In Men Study (AIMS).

PRISMA-IPD Section/topic	Item No	Checklist item	Reported on page
**Title**			
Title	1	Identify the report as a systematic review and meta-analysis of individual participant data.	p1 (The systematic review is cited[a] and published elsewhere)
**Abstract**			
Structured summary	2	Provide a structured summary including as applicable:	p4-5
		**Background**: state research question and main objectives, with information on participants, interventions, comparators and outcomes.	
		**Methods**: report eligibility criteria; data sources including dates of last bibliographic search or elicitation, noting that IPD were sought; methods of assessing risk of bias.	
		**Results**: provide number and type of studies and participants identified and number (%) obtained; summary effect estimates for main outcomes (benefits and harms) with confidence intervals and measures of statistical heterogeneity. Describe the direction and size of summary effects in terms meaningful to those who would put findings into practice.	
		**Discussion**: state main strengths and limitations of the evidence, general interpretation of the results and any important implications.	
		**Other**: report primary funding source, registration number and registry name for the systematic review and IPD meta-analysis.	
**Introduction**			
Rationale	3	Describe the rationale for the review in the context of what is already known.	p6-7

PRISMA-IPD Section/topic	Item No	Checklist item	Reported on page
Objectives	4	Provide an explicit statement of the questions being addressed with reference, as applicable, to participants, interventions, comparisons, outcomes and study design (PICOS). Include any hypotheses that relate to particular types of participant-level subgroups.	p6; For this type of study it is PEO;[b] Appendix Table A2.
**Methods**			
Protocol and registration	5	Indicate if a protocol exists and where it can be accessed. If available, provide registration information including registration number and registry name. Provide publication details, if applicable.	p8
Eligibility criteria	6	Specify inclusion and exclusion criteria including those relating to participants, interventions, comparisons, outcomes, study design and characteristics (e.g. years when conducted, required minimum follow-up). Note whether these were applied at the study or individual level i.e. whether eligible participants were included (and ineligible participants excluded) from a study that included a wider population than specified by the review inclusion criteria. The rationale for criteria should be stated.	p8
Identifying studies - information sources	7	Describe all methods of identifying published and unpublished studies including, as applicable: which bibliographic databases were searched with dates of coverage; details of any hand searching including of conference proceedings; use of study registers and agency or company databases; contact with the original research team and experts in the field; open adverts and surveys. Give the date of last search or elicitation.	p8 (citation[a]).
Identifying studies - search	8	Present the full electronic search strategy for at least one database, including any limits used, such that it could be repeated.	p8 (citation[a])
Study selection processes	9	State the process for determining which studies were eligible for inclusion.	p8 (citation[a])
Data collection processes	10	Describe how IPD were requested, collected and managed, including any processes for querying and confirming data with investigators. If IPD were not sought from any eligible study, the reason for this should be stated (for each such study).	p8, 10 & Supplementary Methods.
		If applicable, describe how any studies for which IPD were not available were dealt with. This should include whether, how and what aggregate data were sought or extracted from study reports and	

PRISMA-IPD Section/topic	Item No	Checklist item	Reported on page
		publications (such as extracting data independently in duplicate) and any processes for obtaining and confirming these data with investigators.	
Data items	11	Describe how the information and variables to be collected were chosen. List and define all study level and participant level data that were sought, including baseline and follow-up information. If applicable, describe methods of standardising or translating variables within the IPD datasets to ensure common scales or measurements across studies.	p8-10 & Suppl. Tables S2, S3
IPD integrity	A1	Describe what aspects of IPD were subject to data checking (such as sequence generation, data consistency and completeness, baseline imbalance) and how this was done.	p9 & Suppl. Methods
Risk of bias assessment in individual studies.	12	Describe methods used to assess risk of bias in the individual studies and whether this was applied separately for each outcome. If applicable, describe how findings of IPD checking were used to inform the assessment. Report if and how risk of bias assessment was used in any data synthesis.	p8 (citation[a]) & Suppl. Methods (Suppl. p4)
Specification of outcomes and effect measures	13	State all treatment comparisons of interests. State all outcomes addressed and define them in detail. State whether they were pre-specified for the review and, if applicable, whether they were primary/main or secondary/additional outcomes. Give the principal measures of effect (such as risk ratio, hazard ratio, difference in means) used for each outcome.	p8-12 (citation[a])
Synthesis methods	14	Describe the meta-analysis methods used to synthesise IPD. Specify any statistical methods and models used. Issues should include (but are not restricted to):<br>• Use of a one-stage or two-stage approach.<br>• How effect estimates were generated separately within each study and combined across studies (where applicable).<br>• Specification of one-stage models (where applicable) including how clustering of patients within studies was accounted for.<br>• Use of fixed or random effects models and any other model assumptions, such as proportional hazards.<br>• How (summary) survival curves were generated (where applicable).<br>• Methods for quantifying statistical heterogeneity (such as I^2 and τ^2).	p10-12 & Suppl. Methods

PRISMA-IPD Section/topic	Item No	Checklist item	Reported on page
		• How studies providing IPD and not providing IPD were analysed together (where applicable).<br>• How missing data within the IPD were dealt with (where applicable).	
Exploration of variation in effects	A2	If applicable, describe any methods used to explore variation in effects by study or participant level characteristics (such as estimation of interactions between effect and covariates). State all participant-level characteristics that were analysed as potential effect modifiers, and whether these were pre-specified.	p8-10, Appendix & Suppl. Methods
Risk of bias across studies	15	Specify any assessment of risk of bias relating to the accumulated body of evidence, including any pertaining to not obtaining IPD for particular studies, outcomes or other variables.	p11-12 & Suppl. Methods
Additional analyses	16	Describe methods of any additional analyses, including sensitivity analyses. State which of these were pre-specified.	p10, 12 & Suppl. Methods
**Results**			
Study selection and IPD obtained	17	Give numbers of studies screened, assessed for eligibility, and included in the systematic review with reasons for exclusions at each stage. Indicate the number of studies and participants for which IPD were sought and for which IPD were obtained. For those studies where IPD were not available, give the numbers of studies and participants for which aggregate data were available. Report reasons for non-availability of IPD. Include a flow diagram.	p8 & Suppl. Fig. S1 & citation[a]
Study characteristics	18	For each study, present information on key study and participant characteristics (such as description of interventions, numbers of participants, demographic data, unavailability of outcomes, funding source, and if applicable duration of follow-up). Provide (main) citations for each study. Where applicable, also report similar study characteristics for any studies not providing IPD.	Appendix Table A1. Funding statements in Supp. Material.
IPD integrity	A3	Report any important issues identified in checking IPD or state that there were none.	p8, Suppl. Results
Risk of bias within studies	19	Present data on risk of bias assessments. If applicable, describe whether data checking led to the up-weighting or down-weighting of these assessments. Consider how any potential bias impacts on the robustness of meta-analysis conclusions.	Suppl. Results (Suppl. p14-18)
Results of individual studies	20	For each comparison and for each main outcome (benefit or harm), for each individual study report the number of eligible participants for which data were obtained and show simple summary data for each	Appendix Table A1, Figs 1-2,

PRISMA-IPD Section/topic	Item No	Checklist item	Reported on page
		intervention group (including, where applicable, the number of events), effect estimates and confidence intervals. These may be tabulated or included on a forest plot.	Suppl. Figs S2-S10.
Results of syntheses	21	Present summary effects for each meta-analysis undertaken, including confidence intervals and measures of statistical heterogeneity. State whether the analysis was pre-specified, and report the numbers of studies and participants and, where applicable, the number of events on which it is based.	Table 1, Appendix Table A3, p12-16 & Suppl. Results
		When exploring variation in effects due to patient or study characteristics, present summary interaction estimates for each characteristic examined, including confidence intervals and measures of statistical heterogeneity. State whether the analysis was pre-specified. State whether any interaction is consistent across trials.	
		Provide a description of the direction and size of effect in terms meaningful to those who would put findings into practice.	
Risk of bias across studies	22	Present results of any assessment of risk of bias relating to the accumulated body of evidence, including any pertaining to the availability and representativeness of available studies, outcomes or other variables.	p16 & Suppl. Fig S19, Suppl. Results
Additional analyses	23	Give results of any additional analyses (e.g. sensitivity analyses). If applicable, this should also include any analyses that incorporate aggregate data for studies that do not have IPD. If applicable, summarise the main meta-analysis results following the inclusion or exclusion of studies for which IPD were not available.	p17 & Fig. 3 & Appendix Table A3 & Suppl. Figs S11-18, Suppl. Results
**Discussion**			
Summary of evidence	24	Summarise the main findings, including the strength of evidence for each main outcome.	p16-19
Strengths and limitations	25	Discuss any important strengths and limitations of the evidence including the benefits of access to IPD and any limitations arising from IPD that were not available.	p20-21
Conclusions	26	Provide a general interpretation of the findings in the context of other evidence.	p20-22
Implications	A4	Consider relevance to key groups (such as policy makers, service providers and service users). Consider implications for future research.	P21-22

PRISMA-IPD Section/topic	Item No	Checklist item	Reported on page
**Funding**			
Funding	27	Describe sources of funding and other support (such as supply of IPD), and the role in the systematic review of those providing such support.	p12, Appendix

a. Marriott RJ, Harse J, Murray K, Yeap BB. Systematic review and meta-analyses on associations of endogenous testosterone concentration with health outcomes in community-dwelling men. *BMJ Open* 2021;11:e048013.
b. Munn Z, Stern C, Aromataris E, et al. What kind of systematic review should I conduct? A proposed typology and guidance for systematic reviewers in the medical and health sciences. *BMC Med Res Methodol* 2018;18:5.

A1 – A3 denote new items that are additional to standard PRISMA items. A4 has been created as a result of re-arranging content of the standard PRISMA statement to suit the way that systematic review IPD meta-analyses are reported.

© Reproduced with permission of the PRISMA IPD Group, which encourages sharing and reuse for non-commercial purposes

Table S2. Harmonised variables summary for AIMS IPD Meta-analysis 1: IPD-level data. Red font = imputations done for systematic missingness in some studies. Blue font = not analysed (see table footnote for explanation[a]).[1]

Variable[b]	Type	Ref.	ARIC	BHS	CHS	EMAS	FHS	HIMS	MAILES	MrOS USA	SHIP
Dependent variable											
Testosterone	C	N/A	✓	✓	✓	✓	✓	✓	✓	✓	✓
SHBG	C	N/A	✓	✓	✓	✓	✓	✓	✓	✓	✓
LH	C	N/A		✓		✓		✓	✓	✓	
DHT	C	N/A		✓	✓	✓		✓	✓		
Estradiol	C	N/A		✓		✓	✓	✓	✓	✓	✓
Socio-demographic											
Age	C	70 yr	✓	✓	✓	✓	✓	✓	✓	✓	✓
Higher Education	D	Not university degree or equivalent	✓		✓	✓	✓	✓	✓	✓	✓
Ethnicity			✓		✓		✓		✓	✓	
Marital status	D	Not married or not defacto	✓	✓	✓	✓	✓	✓	✓	✓	✓
Site or sub-cohort			✓		✓	✓	✓		✓	✓	✓
BMI	C	27.5 kg/m^2	✓	✓	✓	✓	✓	✓	✓	✓	✓
Waist			✓	✓	✓	✓	✓	✓	✓		✓
Lifestyle											
Alcohol consumption	D	<19.2 g/day	✓	✓	✓	✓	✓	✓	✓	✓	✓
Physical activity (vigorous level)	D	>75 mins/wk	✓	✓	✓	✓	✓	✓	✓	✓	✓
Smoking status	D	Never smoked	✓	✓	✓	✓	✓	✓	✓	✓	✓
Health condition / status											
Diastolic BP	C	80 mm Hg	✓	✓	✓	✓	✓	✓	✓		✓
Systolic BP	C	135 mm Hg	✓	✓	✓	✓	✓	✓	✓	✓	✓
Hypertension	D	No	✓	✓	✓	✓	✓	✓	✓	✓	✓
General health	D	Good or Excellent			✓	✓	✓	✓	✓	✓	✓
Prevalent CVD	D	No	✓	✓		✓	✓	✓	✓	✓	✓
Prevalent cancer	D	No	✓	✓	✓	✓	✓	✓	✓	✓	✓
Prevalent dementia			✓	✓	✓		✓	✓			
Baseline cognition					✓	✓	✓	✓			
COPD	D	No	✓	✓	✓			✓	✓	✓	
Diabetes	D	No	✓	✓	✓	✓	✓	✓	✓	✓	✓
Cholesterol / HDL[c]	C	4.3	✓	✓	✓	✓	✓	✓	✓	✓	✓
LDL	C	3.2 mmol/L	✓	✓	✓	✓	✓	✓	✓	✓	✓
HDL	C	1.3 mmol/L	✓	✓	✓	✓	✓	✓	✓	✓	✓
Creatinine level	C	90 µmol/L	✓	✓	✓	✓	✓	✓	✓	✓	✓
Lipid lowering medications	D	Not taking	✓	✓	✓	✓	✓	✓	✓	✓	✓
Anxiety								✓	✓		✓
Depression			✓	✓	✓	✓	✓	✓	✓	✓	✓
Psychotropic drug use	D	No	✓		✓	✓	✓	✓	✓		✓

Abbreviations: 'Type' = C, continuous; D, discrete categories. 'Ref' = reference level for calculating effect size. For C variables Ref. is a reference value (approximate average across studies); for D variables Ref. is the reference level chosen for modelling.

[1] Table S2 footnotes are continued over the page.

a. Variables in blue font deemed not suitable for inclusion in Analysis 1 because: (i) the majority of data is for white males therefore a subgroup analysis will explore the sensitivity to results for modelling white males only instead (for ethnicity); (ii) site or sub-cohort is specific to each study and obtained for the purpose of *post hoc* investigations of heterogeneity where applicable[20]; (iii) waist circumference is highly correlated with BMI in all models (as consistent with Yeap et al.[19]); (iv) baseline cognition and depression were variously quantified among studies and thus could not be harmonised with confidence; (v) prevalent dementia, baseline cognition, and anxiety were obtained from too few studies. Furthermore, for those studies where it was provided, prevalent dementia was sparsely distributed (e.g., n=1 prevalent case in each of the ARIC and FHS datasets, after exclusions).
b. As listed in the Table 1 column for Analysis 1 in the protocol article.[20]
c. Total cholesterol: HDL ratio is used in preference to total cholesterol, as it is an improved indicator of risk to heart disease.[29]

Table S3. Definitions of harmonized variables.

Variable name	Definition	Justification/Rationale
Higher Education	"University degree or equivalent" vs. "Not" (Ref.)	The attainment of a tertiary level qualification, defined as a university degree or equivalent (at least four years of college in the US system) or higher (e.g., post-graduate) was provided for all IPD-level datasets although primary, secondary, and vocational education were variously reported. Accordingly, we constructed a binary categorical variable grouping "University Degree or Equivalent" versus "Not" for education (highest level qualification attained).
Marital status	"Married or Defacto" vs. "Not" (Ref.)	"Defacto" status is not reported as a category by all studies, although "Married" is. However, "Married" and "Defacto" were combined within the same category in SHIP data. Other marital status categories were variously reported among the studies. Therefore we constructed a binary categorical variable grouping "Married or Defacto" versus "Not" values for marital status, where "Not" includes: divorced, never married, separated, widowed, "formerly married or cohabit", single, "separated or divorced", "other".
Alcohol consumption	Average alcohol consumption per day as 2 categories: <19.2 g (Ref.) vs. ≥19.2 g.	EMAS provided the number of days per week participants had consumed alcohol. Dichotomizing to <5 v ≥5 days per week was suggested, as this is often used by EMAS researchers to separate non-drinkers/infrequent from frequent alcohol drinkers. Using a reported estimate of 26.9 g ethanol consumed per day in the EU,[30] we obtain a corresponding threshold for 5 days of average EU alcohol consumption to be 134.5 g, or 19.2 g per day, as distributed across the week. MAILES provided the number of alcoholic drinks per day in categories, and we approximated the threshold of 19.2 g as 2 Australian alcoholic drinks for that study. For FHS we obtained the number of alcoholic drinks per week, and we

Variable name	Definition	Justification/Rationale
		approximated this threshold as 1.37 US alcoholic drinks per week. Alcohol consumption in g/week could be calculated for all of the other study cohorts using the variables provided.
Physical activity	Duration of vigorous-intensity activity, *va*, as 2 categories: $va > 75$ mins per week (Ref.) vs. $va \leq 75$ mins per week.	The 75-minute threshold is consistent with the WHO "Global Recommendations on Physical Activity for Health"[31] for the total duration of vigorous-intensity physical activities per week, to improve cardiorespiratory and muscular fitness, bone health, reduce the risk of non-communicable diseases and depression.<br>Vigorous-intensity activity was specified because HIMS provided only the number of hours of vigorous vs. non-vigorous activity per week. BHS, FHS, HIMS collected self-report estimates of time spent doing activities of different intensity. For MAILES we used durations for activities that resulted in "a large increase in heart rate or breathing". All other studies requested a duration estimate for a specified type of activity. ARIC and SHIP provided the time spent playing sport (either in categories or as continuous variables), and we took the approximation that >2 hours of sport for ARIC or SHIP participants as equivalent to >75 mins of vigorous intensity activity. Where durations for different types of activity were provided (CHS), we consulted the Compendium of Physical Activities to identify vigorous activities as those with a METS ≥ 6.[32] For EMAS and MrOS USA, we used values for activities categorised as strenuous sports and muscle strength/endurance, which had been recorded for the purpose of calculating PASE scores.[33]
Smoking status	"Never"(Ref.) vs. "Former" vs. "Current" smoker.	
Hypertension	Use the available definition for that study, if one exists. This may or may not be derived wholly or in part from self-report or evidence of taking applicable medications. If	

Variable name	Definition	Justification/Rationale
	required to specify in terms of blood pressure thresholds, we used Diastolic BP ≥ 90 mm Hg or Systolic BP ≥ 140 mm Hg.	
General health	"Good or Excellent" (Ref.) vs. "Not"	MrOS USA provided "Poor/ Very Poor / Fair" vs. "Good / Excellent" so individual categories for health status provided by the other studies were pooled commensurately to form the same binary health status categorisation.
Prevalent CVD	Use the available definition for that study, if one exists. This may or may not be derived wholly or in part from self-report data. If required to specify, in terms of ICD-10 codes, we used I20.0, I21-I24, I50, I60, I61, I63, I64 and I69.0-I69.4 or these ICD-9 codes: 410, 411.0, 411.1, 411.81, 411.89, 428, 429.79, 430, 431, 433-438.	
Prevalent cancer	Use the available definition for that study, if one exists. Specified to non-skin cancers, if possible (ICD-10 codes C00-43, C45-C97 or ICD-9 codes 140-172, 175-209).	
COPD	Use the available definition for that study, if one exists. This may or may not be derived wholly or in part from self-report data. Spirometry variables (FEV1, FVC) were used when available. If required to specify, in terms of ICD-10 codes, we used J43.1, J43.2, J43.8, J43.9, J44.0, J44.1, J44.8, J44.9 or these ICD-9 codes: 490-492, 494, 496.	
Diabetes	Use the available definition for that study, if one exists. This may or may not be derived wholly or in part from self-report or evidence of taking applicable medications, fasting glucose or HbA1c measurements. If fasting blood glucose measurements were available, we used the threshold of ≥ 7 mmol/L.	

Variable name	Definition	Justification/Rationale
Lipid-lowering medications	Use the available definition for that study, if one exists. Or picked from list of medications recorded. Otherwise specified using ATC codes.	
Psychotropic drug use	Use the available definition for that study, if one exists. Or picked from list of medications recorded. Otherwise specified using ATC codes.	

Table S4A. Recruitment criteria for participants (community-dwelling men) in each study cohort.

Study	Blood sampling	Fasting	Age criterion	Dementia	Mobility criterion	Notes
ARIC	Morning[1]	Most[1]	45-64[2]	Included	None	1. In the analyses published by Srinath et al.[34] and Srinath et al.[35] all samples obtained after 10:30am were excluded and it was stated that venipuncture (for mass spectrometry measurements of T) was performed in a fasting state. In the IPD analysed n=1,506 (97%) out of 1,550 participants had a fasting time of at least 8 hours recorded, with fasting time missing for 6 participants, and n=1,371 (95%) out of 1,446 participants had recorded blood sampling before 11AM, with missing times for 110 participants.<br>2.Restriction applied to cohort component initially recruited (1987-1989). However, this study analysed IPD from Visit 4 (1996-1998).
BHS	Early morning	Yes	≥18	Included	None	Surviving members of cross-sectional surveys in Busselton from 1966-1987 were invited to participate.[36]
CHS	Most before noon	No[3]	≥65	Included	Excluded wheelchair-bound participants	Also excluded men treated for cancer at initial enrolment (1989-1990) although this study analysed IPD from the 1994 visit as consistent with Shores et al.,[9] which excludes participants with history of cardiovascular disease at that study. Shores et al.[9] also excluded participants with prostate cancer in 1994 but that exclusion was not applied in this analysis.<br>3. In the IPD analysed n=124 (11%) out of 1,123 participants analysed had fasted at least 8 hours prior to blood sampling.
EMAS	Before 10AM	Yes	40-79	Included	None	Initial recruitment was targeted to recruit equal numbers of participants into each of four 10-year age bands.
FHS	Typically 7:30-8:30AM	Yes	None[4]	Included	None	4. Comprised of the second and third generations of the original cohort of participants, who were 30-62 years old, following Bhasin et al.[37]

Study	Blood sampling	Fasting	Age criterion	Dementia	Mobility criterion	Notes
HIMS	8-10:30AM or early morning	No[5]	≥65[6]	Included	None	5. Norman et al.[38] reported that n=3,328 (78%) out of 4,249 men were fasting although Chan et al.[39] reported an analysis of only early morning fasting blood samples. In the IPD analysed n=3,230 (78%) out of 4,121 participants were fasted.<br>6. Men aged 65 or older were recruited into the randomized trial in 1996-1999 although this study analysed IPD from the 2001-2004 follow-up survey, when blood samples were obtained.
MAILES	Before 11AM	Yes	35-80	Excluded[7]	None	7. Excluded men who were of insufficient mental or physical ability to understand requirements of participation or to adequately participate, or were ill or otherwise incapacitated to attend clinics.[40]
MrOS USA	Morning[8]	Yes[9]	≥65	Included	No bilateral hip replacement	8. In the IPD analysed n=1,872 (94%) out of 2,001 participants had blood samples taken before 11AM, with missing time for one participant.<br>9. In the IPD analysed n=1,935 (97%) out of 2,002 participants were fasted.<br>Also excluded men with a medical condition that (in the judgement of the investigator) would result in imminent death.[41]

Study	Blood sampling	Fasting	Age criterion	Dementia	Mobility criterion	Notes
SHIP	8am-7pm[10]	No[11]	20-79	Included	None	10. Kische et al.[42] reported that additional adjustment for time and date of blood sampling did not substantially alter estimates of associations of androgens with depressive symptoms and cognitive status in cross-sectional analyses. In the IPD analysed n=1,192 (57%) out of 2,109 participants had blood samples taken before 11AM, with missing time for one participant.<br>11. Fasting status was available for SHIP-TREND (2008-2011; n=437) but not SHIP-0 (1997-2001; n=1,673) of the IPD provided and analysed together for this study. In the IPD analysed n=400 (92%) out of 437 participants with fasting status recorded had fasted at least 8 hours prior to blood sampling.
CHAMP	Early morning[12]	Yes	≥70	Included	None	12. Hsu et al.,[43] Hsu et al.[44] and Hsu et al.[45] analysed early morning fasting blood samples. Cumming et al.[46] reported that subjects with an afternoon appointment had blood samples taken in their own homes, usually on the same day as their clinic appointment.
MrOS Sweden	Morning or around noon[13]	Yes (1 out of 3 cities)[14]	69-81[47]	Included	No bilateral hip replacement	13. Serum samples drawn in the morning (before 10am; 69% of the cohort) or around noon (between 10am and 3pm, average 1pm; 31%).<br>14. Gothenburg (n=905; 37%) had fasting morning samples, whereas the other two cities (Malmö and Uppsala; 63%) did not.

Table S4B. Recruitment criteria for participants (community-dwelling men) in each study cohort identified as potentially eligible in the bridge search.

Study	Blood sampling	Fasting	Age criterion	Dementia	Mobility criterion	Notes
NHANES 2011-14	Morning/afternoon/ evening	No	≥20	Included	None	15. n=1,780 men in the 2011-14 wave had testosterone measured using mass spectrometry, men in the 1998-91 and 1999-2004 waves had testosterone measured using immunoassay.[48,49]
Henan Rural Cohort Study	After overnight fast	Yes	18-79	Included	None	16. n=2,586 men, blood samples collected after at least 8-hr overnight fasting. Cross-sectional analyses reported, but authors indicate prospective follow-up planned.[50,51]

None of these publications[48-51] contained summary estimates relevant to the IPDMA. These two cohorts could be approached for further information to determine eligibility for inclusion should a new IPDMA be conducted in the future.

Table S5. Values used for spline modelling of continuous predictors: knot points for spline modelling and standard deviations used for effect size calculations.

predictor	units	For spline modelling*	For effect size calculation			
		Knot points	SD	Ref. value**	From	To
Age	year	40, 70, 80	15.6	70.0	62.20	77.80
Age: younger (17-70 yr)	year		15.6	43.5	35.70	51.30
Age: older (70-99 yr)	year		15.6	84.5	76.70	92.30
BMI	kg/m^2	21.6, 25.0, 27.1, 29.4, 34.8	4.11	27.5	25.45	29.56
Diastolic BP	mm Hg	60.0, 72.0, 79.0, 85.0, 99.5	11.9	80.0	74.05	85.95
Systolic BP	mm Hg	108.0, 123.0, 134.5, 148.0, 174.5	20.7	135.0	124.65	145.35
Cholesterol / HDL	dimensionless	2.5, 3.4, 4.1, 4.9, 6.8	1.4	4.3	3.60	5.00
LDL	mmol/L	1.8, 2.6, 3.1, 3.7, 4.8	0.9	3.2	2.75	3.65
HDL	mmol/L	0.8, 1.0, 1.2, 1.4, 1.9	0.36	1.3	1.12	1.48
Creatinine	μmol/L	62.0, 78.0, 88.0, 97.3, 123.9	26.8	90.0	76.60	103.40

* = Restricted cubic splines were fitted with pre-specified knot values at the 5th, 27.5th, 50th, 72.5th, and 95th percentiles using the entire IPD across studies[11] except for age, which had knots placed at suitable values following inspection of distributions by study (at the 11.5th, 60th and 88.5th percentiles). Since age distributions varied across studies (see Supplemental Methods), the first and second knots were placed at values where data ended in some studies, and the second knot value for age was observed in participants from all studies.

** = The reference value was the covariate mean from the entire distribution of IPD except for age, which was centred at a value within the narrow range (i.e., 70-74 yr) common to all studies. The reference value was also used for centering each corresponding covariate.

Table S6. Missing data summary. Number of missing values per variable and dataset for each IPDMA of total testosterone.

Model	Study	n	Age	BMI	Married/ De Facto	Higher Education	Alcohol	Smoking	Physical Activity	Health condition *j**	Incomplete cases (%)	
											**Study****	**IPDMA*****
1	ARIC	1,556	0	0	0	2					0.13	2.45
1	BHS	2,021	0	64	62	**2,021**					5.10	2.45
1	CHS	1,123	0	1	4	2					0.62	2.45
1	EMAS	2,832	0	39	83	53					5.33	2.45
1	FHS	3,334	0	3	6	218					6.66	2.45
1	HIMS	4,121	0	16	4	4					0.53	2.45
1	MAILES	1,975	0	3	6	30					1.67	2.45
1	MrOS USA	2,002	0	1	0	0					0.05	2.45
1	SHIP	2,110	0	4	8	25					1.37	2.45
2	ARIC	1,556	0	0	0	2	2	1	56		3.86	14.43
2	BHS	2,021	0	64	62	**2,021**	343	100	760		46.21	14.43
2	CHS	1,123	0	1	4	2	0	1	17		2.23	14.43
2	EMAS	2,832	0	39	83	53	20	58	45		8.26	14.43
2	FHS	3,334	0	3	6	218	0	5	111		9.66	14.43
2	HIMS	4,121	0	16	4	4	0	2	8		0.73	14.43
2	MAILES	1,975	0	3	6	30	124	15	52		9.92	14.43
2	MrOS USA	2,002	0	1	0	0	2	0	0		0.15	14.43
2	SHIP	2,110	0	4	8	25	132	10	11		7.16	14.43
3	ARIC	1,556	0	0	0	2	2	1	56	0	3.86	16.01
3	BHS	2,021	0	64	62	**2,021**	343	100	760	57	46.26	16.01
3	CHS	1,123	0	1	4	2	0	1	17	1	2.32	16.01
3	EMAS	2,832	0	39	83	53	20	58	45	21	8.30	16.01
3	FHS	3,334	0	3	6	218	0	5	111	2	9.69	16.01
3	HIMS	4,121	0	16	4	4	0	2	8	6	0.85	16.01
3	MAILES	1,975	0	3	6	30	124	15	52	4	10.13	16.01

Model	Study	n	Age	BMI	Married/ De Facto	Higher Education	Alcohol	Smoking	Physical Activity	Health condition *j**	Incomplete cases (%)	
											**Study****	**IPDMA*****
3	MrOS USA	2,002	0	1	0	0	2	0	0	**2,002**	0.15	16.01
3	SHIP	2,110	0	4	8	25	132	10	11	3	7.30	16.01
4	ARIC	1,556	0	0	0	2	2	1	56	0	3.86	14.66
4	BHS	2,021	0	64	62	**2,021**	343	100	760	56	46.26	14.66
4	CHS	1,123	0	1	4	2	0	1	17	0	2.23	14.66
4	EMAS	2,832	0	39	83	53	20	58	45	20	8.26	14.66
4	FHS	3,334	0	3	6	218	0	5	111	1	9.66	14.66
4	HIMS	4,121	0	16	4	4	0	2	8	1	0.73	14.66
4	MAILES	1,975	0	3	6	30	124	15	52	4	10.13	14.66
4	MrOS USA	2,002	0	1	0	0	2	0	0	40	2.15	14.66
4	SHIP	2,110	0	4	8	25	132	10	11	3	7.30	14.66
5	ARIC	1,556	0	0	0	2	2	1	56	4	4.05	14.47
5	BHS	2,021	0	64	62	**2,021**	343	100	760	23	46.21	14.47
5	CHS	1,123	0	1	4	2	0	1	17	0	2.23	14.47
5	EMAS	2,832	0	39	83	53	20	58	45	2	8.26	14.47
5	FHS	3,334	0	3	6	218	0	5	111	1	9.66	14.47
5	HIMS	4,121	0	16	4	4	0	2	8	0	0.73	14.47
5	MAILES	1,975	0	3	6	30	124	15	52	2	10.03	14.47
5	MrOS USA	2,002	0	1	0	0	2	0	0	0	0.15	14.47
5	SHIP	2,110	0	4	8	25	132	10	11	6	7.25	14.47
6	ARIC	1,556	0	0	0	2	2	1	56	**1,556**	3.86	5.63
6	BHS	2,021	0	64	62	**2,021**	343	100	760	**2,021**	46.21	5.63
6	CHS	1,123	0	1	4	2	0	1	17	1	2.23	5.63
6	EMAS	2,832	0	39	83	53	20	58	45	30	8.62	5.63
6	FHS	3,334	0	3	6	218	0	5	111	16	9.90	5.63
6	HIMS	4,121	0	16	4	4	0	2	8	2	0.73	5.63
6	MAILES	1,975	0	3	6	30	124	15	52	6	9.97	5.63

Model	Study	n	Age	BMI	Married/ De Facto	Higher Education	Alcohol	Smoking	Physical Activity	Health condition *j**	Incomplete cases (%)	
											Study**	IPDMA***
6	MrOS USA	2,002	0	1	0	0	2	0	0	1	0.20	5.63
6	SHIP	2,110	0	4	8	25	132	10	11	21	7.35	5.63
7	ARIC	1,556	0	0	0	2	2	1	56	0	3.86	15.13
7	BHS	2,021	0	64	62	**2,021**	343	100	760	0	46.21	15.13
7	CHS	1,123	0	1	4	2	0	1	17	**1,123**	2.23	15.13
7	EMAS	2,832	0	39	83	53	20	58	45	3	8.26	15.13
7	FHS	3,334	0	3	6	218	0	5	111	0	9.66	15.13
7	HIMS	4,121	0	16	4	4	0	2	8	0	0.73	15.13
7	MAILES	1,975	0	3	6	30	124	15	52	0	9.92	15.13
7	MrOS USA	2,002	0	1	0	0	2	0	0	0	0.15	15.13
7	SHIP	2,110	0	4	8	25	132	10	11	11	7.20	15.13
8	ARIC	1,556	0	0	0	2	2	1	56	2	3.98	14.48
8	BHS	2,021	0	64	62	**2,021**	343	100	760	0	46.21	14.48
8	CHS	1,123	0	1	4	2	0	1	17	0	2.23	14.48
8	EMAS	2,832	0	39	83	53	20	58	45	3	8.26	14.48
8	FHS	3,334	0	3	6	218	0	5	111	0	9.66	14.48
8	HIMS	4,121	0	16	4	4	0	2	8	0	0.73	14.48
8	MAILES	1,975	0	3	6	30	124	15	52	0	9.92	14.48
8	MrOS USA	2,002	0	1	0	0	2	0	0	0	0.15	14.48
8	SHIP	2,110	0	4	8	25	132	10	11	17	7.49	14.48
9	ARIC	1,556	0	0	0	2	2	1	56	0	3.86	18.92
9	BHS	2,021	0	64	62	**2,021**	343	100	760	0	46.21	18.92
9	CHS	1,123	0	1	4	2	0	1	17	96	9.88	18.92
9	EMAS	2,832	0	39	83	53	20	58	45	**2,832**	8.26	18.92
9	FHS	3,334	0	3	6	218	0	5	111	**3,334**	9.66	18.92
9	HIMS	4,121	0	16	4	4	0	2	8	3	0.73	18.92
9	MAILES	1,975	0	3	6	30	124	15	52	0	9.92	18.92

Model	Study	n	Age	BMI	Married/ De Facto	Higher Education	Alcohol	Smoking	Physical Activity	Health condition *j**	Incomplete cases (%)	
											**Study****	**IPDMA*****
9	MrOS USA	2,002	0	1	0	0	2	0	0	0	0.15	18.92
9	SHIP	2,110	0	4	8	25	132	10	11	**2,110**	7.16	18.92
10	ARIC	1,556	0	0	0	2	2	1	56	4	4.11	14.54
10	BHS	2,021	0	64	62	**2,021**	343	100	760	99	46.86	14.54
10	CHS	1,123	0	1	4	2	0	1	17	0	2.23	14.54
10	EMAS	2,832	0	39	83	53	20	58	45	19	8.79	14.54
10	FHS	3,334	0	3	6	218	0	5	111	4	9.78	14.54
10	HIMS	4,121	0	16	4	4	0	2	8	2	0.73	14.54
10	MAILES	1,975	0	3	6	30	124	15	52	2	9.92	14.54
10	MrOS USA	2,002	0	1	0	0	2	0	0	0	0.15	14.54
10	SHIP	2,110	0	4	8	25	132	10	11	8	7.16	14.54
11	ARIC	1,556	0	0	0	2	2	1	56	0	3.86	16.20
11	BHS	2,021	0	64	62	**2,021**	343	100	760	10	46.51	16.20
11	CHS	1,123	0	1	4	2	0	1	17	42	4.19	16.20
11	EMAS	2,832	0	39	83	53	20	58	45	31	9.15	16.20
11	FHS	3,334	0	3	6	218	0	5	111	6	9.81	16.20
11	HIMS	4,121	0	16	4	4	0	2	8	0	0.73	16.20
11	MAILES	1,975	0	3	6	30	124	15	52	3	10.08	16.20
11	MrOS USA	2,002	0	1	0	0	2	0	0	144	7.34	16.20
11	SHIP	2,110	0	4	8	25	132	10	11	195	15.36	16.20
12	ARIC	1,556	0	0	0	2	2	1	56	18	4.95	17.27
12	BHS	2,021	0	64	62	**2,021**	343	100	760	44	47.25	17.27
12	CHS	1,123	0	1	4	2	0	1	17	51	4.99	17.27
12	EMAS	2,832	0	39	83	53	20	58	45	99	11.37	17.27
12	FHS	3,334	0	3	6	218	0	5	111	78	11.64	17.27
12	HIMS	4,121	0	16	4	4	0	2	8	3	0.78	17.27
12	MAILES	1,975	0	3	6	30	124	15	52	95	14.08	17.27

Model	Study	n	Age	BMI	Married/ De Facto	Higher Education	Alcohol	Smoking	Physical Activity	Health condition *j**	Incomplete cases (%)	
											**Study****	**IPDMA*****
12	MrOS USA	2,002	0	1	0	0	2	0	0	144	7.34	17.27
12	SHIP	2,110	0	4	8	25	132	10	11	189	15.07	17.27
13	ARIC	1,556	0	0	0	2	2	1	56	0	3.86	16.15
13	BHS	2,021	0	64	62	**2,021**	343	100	760	10	46.51	16.15
13	CHS	1,123	0	1	4	2	0	1	17	40	4.01	16.15
13	EMAS	2,832	0	39	83	53	20	58	45	27	9.00	16.15
13	FHS	3,334	0	3	6	218	0	5	111	6	9.81	16.15
13	HIMS	4,121	0	16	4	4	0	2	8	0	0.73	16.15
13	MAILES	1,975	0	3	6	30	124	15	52	3	10.08	16.15
13	MrOS USA	2,002	0	1	0	0	2	0	0	144	7.34	16.15
13	SHIP	2,110	0	4	8	25	132	10	11	190	15.12	16.15
14	ARIC	1,556	0	0	0	2	2	1	56	0	3.86	18.77
14	BHS	2,021	0	64	62	**2,021**	343	100	760	6	46.36	18.77
14	CHS	1,123	0	1	4	2	0	1	17	55	5.34	18.77
14	EMAS	2,832	0	39	83	53	20	58	45	20	8.83	18.77
14	FHS	3,334	0	3	6	218	0	5	111	0	9.66	18.77
14	HIMS	4,121	0	16	4	4	0	2	8	1	0.75	18.77
14	MAILES	1,975	0	3	6	30	124	15	52	561	37.92	18.77
14	MrOS USA	2,002	0	1	0	0	2	0	0	144	7.34	18.77
14	SHIP	2,110	0	4	8	25	132	10	11	187	14.93	18.77
15	ARIC	1,556	0	0	0	2	2	1	56	0	3.86	18.84
15	BHS	2,021	0	64	62	**2,021**	343	100	760	74	46.21	18.84
15	CHS	1,123	0	1	4	2	0	1	17	2	2.40	18.84
15	EMAS	2,832	0	39	83	53	20	58	45	897	37.46	18.84
15	FHS	3,334	0	3	6	218	0	5	111	3	9.72	18.84
15	HIMS	4,121	0	16	4	4	0	2	8	0	0.73	18.84
15	MAILES	1,975	0	3	6	30	124	15	52	0	9.92	18.84

Model	Study	n	Age	BMI	Married/ De Facto	Higher Education	Alcohol	Smoking	Physical Activity	Health condition *j**	Incomplete cases (%)	
											**Study****	**IPDMA*****
15	MrOS USA	2,002	0	1	0	0	2	0	0	99	5.04	18.84
15	SHIP	2,110	0	4	8	25	132	10	11	0	7.16	18.84
16	ARIC	1,556	0	0	0	2	2	1	56	0	3.86	10.83
16	BHS	2,021	0	64	62	**2,021**	343	100	760	**2,021**	46.21	10.83
16	CHS	1,123	0	1	4	2	0	1	17	0	2.23	10.83
16	EMAS	2,832	0	39	83	53	20	58	45	897	37.46	10.83
16	FHS	3,334	0	3	6	218	0	5	111	2	9.69	10.83
16	HIMS	4,121	0	16	4	4	0	2	8	4	0.73	10.83
16	MAILES	1,975	0	3	6	30	124	15	52	0	9.92	10.83
16	MrOS USA	2,002	0	1	0	0	2	0	0	**2,002**	0.15	10.83
16	SHIP	2,110	0	4	8	25	132	10	11	0	7.16	10.83

* Health conditions, from j = 1-14: Diastolic BP, Systolic BP, Hypertension, General health, prevalent CVD, prevalent cancer, COPD, Diabetes, Cholesterol / HDL, LDL, HDL, Creatinine level, Lipid lowering medications, Psychotropic drug use.

** Percentage of participants with incomplete cases (missing values for at least one variable), excluding those variables that were entirely missing (i.e., no observations) from a dataset (bold, shaded cells = systematic missingness).

*** Percentages of incomplete cases for combined IPD across studies (‘IPDMA’). The percentages of incomplete cases in this column are calculated using the merged datasets, prior to imputation and model fitting.

Supplementary Figures

Figure S1. Flow chart identifying studies selected from systematic review and the IPD and AD obtained from those studies.

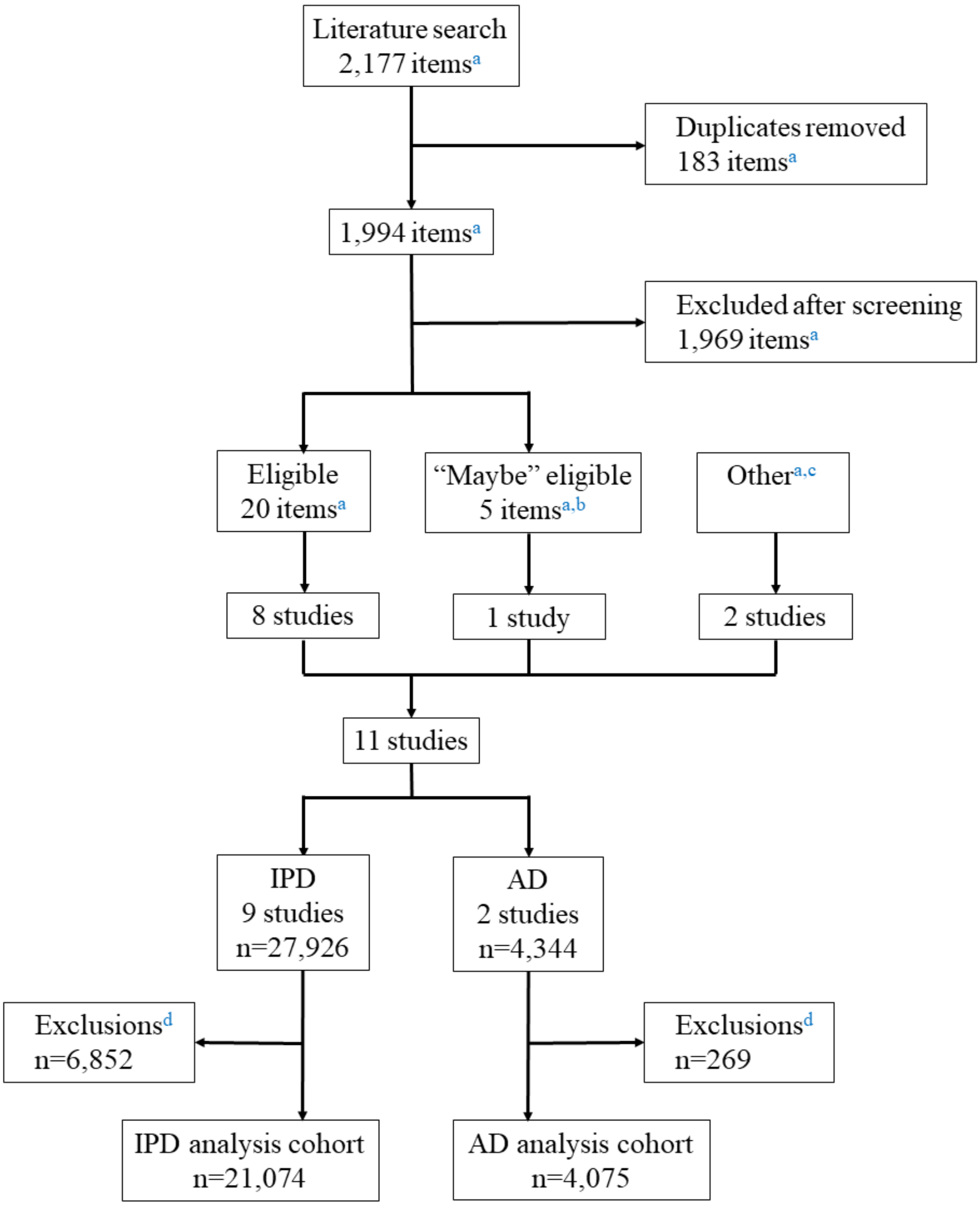

a. Further details on systematic literature searches, screening and selection of items (journal articles, reports, theses, webpage articles), with a PRISMA flow chart for the systematic review are reported in Marriott et al.[1]
b. Items identified as "Maybe" at completion of systematic screening were further investigated using information external to the systematic review, resulting in the identification of one additional eligible study with IPD-level data.
c. Additional studies identified through known contacts of the authors in Marriott et al.[1]
d. For analyses of testosterone associations, data were excluded for participants with missing measurements of total testosterone made using mass spectrometry at baseline, who had history of orchidectomy, or were using androgen or anti-androgen medications.

Figure S2. Summary curves and forest plots for the associations of sociodemographic factors with DHT and Estradiol concentrations. Presented estimates obtained from models controlling for all other sociodemographic factors in Model 1 (refer Appendix Table A1). MD = mean difference; vertical dashed line on summary curves identifies the reference level (ref.) for the predictor of interest; dotted lines show 95% prediction intervals; forest plots show the MD from the reference level of the categorical predictor (refer Table S2, S3).

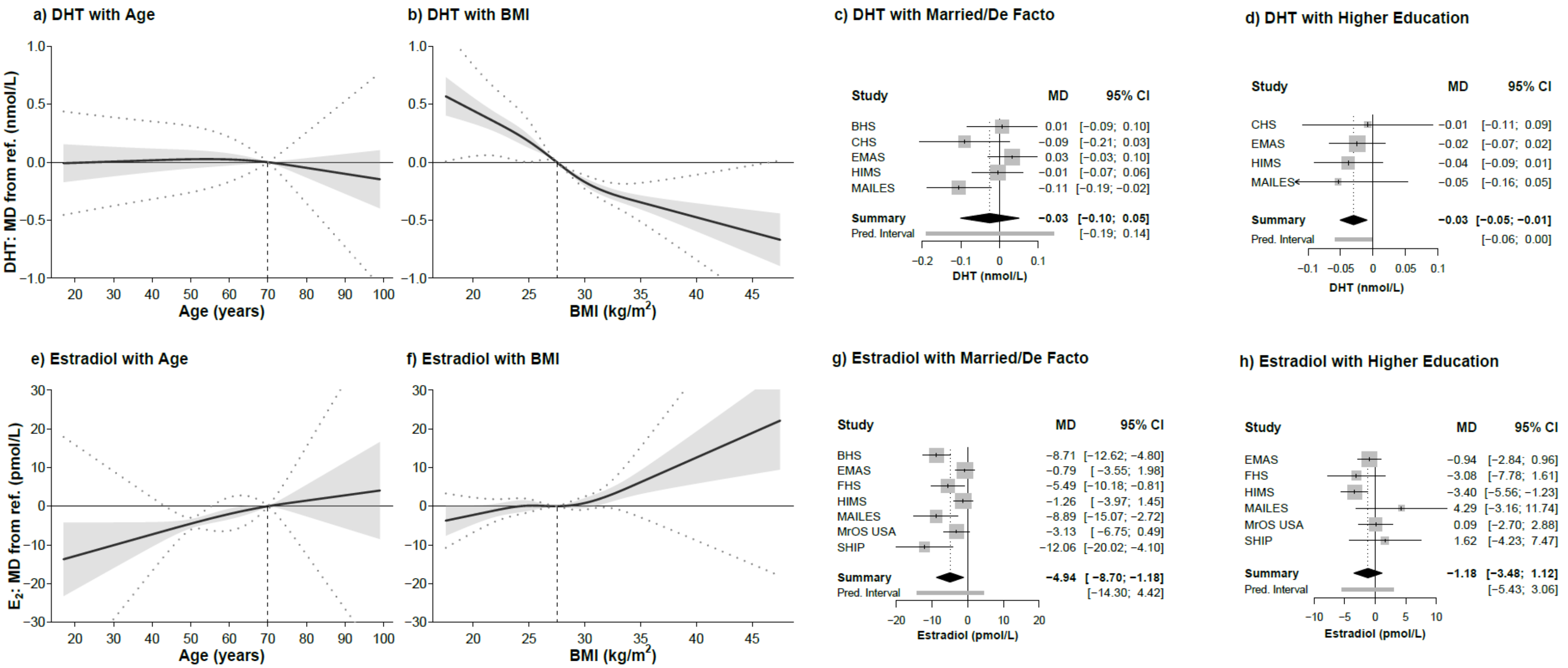

Figure S3. Estimated associations of hormone variables with age and BMI by study from the first stage of the IPDMA after controlling for all other sociodemographic predictors in Model 1 (refer Appendix Table A1).

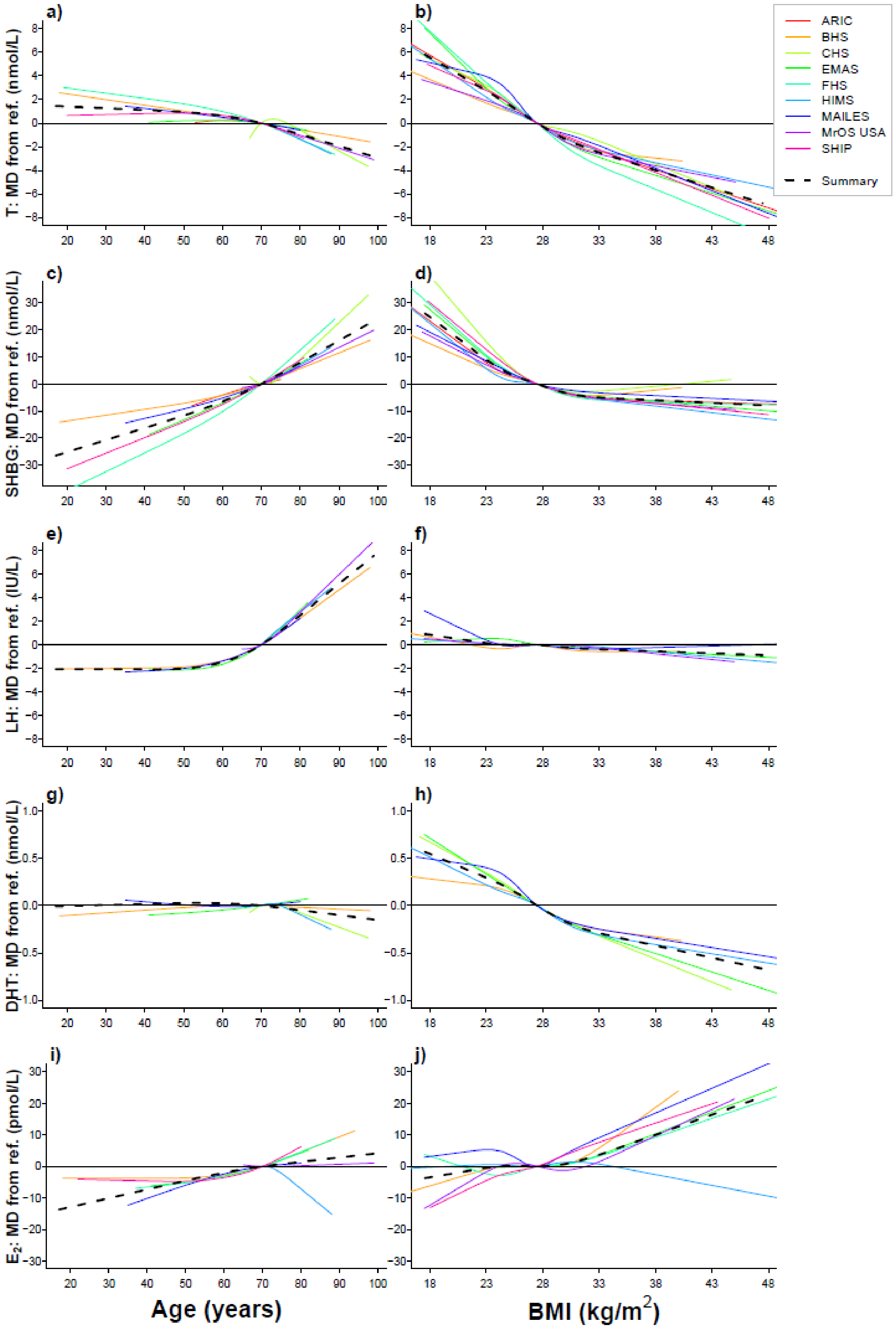

Figure S4. Forest plots for the associations of lifestyle factors with testosterone, SHBG, and LH concentrations. MD = mean difference; forest plots show the MD from the reference level of the categorical predictor (refer Supplementary Tables S2, S3). Presented estimates obtained from models controlling for all sociodemographic and other lifestyle factors in Model 2 (refer Appendix Table A1).

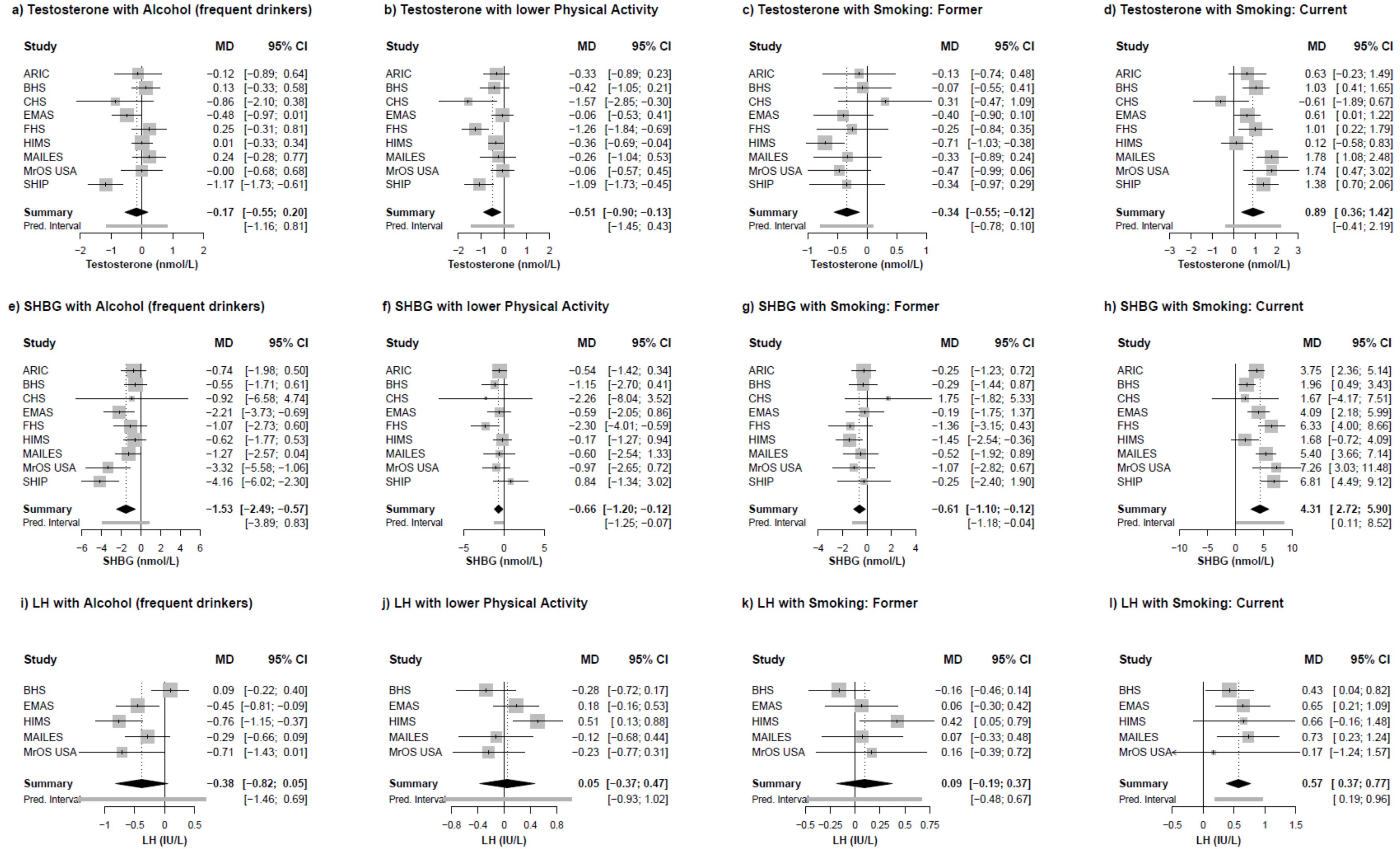

Figure S5. Forest plots for the associations of lifestyle factors with DHT and Estradiol concentrations. MD = mean difference; forest plots show the MD from the reference level of the categorical predictor (refer Table S2, S3). Presented estimates obtained from models controlling for all sociodemographic and other lifestyle factors in Model 2 (refer Appendix Table A1).

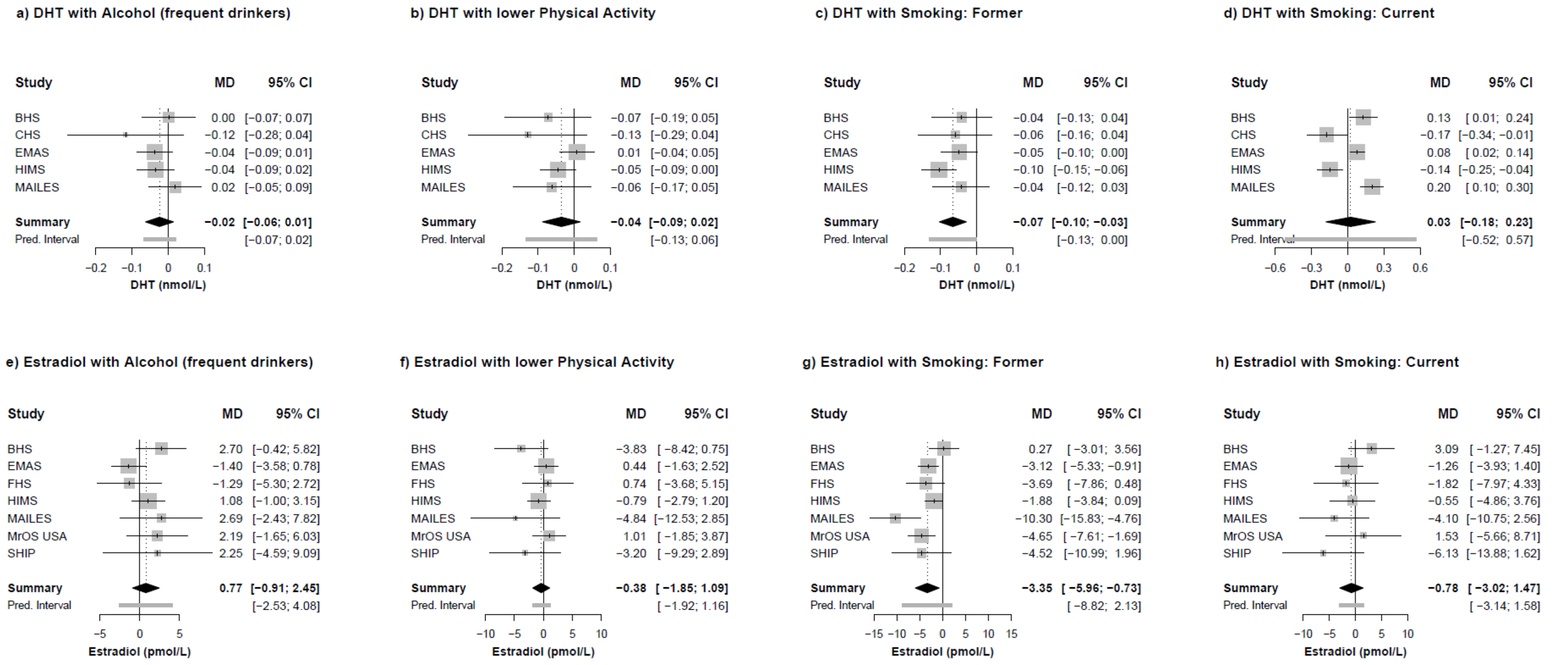

Figure S6. Summary curves and forest plots for the associations of prevalent health conditions with SHBG concentration. MD = mean difference; vertical dashed line on summary curves identifies the reference level (ref.) for the predictor of interest; dotted lines show 95% prediction intervals; forest plots show the MD from the reference level of the categorical predictor (refer Table S2, S3). Presented estimates obtained from models controlling for all sociodemographic and lifestyle factors (refer Appendix Table A1).

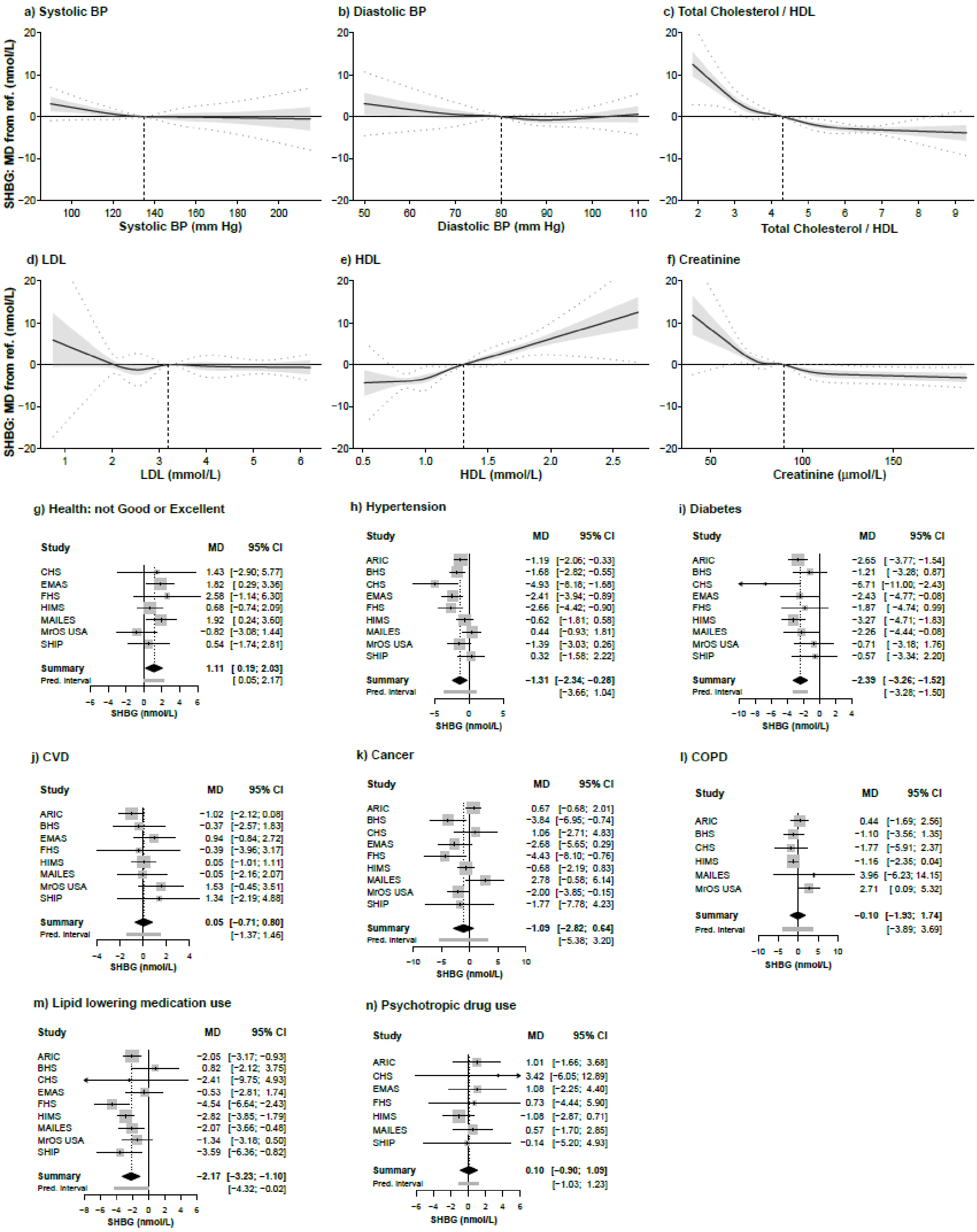

Figure S7. Summary curves and forest plots for the associations of prevalent health conditions with LH concentration. MD = mean difference; vertical dashed line on summary curves identifies the reference level (ref.) for the predictor of interest; dotted lines show 95% prediction intervals; forest plots show the MD from the reference level of the categorical predictor (refer Table S2, S3). Presented estimates obtained from models controlling for all sociodemographic and lifestyle factors (refer Appendix Table A1).

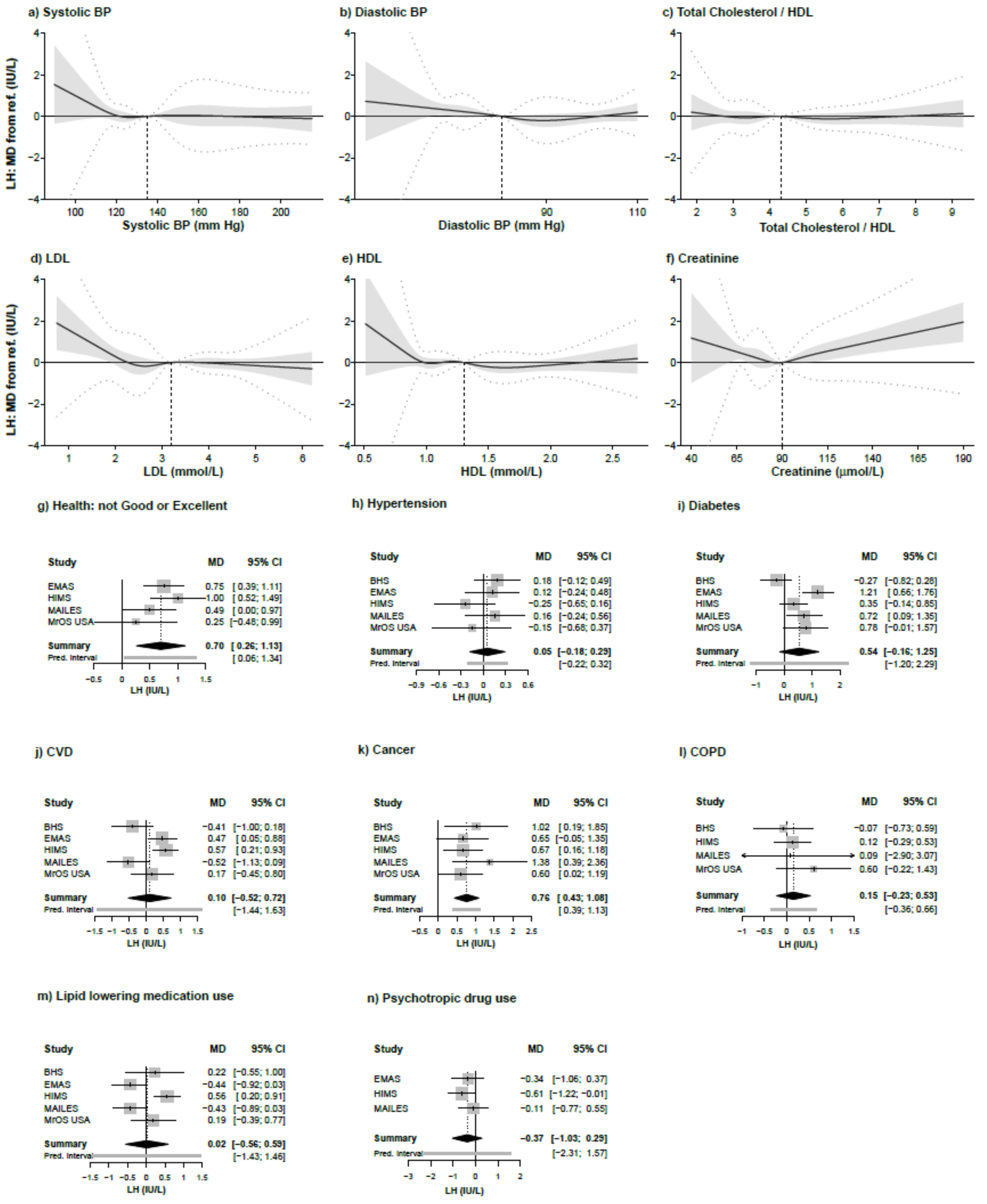

Figure S8. Summary curves and forest plots for the associations of prevalent health conditions with DHT concentration. MD = mean difference; vertical dashed line on summary curves identifies the reference level (ref.) for the predictor of interest; dotted lines show 95% prediction intervals; forest plots show the MD from the reference level of the categorical predictor (refer Table S2, S3). Presented estimates obtained from models controlling for all sociodemographic and lifestyle factors (refer Appendix Table A1).

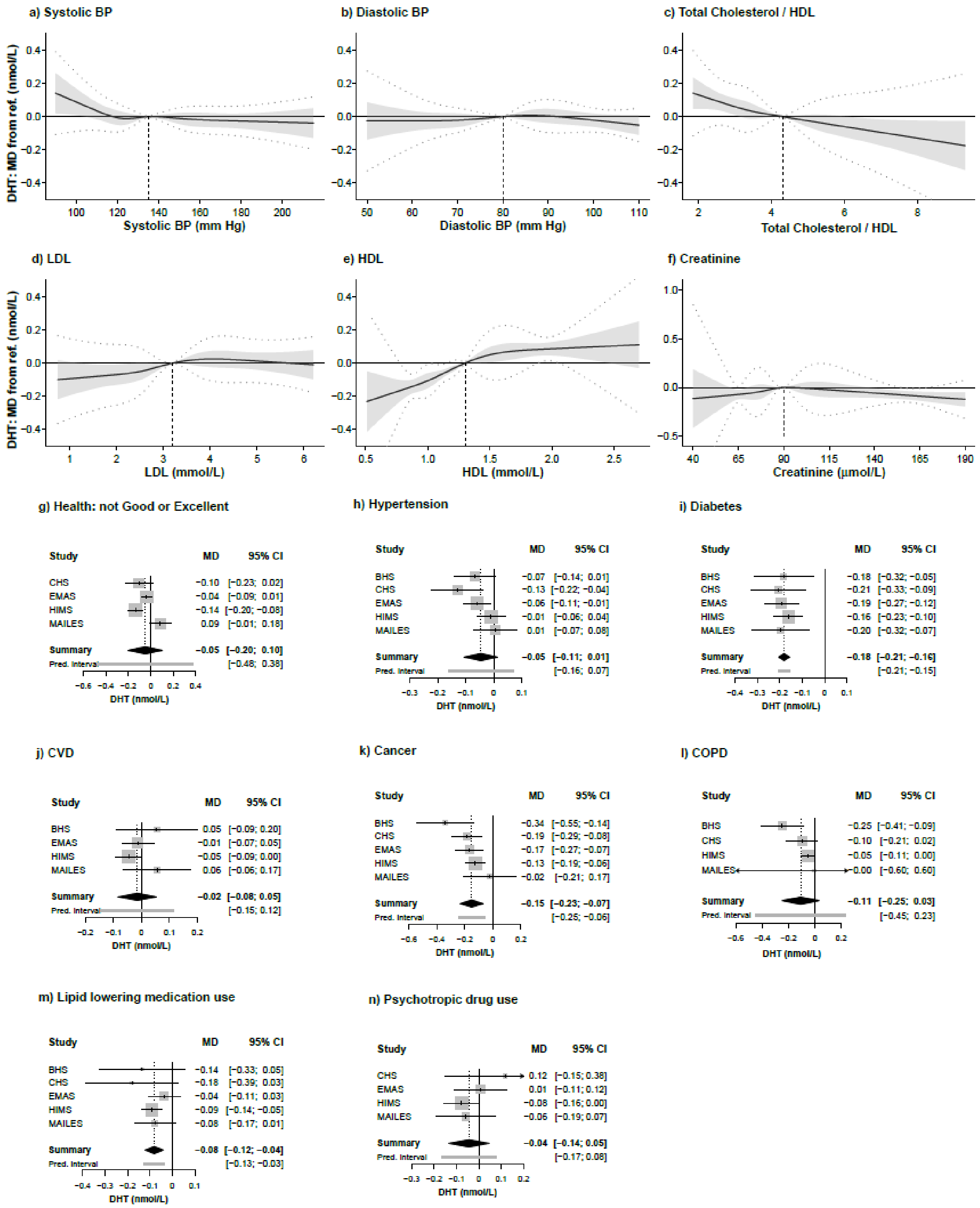

Figure S9. Summary curves and forest plots for the associations of prevalent health conditions with estradiol concentration. MD = mean difference; vertical dashed line on summary curves identifies the reference level (ref.) for the predictor of interest; dotted lines show 95% prediction intervals; forest plots show the MD from the reference level of the categorical predictor (refer Table S2, S3). Presented estimates obtained from models controlling for all sociodemographic and lifestyle factors (refer Appendix Table A1).

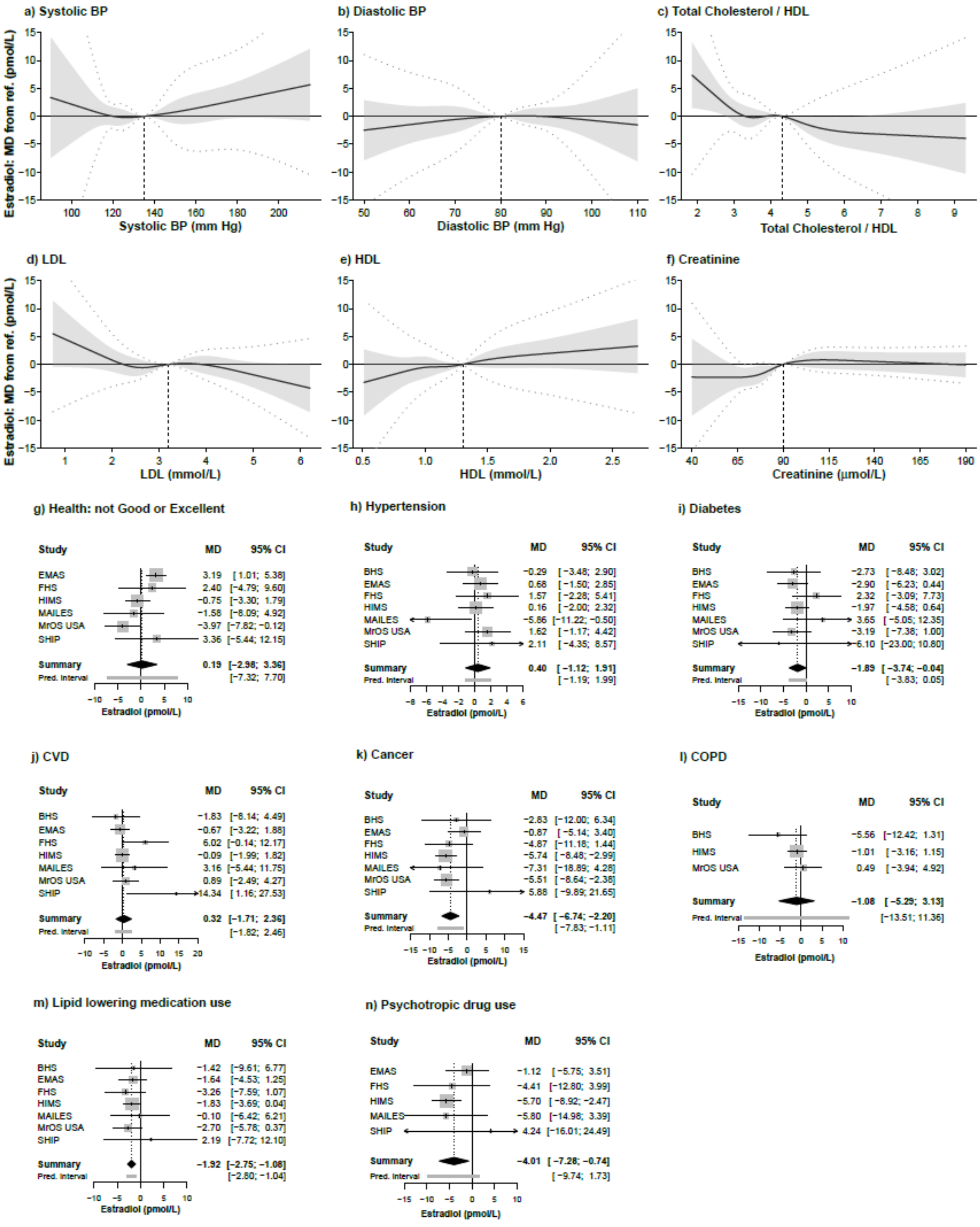

Figure S10. Associations of LH with creatinine among studies providing IPD for both variables (I^2 = 72.3, CI 55.0, 83.0). Presented estimates obtained from models controlling for all sociodemographic and lifestyle factors (refer Appendix Table A1).

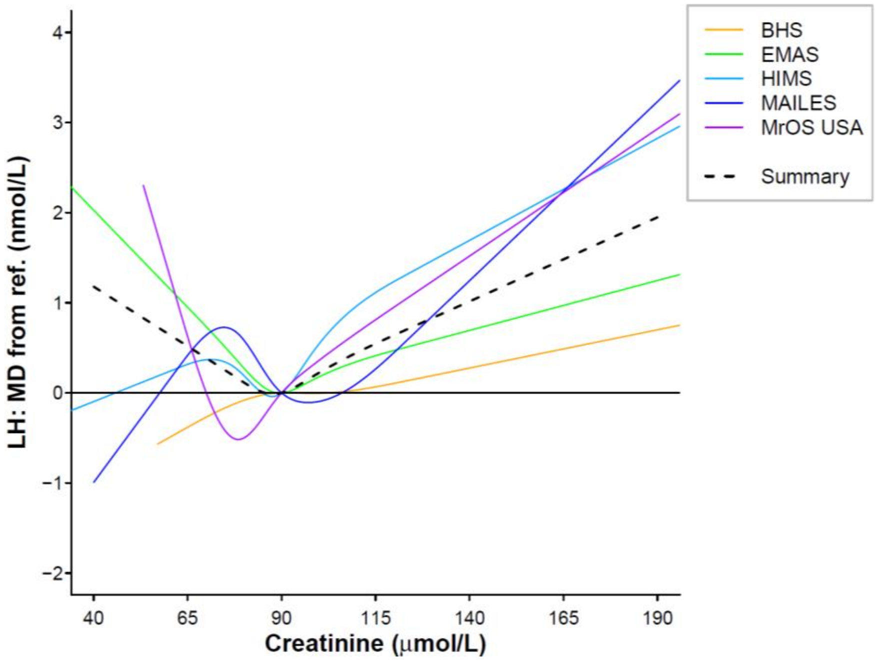

Figure S11. Comparison of summary curves for the association of hormone variables with age when modelled using a restricted cubic spline versus a cubic polynomial function. Presented estimates obtained from models controlling for all other sociodemographic factors in Model 1 (refer Appendix Table A1).

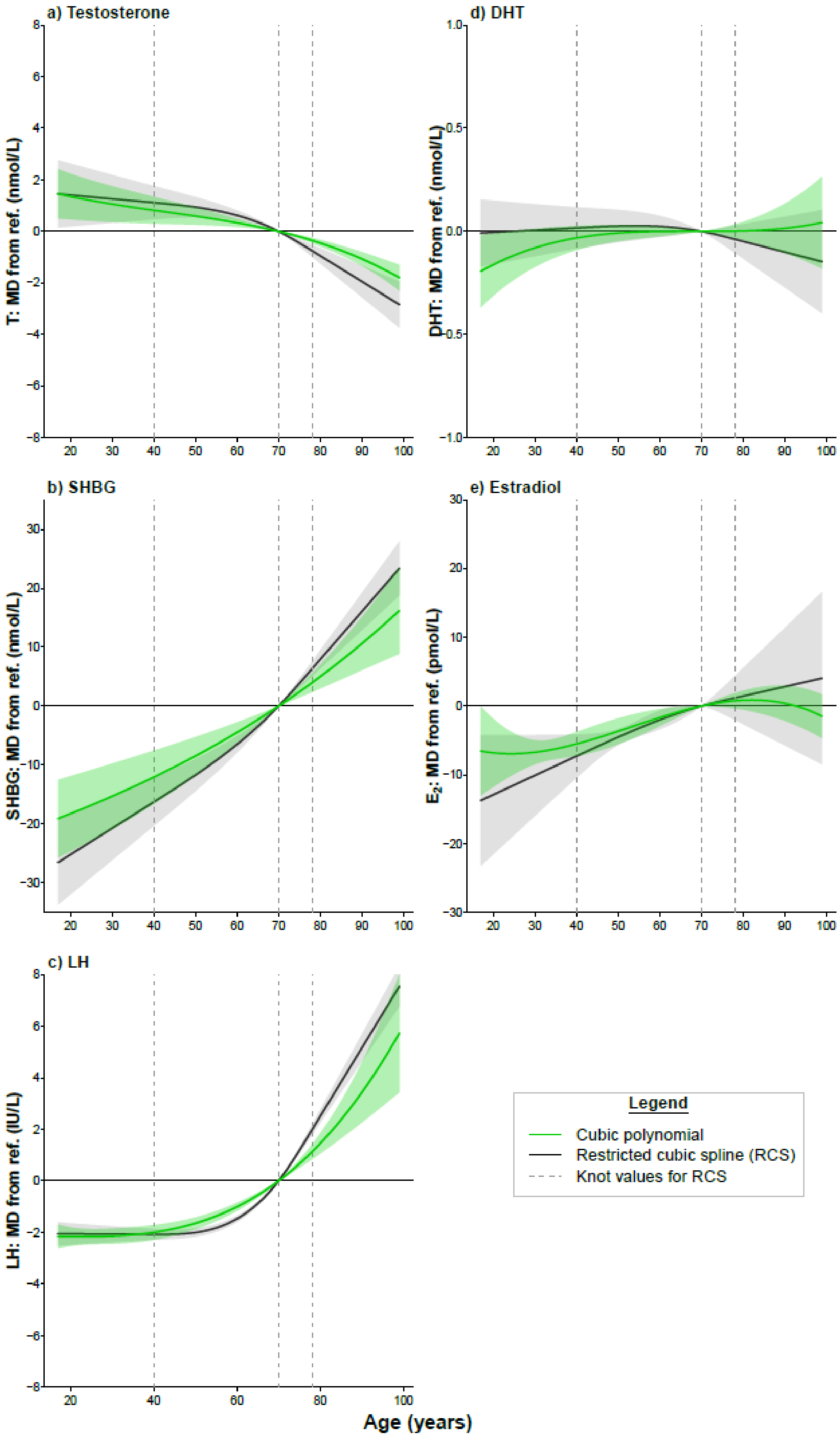

Figure S12. Comparison of summary estimates for studies with data on baseline testosterone concentration and the predictor of interest (excluding studies with missing data) versus estimates from all studies that provided IPD-level data, which had the missing variable systematically imputed. MD = mean difference from the reference level or value (refer Tables S2, S3).

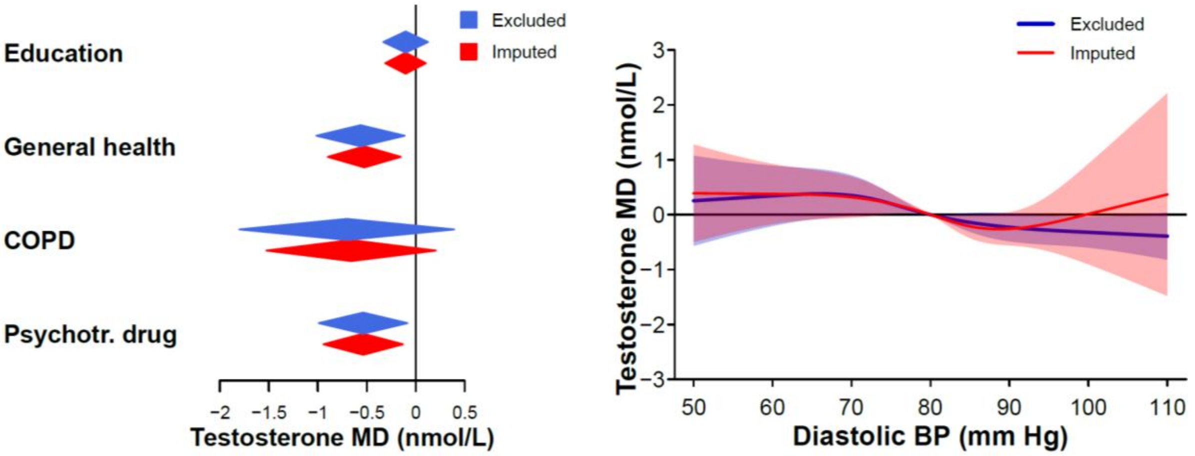

Model	Predictor	Level	Excluded	Imputed
1	Higher Education	Yes	-0.10 (-0.33- 0.13)	-0.11 (-0.31- 0.10)
3	Diastolic BP[a]		-0.40 (-0.72--0.08)	-0.47 (-0.81--0.13)
6	General health	<Good[b]	-0.56 (-1.02--0.11)	-0.53 (-0.90--0.15)
9	COPD	Yes	-0.70 (-1.80- 0.39)	-0.66 (-1.53- 0.21)
16	Psychotr. drug	Yes	-0.54 (-0.99--0.08)	-0.54 (-0.94--0.13)

a. Effect sizes presented as change for 1 standard deviation increase around the Ref. value; Ref. values listed in Supplementary Table S3 (summary of harmonised variables).
b. <Good = Fair, Poor or Very Poor.

Figure S13. Comparison of summary estimates for studies with data on baseline SHBG concentration and the predictor of interest (excluding studies with missing data) versus estimates from all studies that provided IPD-level data, which had the missing variable systematically imputed. MD = mean difference from the reference level or value (refer Tables S2, S3).

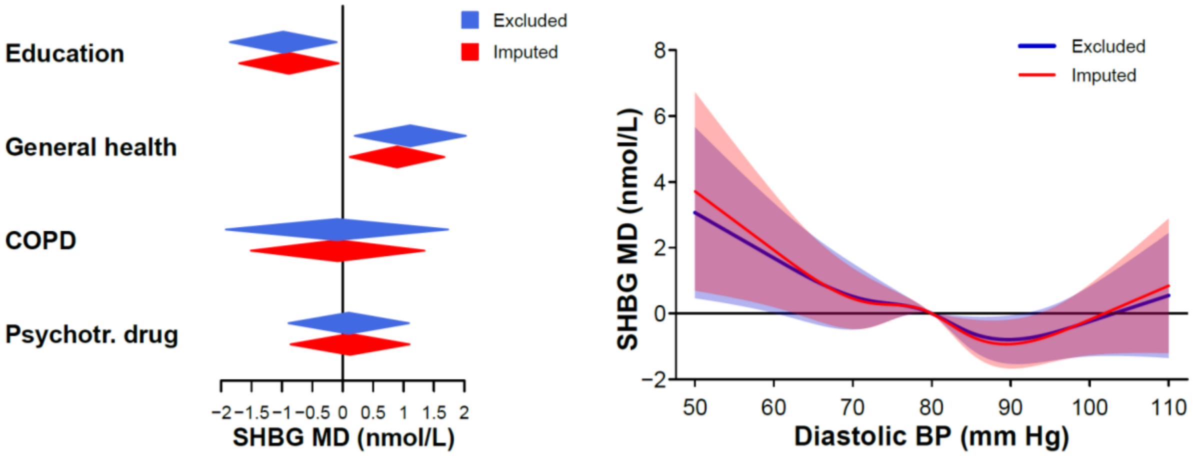

Model	Predictor	Level	Excluded	Imputed
1	Higher Education	Yes	-0.98 (-1.86--0.10)	-0.89 (-1.71--0.07)
3	Diastolic BP[a]		-0.99 (-1.86--0.12)	-1.07 (-1.93--0.20)
6	General health	<Good[b]	1.11 (0.19- 2.03)	0.89 (0.11- 1.68)
9	COPD	Yes	-0.10 (-1.93- 1.74)	-0.08 (-1.52- 1.35)
16	Psychotr. drug	Yes	0.10 (-0.90- 1.09)	0.12 (-0.86- 1.10)

a. Effect sizes presented as change for 1 standard deviation increase around the Ref. value; Ref. values listed in Supplementary Table S3 (summary of harmonised variables).
b. <Good = Fair, Poor or Very Poor.

Figure S14. Comparison of summary estimates for studies with data on baseline LH concentration and the predictor of interest (excluding studies with missing data) versus estimates from all studies that provided IPD-level data, which had the missing variable systematically imputed. MD = mean difference from the reference level or value (refer Tables S2, S3).

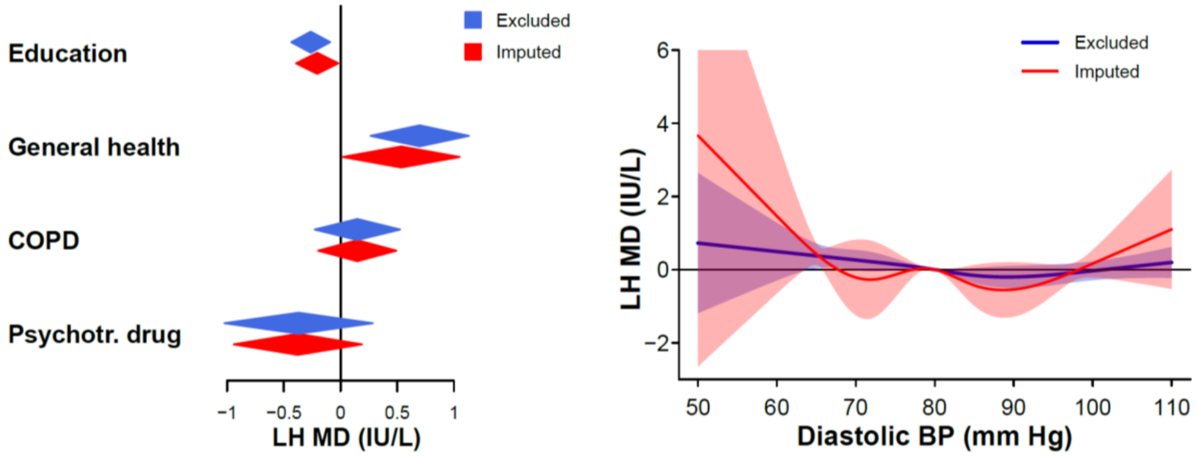

Model	Predictor	Level	Excluded	Imputed
1	Higher Education	Yes	-0.26 (-0.43--0.09)	-0.21 (-0.39--0.02)
3	Diastolic BP[a]		-0.35 (-0.55--0.14)	-0.29 (-0.55--0.04)
6	General health	<Good[b]	0.70 (0.26- 1.13)	0.53 (0.02- 1.05)
9	COPD	Yes	0.15 (-0.23- 0.53)	0.15 (-0.20- 0.49)
16	Psychotr. drug	Yes	-0.37 (-1.03- 0.29)	-0.38 (-0.94- 0.19)

a. Effect sizes presented as change for 1 standard deviation increase around the Ref. value; Ref. values listed in Supplementary Table S3 (summary of harmonised variables).
b. <Good = Fair, Poor or Very Poor.

Figure S15. Comparison of summary estimates for studies with data on baseline DHT concentration and the predictor of interest (excluding studies with missing data) versus estimates from all studies that provided IPD-level data, which had the missing variable systematically imputed. MD = mean difference from the reference level or value (refer Tables S2, S3). A comparison of estimates was not required for the association with diastolic blood pressure because either DHT and diastolic blood pressure were available or neither were available, and in the latter cases systematic imputations were not done.

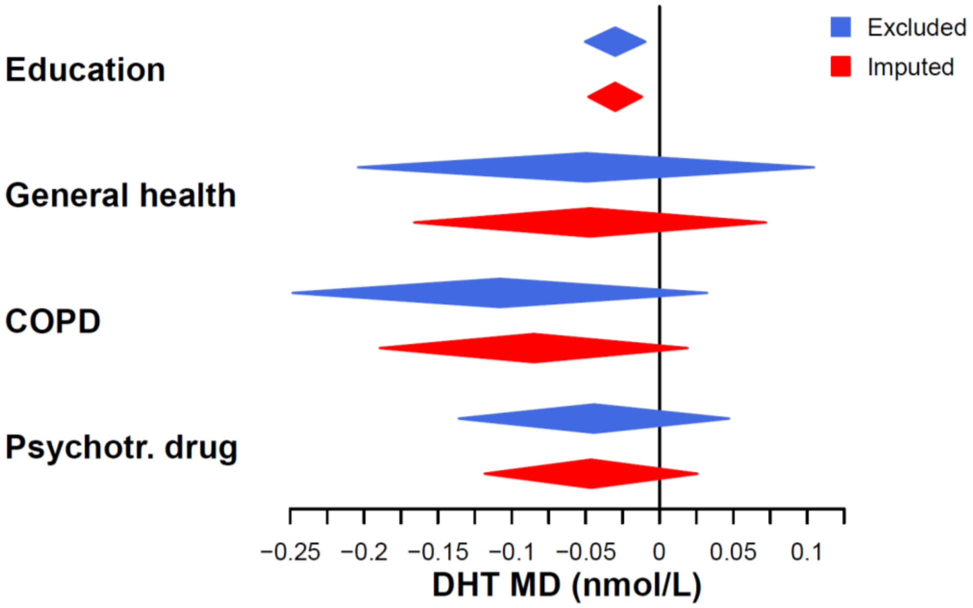

Model	Predictor	Level	Excluded	Imputed
1	Higher Education	Yes	-0.030 (-0.051--0.009)	-0.030 (-0.048--0.012)
6	General health	<Good[a]	-0.050 (-0.204- 0.105)	-0.047 (-0.166- 0.072)
9	COPD	Yes	-0.108 (-0.249- 0.032)	-0.085 (-0.190- 0.019)
16	Psychotr. drug	Yes	-0.044 (-0.136- 0.047)	-0.046 (-0.119- 0.026)

a. <Good = Fair, Poor or Very Poor.

Figure S16. Comparison of summary estimates for studies with data on baseline estradiol concentration and the predictor of interest (excluding studies with missing data) versus estimates from all studies that provided IPD-level data, which had the missing variable systematically imputed. MD = mean difference from the reference level or value (refer Tables S2, S3).

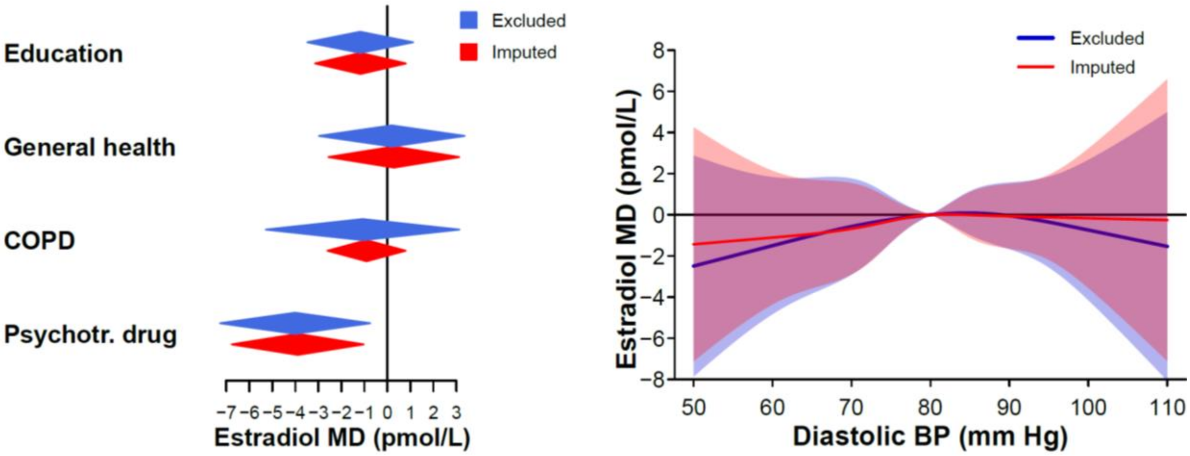

Model	Predictor	Level	Excluded	Imputed
1	Higher Education	Yes	-1.18 (-3.48- 1.12)	-1.17 (-3.14- 0.81)
3	Diastolic BP[a]		0.36 (-1.34- 2.07)	0.35 (-1.27- 1.97)
6	General health	<Good[b]	0.19 (-2.98- 3.36)	0.28 (-2.57- 3.13)
9	COPD	Yes	-1.08 (-5.29- 3.13)	-0.91 (-2.61- 0.80)
16	Psychotr. drug	Yes	-4.01 (-7.28--0.74)	-3.90 (-6.77--1.03)

a. Effect sizes presented as change for 1 standard deviation increase around the Ref. value; Ref. values listed in Supplementary Table S3 (summary of harmonised variables).
b. <Good = Fair, Poor or Very Poor.

Figure S17. Sensitivity of summary curves to the inclusion of ethnicity types other than "White". Plots compare summary curves for the associations of total testosterone (T) with participant age (a, b) and BMI (c, d). Comparisons are made: (i) from IPD where the ethnicity type was supplied as a variable (a, c); (ii) of subsets of IPD where participants are classified as being White on the basis of recorded ethnicity and other information (b, d). MD = mean difference from the reference value (refer Tables S2, S3).

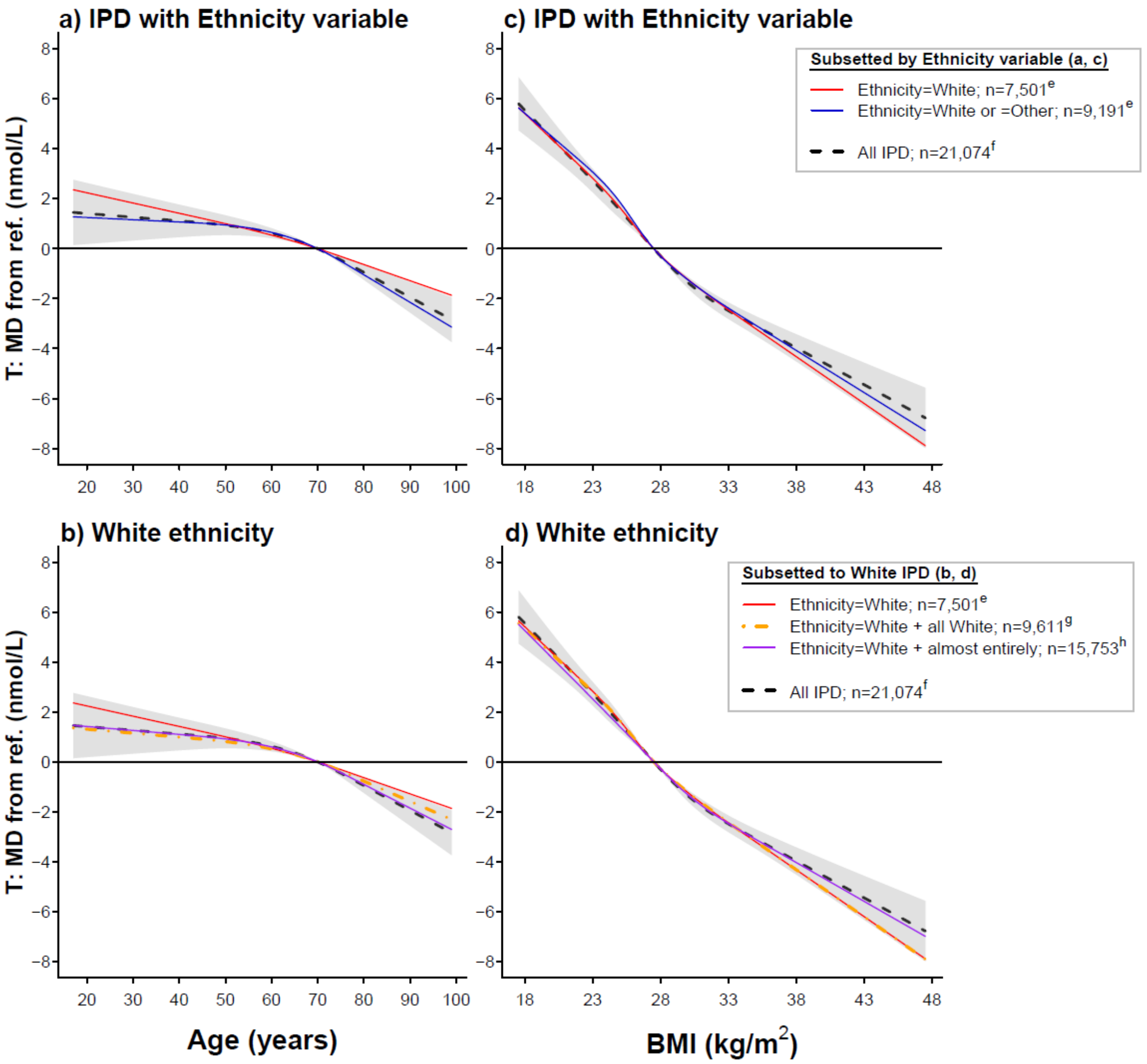

e. IPD with Ethnicity variable supplied: ARIC, CHS, FHS, MAILES (FAMAS Wave 1 cohort), MrOS USA.
f. All IPD, as included in the main analysis, with no subsetting based on ethnicity type: ARIC, BHS, CHS, EMAS, FHS, HIMS, MAILES, MrOS USA, SHIP.
g. IPD with Ethnicity variable supplied which has been subsetted to White participants only, and from one additional study comprised entirely of White participants: ARIC, CHS, FHS, MAILES (FAMAS Wave 1 cohort), MrOS USA, SHIP.
h. IPD with Ethnicity variable supplied which has been subsetted to White participants only, and from additional studies comprised entirely, or almost entirely, of White participants: ARIC, BHS, CHS, FHS, HIMS, MAILES (FAMAS Wave 1 cohort), MrOS USA, SHIP.

Figure S18. Sensitivity of summary estimates to the inclusion of ethnicity types other than "White". Summary estimates show the mean difference from the reference level of the categorical predictor for different subsets of IPD, based on ethnicity type. Vertical grey lines represent the 95% confidence interval for the summary estimate calculated from all IPD, as included in the main analysis, which had no sub-setting based on ethnicity type.

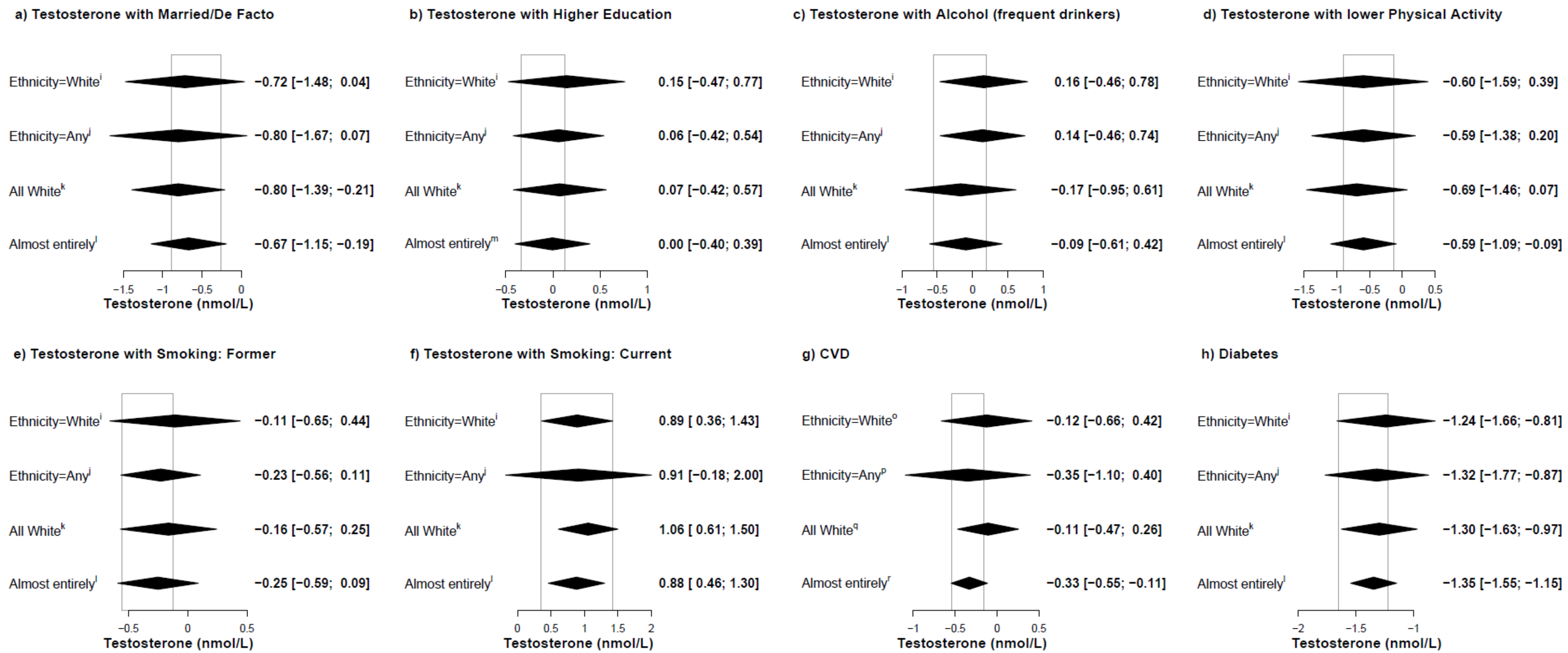

i. 'Ethnicity=White'. IPD with Ethnicity variable supplied, subsetted to White participants only: ARIC, CHS, FHS, MAILES (FAMAS Wave 1 cohort), MrOS USA; n=7,501.
j. 'Ethnicity=Any'. IPD with Ethnicity variable supplied, not subsetted: ARIC, CHS, FHS, MAILES (FAMAS Wave 1 cohort), MrOS USA; n=9,191.

k. ‘All White’. IPD with Ethnicity variable supplied which has been subsetted to White participants only, and from one additional study comprised entirely of White participants: ARIC, CHS, FHS, MAILES (FAMAS Wave 1 cohort), MrOS USA, SHIP; n=9,611.
l. ‘Almost entirely’ White. IPD with Ethnicity variable supplied which has been subsetted to White participants only, and from additional studies comprised entirely, or almost entirely, of White participants: ARIC, BHS, CHS, FHS, HIMS, MAILES (FAMAS Wave 1 cohort), MrOS USA, SHIP; n=15,753.
m. ‘Almost entirely’ White. IPD with Ethnicity variable supplied which has been subsetted to White participants only, and from additional studies comprised entirely, or almost entirely, of White participants: ARIC, CHS, FHS, HIMS, MAILES (FAMAS Wave 1 cohort), MrOS USA, SHIP. BHS did not have an education variable so was omitted from this sensitivity analysis; n=13,732.
o. ‘Ethnicity=White’. IPD with Ethnicity variable supplied, subsetted to White participants only: ARIC, FHS, MAILES (FAMAS Wave 1 cohort), MrOS USA; n=6,548. CHS data omitted because participants with history of CVD were excluded in that study.
p. ‘Ethnicity=Any’. IPD with Ethnicity variable supplied, not subsetted: ARIC, FHS, MAILES (FAMAS Wave 1 cohort), MrOS USA; n=8,068. CHS data omitted because participants with history of CVD were excluded in that study.
q. ‘All White’. IPD with Ethnicity variable supplied which has been subsetted to White participants only, and from one additional study comprised entirely of White participants: ARIC, FHS, MAILES (FAMAS Wave 1 cohort), MrOS USA, SHIP; n=8,658. CHS data omitted because participants with history of CVD were excluded in that study.
r. ‘Almost entirely’ White. IPD with Ethnicity variable supplied which has been subsetted to White participants only, and from additional studies comprised entirely, or almost entirely, of White participants: ARIC, BHS, FHS, HIMS, MAILES (FAMAS Wave 1 cohort), MrOS USA, SHIP; n=14,800. CHS data omitted because participants with history of CVD were excluded in that study.

Figure S19. Contour-enhanced funnel plots. Estimates from studies with individual participant data are presented as solid (black) dots and without as white dots. Contours show regions of the probability of a mean difference (MD) not different from zero. Egger's tests of symmetry were done for plots of at least 10 studies, with P<0.1 taken to indicate evidence of asymmetry. * = Funnel plots show the effect size as the change in testosterone from an increase in 1 SD of: (a) age around the reference age of 70 years; (b) BMI around the reference mean of 27.5 kg/m^2. Summary estimate from the analysis of studies with IPD only is shown as the vertical dashed line (brown). This figure is continued onto the next page.

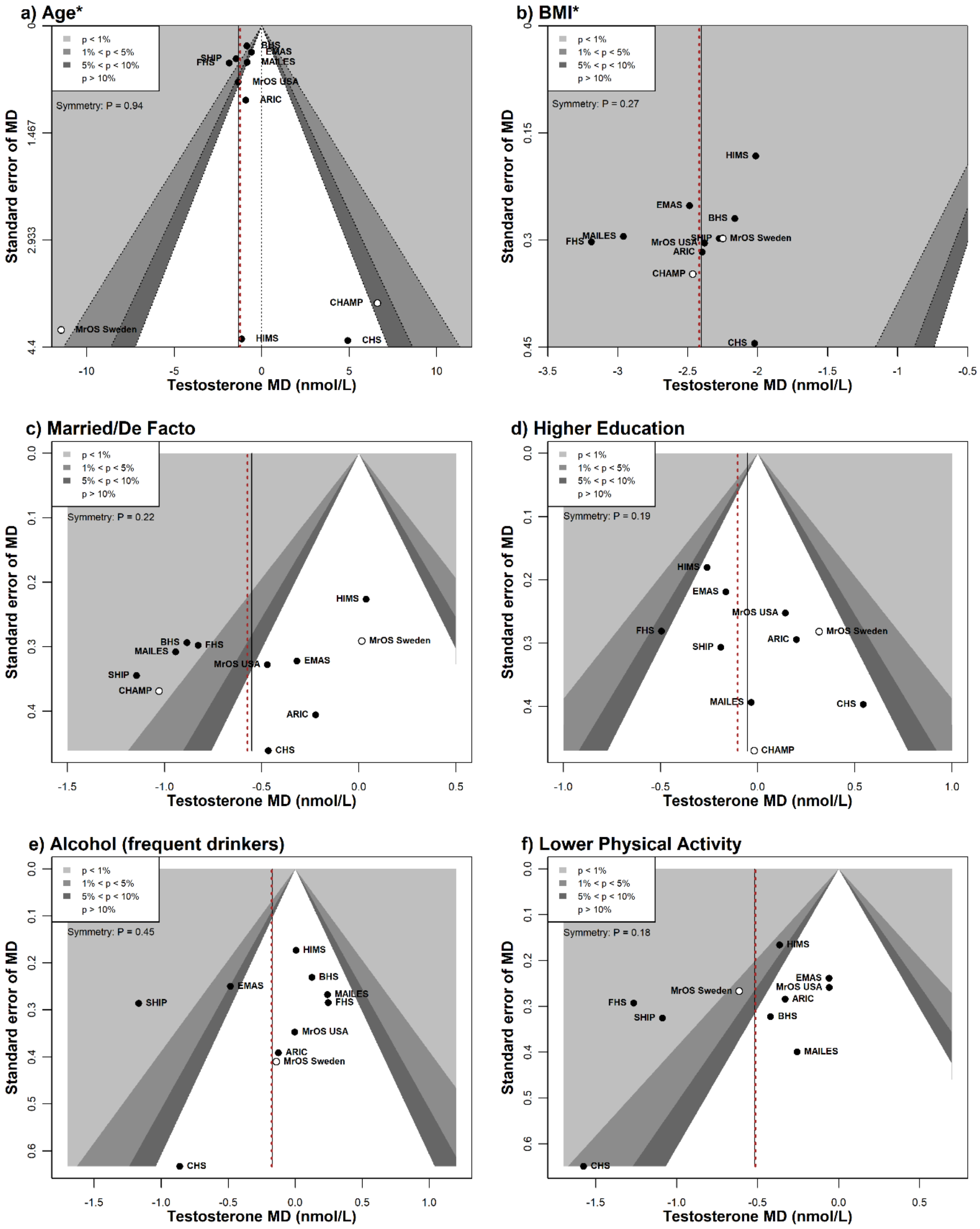

Figure S19 (continued from previous page).

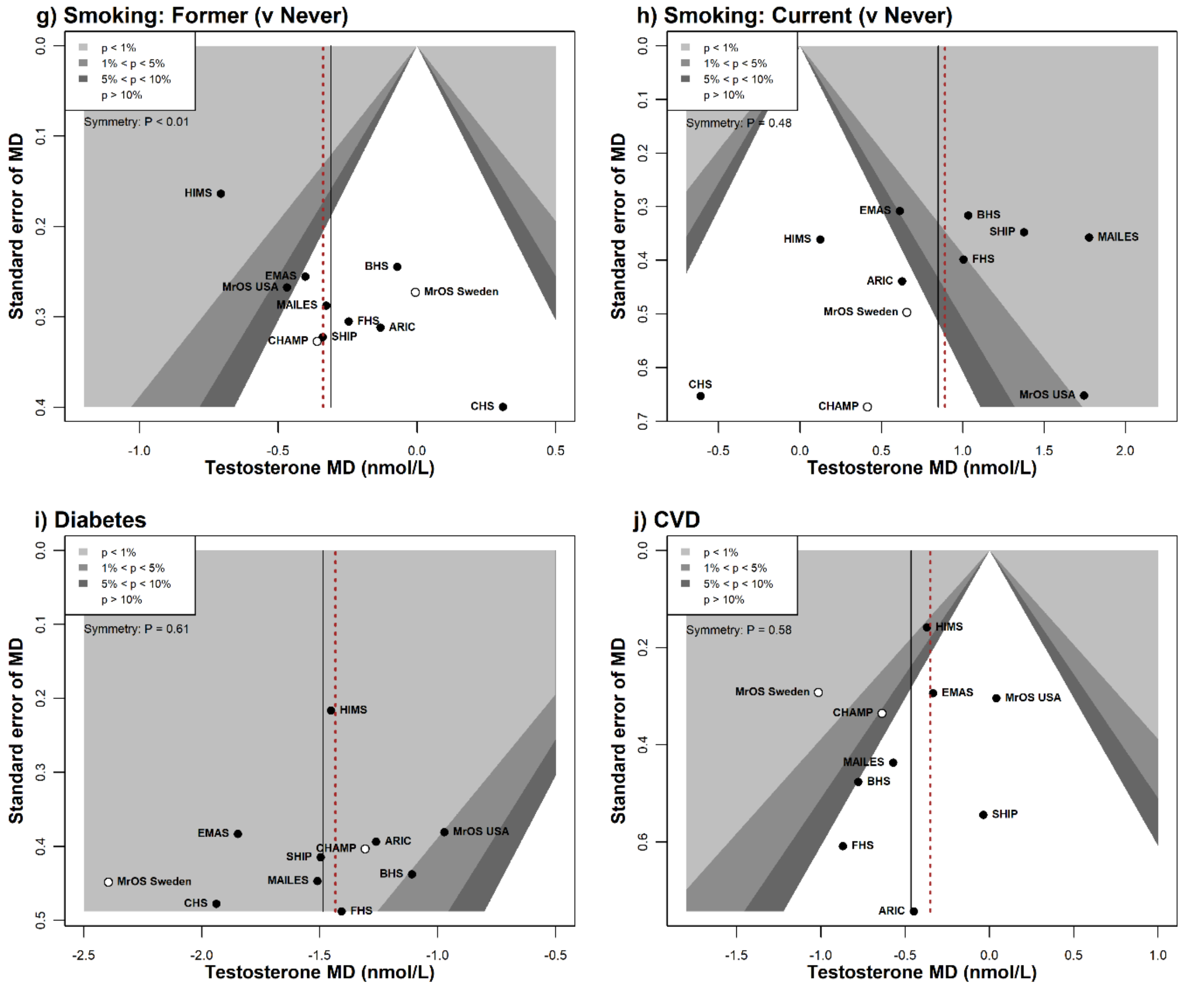

Figure S20. Summary curves for the associations of (a) baseline testosterone concentration (nmol/L) with baseline age and (b) baseline LH concentration (IU/L) with age, for all IPD versus IPD for men who were free of hypertension, diabetes, CVD, cancer, COPD, who were not taking lipid-lowering medications, and had serum creatinine ≤ 150 µmol/L (i.e., Subgroup A). Shaded regions are respective 95% CIs and dotted lines show 95% prediction intervals of the summary curve estimated for Subgroup A. MD = mean difference from that calculated at the reference age (70 years). * = IPD meta-analysis of Subgroup A excluded data from three studies who did not provide IPD on COPD status (EMAS, FHS, SHIP).

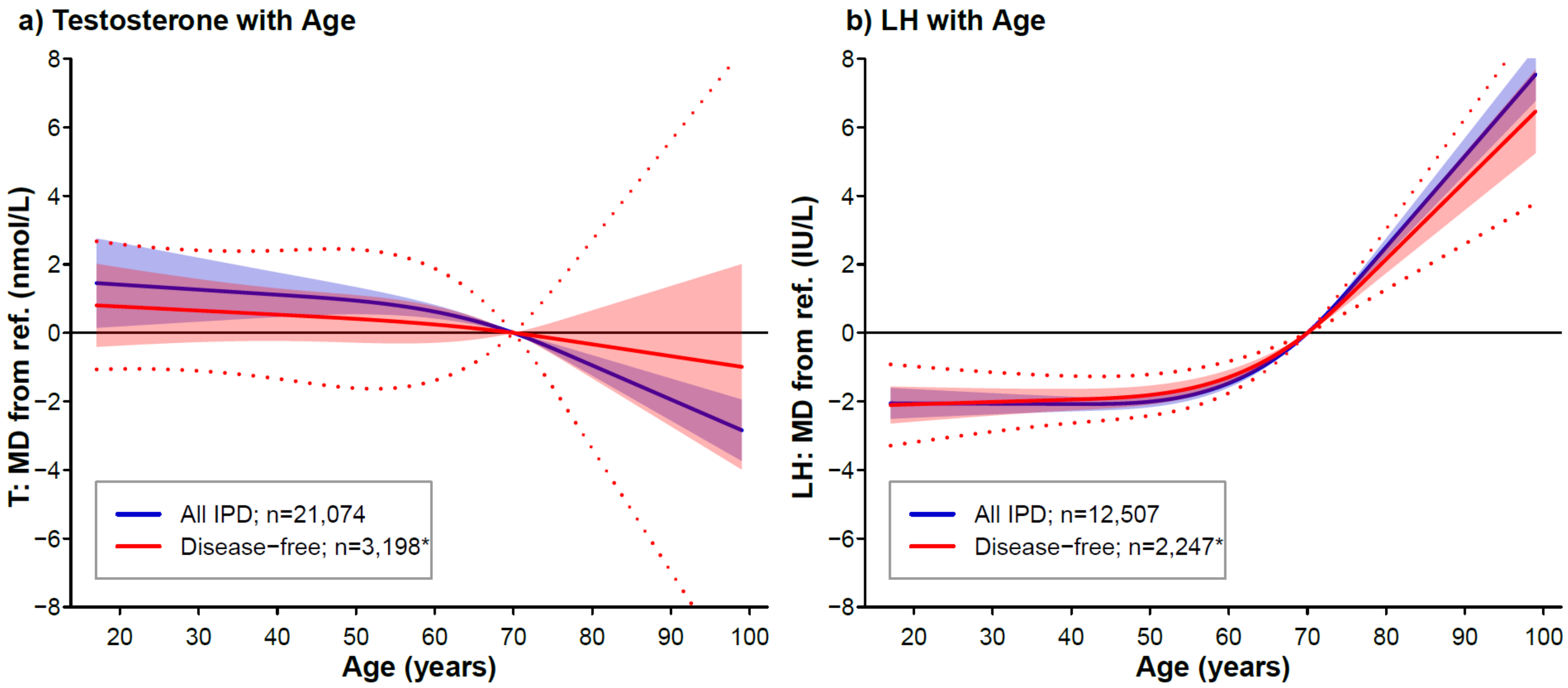

Figure S21. Summary curves for the associations of (a) baseline testosterone concentration (nmol/L) with baseline age and (b) baseline LH concentration (IU/L) with age, for all IPD versus IPD for men who were free of hypertension, diabetes, CVD, cancer, who were not taking lipid-lowering medications, and had serum creatinine ≤ 150 µmol/L (i.e., Subgroup B). Shaded regions are respective 95% CIs and dotted lines show 95% prediction intervals of the summary curve estimated for Subgroup B. MD = mean difference from that calculated at the reference age (70 years). * = COPD status was not used as a criterion to determine the subgroup of disease-free men in this analysis to ensure that IPD from all available studies could be used (because COPD status was not available from 3 of the studies who supplied IPD).

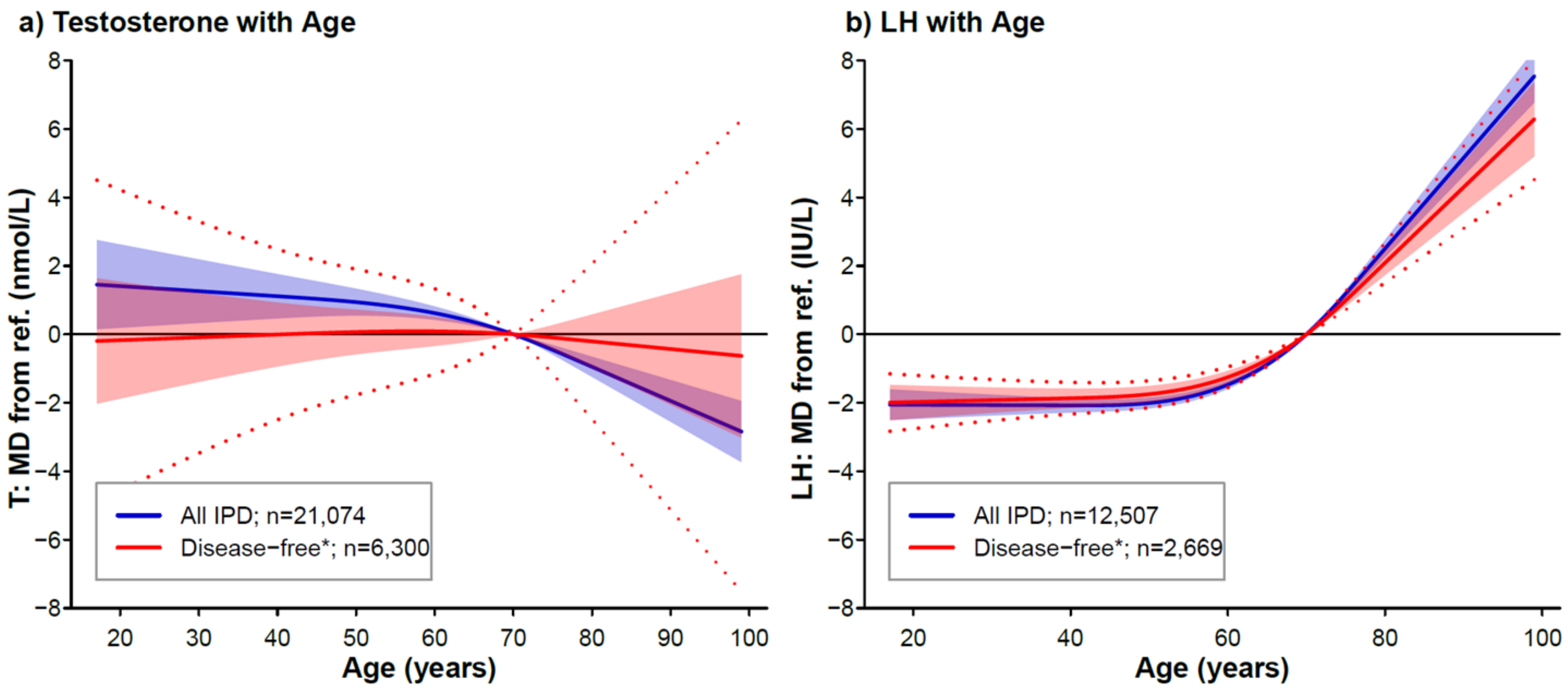

References cited

1. Marriott RJ, Harse J, Murray K, Yeap BB. Systematic review and meta-analyses on associations of endogenous testosterone concentration with health outcomes in community-dwelling men. *BMJ Open* 2021;11:e048013.
2. Wells GA, Shea B, O'Connell DO. The Newcastle-Ottawa scale (NOS) for assessing the quality of nonrandomised studies in meta-analyses, 2020. Available: http://www.ohri.ca/programs/clinical_epidemiology/oxford.asp [Accessed 10 Nov 2020].
3. Bartlett J, Keogh R, Bonneville EF. smcfcs: Multiple Imputation of Covariates by Substantive Model Compatible Fully Conditional Specification. 2022. R package version 1.6.1. https://CRAN.R-project.org/package=smcfcs
4. Seaman, SR., Bartlett JW, White IR. Multiple imputation of missing covariates with non-linear effects and interactions: An evaluation of statistical methods. BMC Medical Research Methodology 2012;12:46.
5. Bartlett JW, Morris TP. Multiple imputation of covariates by substantive-model compatible fully conditional specification. The Stata Journal 2015;15:437-456.
6. Grund S, Robitzsch A, Luedtke O. mitml: Tools for Multiple Imputation in Multilevel Modeling. R package version 0.4-3. 2021. https://CRAN.R-project.org/package=mitml
7. Quartagno M, Grund S, Carpenter J. jomo: A Flexible package for two-level joint modelling multiple imputation. The R Journal 2019;11(2):205-228.
8. Audigier V, White IR, Jolani S, et al. Multiple imputation for multilevel data with continuous and binary variables. Statistical Science 2018;3:160-183.
9. Shores MM, Biggs ML, Arnold AM, et al. Testosterone, dihydrotestosterone, and incident cardiovascular disease and mortality in the cardiovascular health study. J Clin Endocrinol Metab 2014;99:2061–8.
10. Riley RD, Tierney JF, Steward LA. Individual Participant Meta-Analysis: A Handbook for Healthcare Research. Oxford: John Wiley & Sons Ltd, 2021.
11. Harrell, FE Jr Regression Modelling Strategies: With Applications to Linear Models, Logistic and Ordinal Regression, and Survival Analysis (2nd edition). New York: Springer, 2015.
12. Gasparrini A, Armstrong B, Kenward MG, Multivariate meta-analysis for non-linear and other multi-parameter associations. Statistics in Medicine. 2012;31:3821-3839.
13. Sera F, Armstrong B, Blangiardo M, Gasparrini A. An extended mixed-effects framework for meta-analysis. Statistics in Medicine. 2019;38:5429-5444.
14. Balduzzi S, Rücker G, Schwarzer G. How to perform a meta-analysis with R: a practical tutorial. Evid Based Ment Health 2019;22:153-160.
15. Higgins JPT, Thompson SG. Quantifying heterogeneity in meta-analysis. Statistics in Medicine 2002;21:1539–58.
16. Borenstein M, Hedges LV, Higgins JPT, et al. Introduction to meta-analysis. West Sussex: John Wiley & Sons Ltd, 2009.
17. von Hippel PT. The heterogeneity statistic I^2 can be biased in small meta-analyses. BMC Medical Research Methodology 2015;15:35.
18. Viechtbauer W. Confidence intervals for the amount of heterogeneity in meta-analysis. Statistics in Medicine 2007;26(1):37–52.
19. Yeap BB, Marriott RJ, Antonio L, et al. Sociodemographic, lifestyle and medical influences on serum testosterone and sex hormone-binding globulin in men from UK Biobank. Clinical Endocrinology 2021;94:290–302.

20. Yeap BB, Marriott RJ, Adams RJ, et al. Androgens in men study (AIMS): protocol for meta-analyses of individual participant data investigating associations of androgens with health outcomes in men. BMJ Open 2020;10:e034777.
21. Gunnell AS, Knuiman MW, Divitini ML, Cormie P. Leisure time physical activity and long-term cardiovascular and cancer outcomes: the Busselton Health Study. Eur J Epidemiol. 2014;29:851-7.
22. LeBlanc ES, Wang PY, Janowsky JS, Neiss MB, Fink HA, Yaffe K, et al. Association between sex steroids and cognition in elderly men. Clin Endocrinol (Oxf). 2010;72(3):393-403.
23. Egger M, Davey Smith G, Schneider M, Minder CE. Bias in meta-analysis detected by a simple, graphical test. Br Med J 1997;315:629-34.
24. Sterne JAC, Egger M. Funnel plots for detecting bias in meta-analysis: guidelines on choice of axis. J Clin Epidemiol. 2001;54:1046-55.
25. Sterne JAC, Sutton AJ, Ioannidis JPA, et al. Recommendations for examining and interpreting funnel plot asymmetry in meta-analyses of randomised controlled trials. BMJ 2011;343:d4002.
26. Duval S, Tweedie R. Trim and fill: a simple funnel-plot-based method of testing and adjusting for publication bias in meta-analysis. Biometrics. 2000:56;455–63.
27. Andresen EM, Carter WB, Malmgren JA, Patrick DL. Screening for depression in well older adults: evaluation of a short form of the CES-D. Am J Prev Med. 1994;10(2):77-84.
28. Beck AT, Steer RA, Brown GK. Manual for the Beck Depression Inventory-II. San Antonio, TX: Psychological Corporation; 1996.
29. Prospective Studies Collaboration. Blood cholesterol and vascular mortality by age, sex, and blood pressure: a meta-analysis of individual data from 61 prospective studies with 55,000 vascular deaths. Lancet 2007;370:1829-39.
30. Shield KD, Kehoe T, Gmel G, Rehm MX, Rehm J. Societal burden of alcohol. In: Anderson P, Møller L, Galea G, editors. Alcohol in the European Union Consumption, harm and policy approaches: World Health Organisation; 2012. p. 10-28.
31. World Health Organisation (WHO). Global Recommendations on Physical Activity for Health. 2011.
32. Ainsworth BE, Haskell WL, Whitt MC, Irwin ML, Swartz AM, Strath SJ, et al. Compendium of physical activities: an update of activity codes and MET intensities. Med Sci Sports Exerc. 2000;32(9 Suppl.): S498-S504.
33. Washburn RA, Smith KW, Jette AM, Janney CA. The physical activity scale for the elderly (PASE): development and evaluation. J Clin Epidemiol. 1993;46(2):153-62.
34. Srinath R, Hill Golden S, Carson KA. Endogenous Testosterone and its Relationship to Preclinical and Clinical Measures of Cardiovascular Disease in the Atherosclerosis Risk in Communities Study. J Clin Endocrinol Metab 2015;100(4):1602-02.
35. Srinath R, Gottesman RF, Hill Golden S, et al. Association Between Endogenous Testosterone and Cerebrovascular Disease in the ARIC Study (Atherosclerosis Risk in Communities). Stroke 2016;47(11):2682-88.
36. Knuiman MW, Jamrozik K, Welborn TA, et al. Age and secular trends in risk factors for cardiovascular disease in Busselton. Aust J Public Health 1995;19:375-82.
37. Bhasin S, Pencina MJ, Kaur Jasuja G, et al. Reference ranges for testosterone in men generated using liquid chromatography tandem mass spectrometry in a community-base sample of healthy nonobese young men in the Framingham Heart Study and applied to three geographically distinct cohorts. J Clin Endocrinol Metab 2011;96(8):2430-39.
38. Norman PE, Flicker L, Almeida OP, et al. Cohort profile: the health in men study (HIMS). Int J Epidemiol 2009;38:48-52.

39. Chan YX, Alfonso H, Chubb SA, et al. Higher Dihydrotestosterone Is Associated with the Incidence of Lung Cancer in Older Men. Horm Cancer 2017;8(2):119-26.
40. Martin S, Haren M, Taylor A, et al. Cohort profile: The Florey Adelaide Male Ageing Study (FAMAS). Int J Epidemiol 2007;36:302-306.
41. Orwoll E, Babich Blank J, Barrett-Connor E, et al. Design and baseline characteristics of the osteoporotic fractures in men (MrOS) study - A large observational study of the determinants of fracture in older men. Contemp Clin Trials 2005;26:569–85.
42. Kische H, Gross S, Wallaschofski H, et al. Associations of androgens with depressive symptoms and cognitive status in the general population. PLoS ONE 2017;12(5):e0177272.
43. Hsu B, Cumming RG, Waite LM, et al. Longitudinal Relationships between Reproductive Hormones and Cognitive Decline in Older Men: The Concord Health and Ageing in Men Project. J Clin Endocrinol Metab 2015;100(6):2223-30.
44. Hsu B, Cumming RG, Naganathan V, et al. Temporal Changes in Androgens and Estrogens Are Associated With All-Cause and Cause-Specific Mortality in Older Men. J Clin Endocrinol Metab 2016;101(5):2201-10.
45. Hsu B, Cumming RG, Blyth FM, et al. Evaluating Calculated Free Testosterone as a Predictor of Morbidity and Mortality Independent of Testosterone for Cross-sectional and 5-Year Longitudinal Health Outcomes in Older Men: The Concord Health and Ageing in Men Project. J Gerontol A Biol Sci Med Sci 2018;73(6):729-36.
46. Cumming RG, Handelsman D, Seibel MJ, et al. Cohort Profile: The Concord Health and Ageing in Men Project (CHAMP). Int J Epidemiol 2009; 38: 374-8.
47. Tivesten Å, Vandenput L, Labrie F, et al. Low serum testosterone and estradiol predict mortality in elderly men. J Clin Endocrinol Metab 2009;94:2482-8.
48. Lopez DS, Lee W-C, Garcia CO, et al. Low testosterone and high cholesterol levels in relation to all-cause, cardiovascular disease, and cancer mortality in White, Black, and Hispanic men: NHANES 1988-2015. Hormones 2022;21:399-411.
49. Qiao Y, Liu F, Peng Y, et al. Association of serum Klotho levels with cancer and cancer mortality: Evidence from National Health and Nutrition Examination Survey. Cancer Med 2023;12:1922-34.
50. Liu P, Liu X, Wei D, et al. Associations of serum androgens with coronary heart disease and interaction with age: the Henan Rural Cohort Study. Nutr Metab Cardiovasc Dis 2021;31:3352-8.
51. Liu P, Wei D, Nie L, et al. The correlation between testosterone and stroke and the mediating role of blood pressure: the Henan Rural Cohort Study. J Stroke Cerebrovasc Dis 2022;31:106669.
